# Adsorption and activation of molecular oxygen over atomic copper(I/II) site on ceria

**DOI:** 10.1038/s41467-020-17852-8

**Published:** 2020-08-11

**Authors:** Liqun Kang, Bolun Wang, Qiming Bing, Michal Zalibera, Robert Büchel, Ruoyu Xu, Qiming Wang, Yiyun Liu, Diego Gianolio, Chiu C. Tang, Emma K. Gibson, Mohsen Danaie, Christopher Allen, Ke Wu, Sushila Marlow, Ling-dong Sun, Qian He, Shaoliang Guan, Anton Savitsky, Juan J. Velasco-Vélez, June Callison, Christopher W. M. Kay, Sotiris E. Pratsinis, Wolfgang Lubitz, Jing-yao Liu, Feng Ryan Wang

**Affiliations:** 1 Department of Chemical Engineering, University College London, Roberts Building, Torrington Place, London, WC1E 7JE UK; 2 Laboratory of Theoretical and Computational Chemistry, Institute of Theoretical Chemistry, Jilin University, Changchun, Jilin 130023 P. R. China; 3 Institute of Physical Chemistry and Chemical Physics, Slovak University of Technology in Bratislava, Faculty of Chemical and Food Technology, Radlinského 9, 81237 Bratislava, Slovak Republic; 4 Particle Technology Laboratory, Institute of Process Engineering, Department of Mechanical and Process Engineering, ETH Zürich, 8092, Zürich, Switzerland; 5 Diamond Light Source Ltd., Harwell Science and Innovation Campus, Chilton, Didcot, OX11 0DE UK; 6 School of Chemistry, University of Glasgow, Joseph Black Building. University Avenue, Glasgow, G12 8QQ UK; 7 Electron Physical Science Imaging Center, Diamond Light Source Ltd., Didcot, OX11 0DE UK; 8 Department of Materials, University of Oxford, Parks Road, Oxford, OX1 3PH UK; 9 College of Chemistry and Molecular Engineering, Peking University, Beijing, P. R. China; 10 Department of Materials Science and Engineering, National University of Singapore, Singapore, 117575 Singapore; 11 HarwellXPS—The EPSRC National Facility for Photoelectron Spectroscopy, Research Complex at Harwell (RCaH), Didcot, OX11 0FA UK; 12 Max-Planck-Institut Für Chemische Energiekonversion, Stiftstrasse 34-36, D-45470 Mülheim an der Ruhr, Germany; 13 Department of Physics, Technical University of Dortmund, 44221 Dortmund, Germany; 14 Fritz-Haber-Institut der Max-Planck-Gesellschaft, Faradayweg 4-6, 14195 Berlin, Germany; 15 UK Catalysis Hub, Research Complex at Harwell (RCaH), Rutherford Appleton Laboratory, Harwell, OX11 0FA UK; 16 London Centre for Nanotechnology, University College London, 17-19 Gordon Street, London, WC1H 0AH UK; 17 Department of Chemistry, University of Saarland, 66123 Saarbrücken, Germany

**Keywords:** Heterogeneous catalysis, Reaction kinetics and dynamics

## Abstract

Supported atomic metal sites have discrete molecular orbitals. Precise control over the energies of these sites is key to achieving novel reaction pathways with superior selectivity. Here, we achieve selective oxygen (O_2_) activation by utilising a framework of cerium (Ce) cations to reduce the energy of 3*d* orbitals of isolated copper (Cu) sites. Operando X-ray absorption spectroscopy, electron paramagnetic resonance and density-functional theory simulations are used to demonstrate that a [Cu(I)O_2_]^3−^ site selectively adsorbs molecular O_2_, forming a rarely reported electrophilic η^2^-O_2_ species at 298 K. Assisted by neighbouring Ce(III) cations, η^2^-O_2_ is finally reduced to two O^2−^, that create two Cu–O–Ce oxo-bridges at 453 K. The isolated Cu(I)/(II) sites are ten times more active in CO oxidation than CuO clusters, showing a turnover frequency of 0.028 ± 0.003 s^−1^ at 373 K and 0.01 bar *P*_CO_. The unique electronic structure of [Cu(I)O_2_]^3−^ site suggests its potential in selective oxidation.

## Abstract (web summary)

Precise control over the energy of atomic metal sites is key to unlocking novel reaction pathways. Here, the authors achieve selective oxygen activation by the isolated copper site on ceria, due to its reduced 3*d* orbital energy via cerium induced electron withdrawing effect.

## Introduction

Molecular O_2_ is the simplest and most abundant oxidant for combustion, oxidation and electrochemical reactions. The O_2_ activation pathway depends on the nature of the catalytic active site, which often involves *d*-block metals due to their rich oxidation states and variable coordination geometries^1^. In an electrochemical oxygen reduction reaction, an optimal position for the *d*-band centre of the metal is required for both adsorption of O_2_ and cleavage of the O=O bond^2^. Hence, group 10 metals and alloys, such as Pt and Pd, are a standard choice^3–5^. In comparison, when considering selective oxidation, metals with lower *d*-band centres, such as group 11 metals, are preferred^6–8^. Typically, O_2_ weakly adsorbs on these metal surfaces, forming superoxide O_2_^−^ and peroxide O_2_^2−^. Gold and its alloys with Pd can form H_2_O_2_ in situ and subsequently activate the C–H bond^9,10^ while large Ag nanoparticles are the commercial catalyst for the epoxidation of ethylene^11^. The first group 11 metal, Cu, has a higher *d*-band centre than both Au and Ag^12,13^. As a result, Cu is mainly used for the total oxidation of CO^14–16^ and NH_3_ oxidation with NO^17–19^. The redox reaction of Cu(I)/Cu(II) involves a transition between *d*^10^ and *d*^9^ electron configurations, which is rarely seen in the periodic table and is key to the high oxidation activity and selectivity. Unlike Au and Ag, the Cu based peroxide O_2_^2−^ and superoxide O_2_^−^ systems are mainly found in homogeneous catalysis^20–22^, where 1:1 Cu:(η^2^–O_2_) single-site complexes are present in nature or synthetically made for Cu-mediated catalysis^23–25^.

**Fig. 1:**
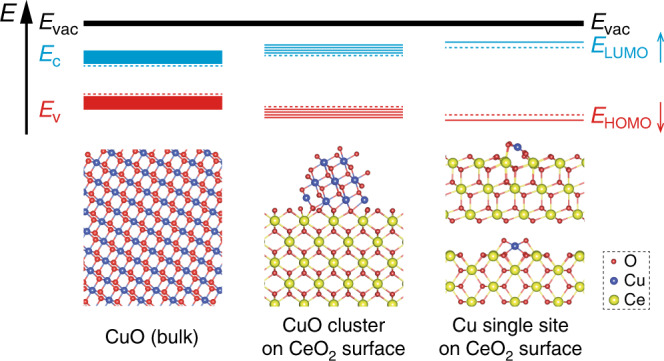
Schematic of electron withdrawing effect from Ce^4+^. From bulk CuO to atomic Cu(I/II) site, continuous band structure becomes discrete molecular orbitals. *E*_vac_, *E*_c_, *E*_v_, *E*_LUMO_ and *E*_HOMO_ represent for the energy level of vacuum, conductive band, valence band, LUMO and HOMO, respectively. The HOMO and LUMO gap increased in comparison between bulk and atomic Cu(I/II) site. This is indicated by the study of occupied 3*d* orbitals and unoccupied 4*p* orbitals energy levels, respectively. The schematic structures illustrate the atomic Cu(I/II) site concept and do not represent the real surface species.

The surface atomic metal site has discrete electronic structures compared with the continuous band structures found in clusters^26,27^. Their interactions with reactants are based on their structure and energy match. The supporting cation, Ce^4+^, can withdraw electrons from Cu to reduce its *d*-band centre, enhancing the electrophilicity of the Cu species^28^. This electron withdrawing effect is strengthened by increasing the number of Ce^4+^ ions per Cu site. The energy splitting between the highest occupied molecular orbital (HOMO) and the lowest unoccupied molecular orbital (LUMO) of Cu species is therefore increased by the interaction between Cu and Ce (Fig. 1), and is maximised in isolated and atomically dispersed Cu sites, which have the lowest possible HOMO. Recently, such a discrete electronic structure was demonstrated in the Ag/Cu system, in which the Cu *d* states were nearly unperturbed from their free-atom state^29^. In the Cu/TiO_2_ system, the atomic Cu site can reversibly modulate the macroscopic optoelectronic properties of TiO_2_, enhancing the photocatalytic hydrogen evolution activity^30^. Thus, the ability to precisely control the electronic structures of atomic sites is important to achieve the desired reaction pathway.

As part of our search for catalytic active centres in selective O_2_ adsorption and activation towards electrophilic or nucleophilic oxygen species^31,32^, here we report controlling the electronic structures of atomic Cu(I)/Cu(II) sites via CeO_2_ surface. The neighbouring Ce^4+^ lowers the occupied 3*d* orbital energy of atomically isolated Cu(II) by 0.7 eV, as shown in results from X-ray absorption near edge structure (XANES). In comparison, the energy of the unoccupied 4*p*_*z*_ orbitals increases by 2.2 eV from CuO clusters to atomic Cu(II) site. The change in the electronic structure leads to the formation of an electrophilic [Cu(II)O_2_(η^2^–O_2_)]^4−^ site upon O_2_ adsorption, as confirmed via near ambient pressure-near edge X-ray absorption fine structure (NAP-NEXAFS) and Raman spectroscopy supported by spin-polarised density functional theory (DFT) calculations. With quantified density of the surface atomic Cu(II) at 1 site per 5 nm^2^ by electron paramagnetic resonance (EPR) spectroscopy, the activity of atomic Cu(I/II) site in model CO oxidation is directly correlated to its quantitative electronic structures.

## Results

### Identifying the highest density of atomic Cu(II) site

**Fig. 2:**
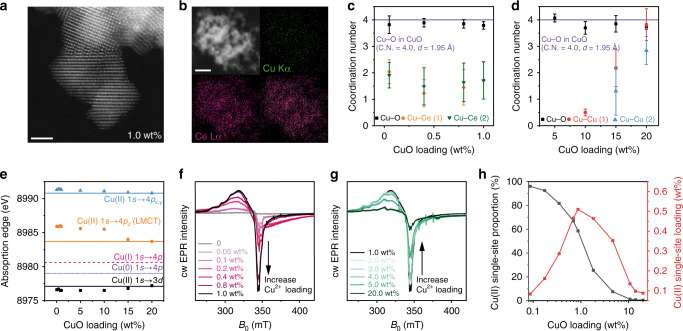
Structure and quantification of atomic Cu(II) site. **a** HAADF-STEM images of CuO–CeO_2_ at 1 wt% CuO loading. **b** EDS mapping of the CuO-CeO_2_ at 1 wt% CuO loading with red for Ce and green for Cu. **c**, **d** The coordination numbers (C.N.) of Cu–O, Cu–Ce(1), Cu–Ce(2), Cu–Cu(1) and Cu–Cu(2) scattering as a function of Cu loading, derived from the EXAFS spectra. Purple line: The Cu–O coordination number is 4 in the CuO standard. The standard error of the mean (s.e.m.) is labelled according to the EXAFS fitting results listed in Supplementary Table 1. **e** The peak position of 1*s* → 3*d* and 1*s* → 4*p* electron transitions in the first derivative of Cu K-edge XANES, showing changes of absorption energy as a function of Cu loading compared to metallic Cu (purple line), Cu_2_O (pink line) and CuO (black, orange and blue lines) as standard materials. **f** X-band cw EPR spectra for CuO–CeO_2_ with CuO loading from 0 to 1 wt%. **g** EPR spectra for CuO–CeO_2_ with CuO loading from 1 to 20 wt%. **h** Quantification of atomic Cu(II) site. Black: molar proportion of atomic Cu(II) site within all Cu species in CuO–CeO_2._ Red: absolute loading of atomic Cu(II) site showing the optimal content at 1 wt%. Scale bars: 2 nm in (**a**) and 10 nm in (**b**).

FSP is used to obtain atomic Cu(II) site and clusters supported on CeO_2_ nanoparticles with small particle sizes (3–5 nm) (Supplementary Figs. 1 and 2) and high surface area up to 220 m^2^/g^33,34^. The pyrolysis of Cu and Ce forms uniformly distributed Cu species on CeO_2_ owing to simultaneous CeO_2_ crystallisation and Cu site formation. Due to the low Z-contrast of Cu compared with Ce, Cu species are difficult to observe in high resolution aberration-corrected high angle annular dark field-scanning transmission electron microscopy (HAADF-STEM) (Fig. 2a). The presence of Cu is confirmed by energy-dispersive X-ray spectroscopy (EDS) (Cu peak in Supplementary Fig. 2b). Element mapping shows a uniform distribution of Cu and Ce at 1 wt% CuO loading (Fig. 2b). Increasing the CuO loading causes aggregation of Cu species (white circle in Supplementary Fig 3c, d). Synchrotron X-ray diffraction (SXPD) shows the presence of small crystalline CuO particles in 20 wt% CuO–CeO_2_ (Supplementary Fig. 4).

The structure of atomic Cu site is revealed in the EXAFS study. Below 1 wt% CuO loading, the EXAFS show a similar Cu–O scattering at 1.95 ± 0.01 Å with coordination numbers between 3.70 ± 0.24 and 4.07 ± 0.15 (Fig. 2c; Supplementary Fig. 6; Supplementary Table 1). There is no Cu–Cu scattering, indicating the atomic isolation of Cu sites. Above 10 wt%, Cu–Cu scattering appears at 2.87 ± 0.04 Å (Cu–Cu(1)) and 3.12 ± 0.02 Å (Cu–Cu(2)) (Fig. 2d; Supplementary Table 1), corresponding well to the scattering in standard CuO crystals (Supplementary Table 1). At the near edge, XANES shows the 1*s* → 3*d* quadruple-allowed transitions for all samples from 20 wt% to 0.05 wt% CuO loading, suggesting a majority of Cu(II) species (Fig. 2e; Supplementary Fig. 5)^35,36^. The absorption energy of 1*s* → 3*d* transitions decreases from 8977.2 eV at 20 wt% to 8976.5 eV below 1 wt%, while the CuO standard gives 8977.1 eV. The reduced adsorption energy at low loadings suggests a decrease of the 3*d* orbital energy for atomic Cu(II) site. The Cu(II) site has the *3d*^9^ configuration with four O^2−^ ligands. The absorption of 1*s* → 3*d* depends on the energy level of the half-empty orbital which is the only destination of the excited electron to 3*d* orbitals. This is considered to be the HOMO or the singly occupied molecular orbital. In comparison to CuO clusters and bulk CuO, the absorption energies for the 1*s* → 4*p*_*z*_ (i.e., the shakedown peak from ligand-to-metal charge transfer) and 1*s* → 4*p*_*xy*_ transition rise with less CuO loading, up to 2.2 and 0.5 eV, respectively, indicating an increase of the unoccupied 4*p* orbital energy for atomic Cu(II) site (Fig. 2e; Supplementary Fig. 5). High energy resolution fluorescence detected XANES (HERFD-XANES) is performed to validate such change of 1*s* → 3*d*, 1*s* → 4*p*_*xy*_ and 1*s* → 4*p*_*z*_ energy from clusters to atomic sites, showing the similar trend (Supplementary Fig. 5e, f; Supplementary Table 2). Thus, the decrease of CuO size leads to the stronger interaction between Cu–O–Ce and less interaction between Cu–O–Cu, increasing the gap between HOMO and LUMO. The change of white line position from 1 to 20 wt% shows the same trend as that of the 4*p* orbitals (Supplementary Table 2). The XANES study proves the assumption that Ce^4+^ reduces the HOMO of atomic Cu(II) site and increases the energy of its 4*p* orbitals (Fig. 1).

The Cu(II) has a *d*^9^ configuration and is, therefore, EPR active. With increasing Cu loading, the EPR spectra show signals characteristic of predominantly isolated Cu(II) species of an axial symmetry with *g*_||_ > *g*_⊥_ > *g*_e_ (Fig. 2f). The EPR spectra are representative for composite signals indicating atomic Cu(II) sites are in different coordination environments. The spectrum of 0.05 wt% CuO–CeO_2_, shows two resolved species with *g*_||_ = 2.327, *g*_⊥_ = 2.048, *A*_||_ = 372 MHz, *A*_⊥_ = 55 MHz and *g*_||_ = 2.293, *g*_⊥_ = 2.036, *A*_||_ = 402 MHz, *A*_⊥_ = 92 MHz (signals A1 and C1, respectively^37–40^, Supplementary Fig. 7; Supplementary Note 1; Supplementary Table 3), superimposed on a broader virtually isotropic line with 〈*g*〉 = 2.1 (signal B1). With increasing CuO loading, from 0.4 up to 1 wt%, a poorly resolved signal with extrema at *g* = 2.21 and 2.05 becomes dominant (Fig. 2f, and signal B2 in Supplementary Fig. 8)^16,41^. The Spin Hamiltonian parameters of the resolved signals A1 and C1, agree with a *d*_x2–y2_ electronic ground state assigned to isolated atomic Cu(II) site in tetragonally distorted octahedral due to Jahn–Teller effect and square planar coordination of oxygen ligands, respectively^16,41,42^. Signals B1 and B2, showing averaged 〈g〉 values close to the signal of C1, also originate from the Cu(II) with (distorted) square planar geometry. It is proposed that these signals stem from [Cu(II)O_4_]^6−^, which is supported by the XAFS data. The larger linewidth has been previously attributed to dipolar broadening effects in a Cu(II)-containing aggregated phase of an oxidative type^16,41^. However, since no zero field splitting is resolved in these spectra, the dipolar interactions are likely of a long-range character (with a Cu–Cu distance >8 Å), and such geometric arrangements are not detected in XAFS. The simulated EPR components indicate the presence of at least two types of isolated atomic Cu(II) sites with one additional site having long-range spin interactions. Such composite spin states, which are not distinguishable in X-ray based techniques, suggest a certain heterogeneity of surface structures.

Using dilute CuSO_4_·5H_2_O in Na_2_SO_4_ as an external standard^18,43^, we quantified the atomic Cu(II) site content as a function of the CuO loading with EPR spectroscopy (Fig. 2h). Nearly 100% of the Cu is in the form of atomic Cu(II) site at 0.05 and 0.1 wt% loading. The molar proportion of isolated Cu(II) reaches 58% at 1 wt% of CuO–CeO_2_, giving the highest absolute atomic site loading. Giving the surface area of 220 m^2^/g, a maximum density is calculated as 1 site per 5 nm^2^ CeO_2_ surface. The density is significantly reduced above 1 wt% of CuO–CeO_2_, and approaching 0 for 20 wt% CuO–CeO_2_.

### CO oxidation activity with atomic Cu site

**Fig. 3:**
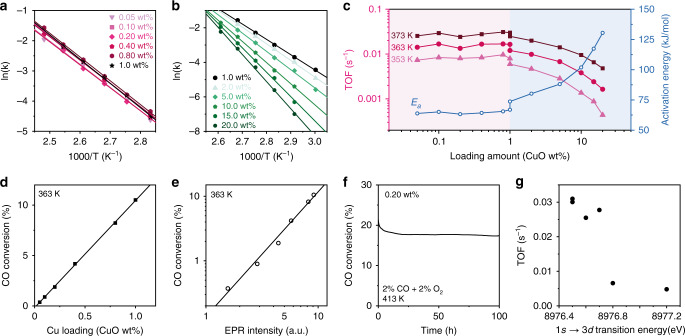
Catalytic evaluation of Cu species in CO oxidation. **a** Arrhenius plots at WHSV = 750,000 mL_CO_ h^−1^ g_CuO_^−^^1^ for Cu species ranging from 0.1 to 1 wt%. **b** Arrhenius plots at WHSV = 120,000 mL_CO_ h^−1^ g_CuO_^−1^ for Cu species ranging from 1 to 20 wt%. **c** TOF and *E*_a_ as function of Cu loading. WHSV = 750,000 mL_CO_ h^−1^ g_CuO_^−1^ and 120,000 mL_CO_ h^−1^ g_CuO_^−1^ were used below and above 1 wt% CuO loading, as indicated in the pink and blue areas, respectively. **d** Below 1 wt% CuO loading, CO conversion as a function of CuO loading amount at 363 K. **e** Below 1 wt% CuO loading, CO conversion as a function of the EPR intensity of atomic Cu(II) site at 363 K. **f** On stream stability test for 0.20 wt% CuO–CeO_2_ at 1,500,000 mL_CO_ h^−1^ g_CuO_^−1^ and 413 K. **g** The correlation of the CO oxidation TOF of CuO–CeO_2_ catalysts against their absorption energies of 1*s* → 3*d* transition.

With quantified atomic Cu(II) site loading, we investigate the relationship between the absolute atomic Cu(II) site content and the catalytic activity in model CO oxidation. We also compared the different catalytic behaviours between atomic Cu site and oligomers/clusters^44^. In kinetic studies, the ratio between the CO space velocity and CuO weight in the bed is fixed. The Arrhenius plots from 0.05 to 1 wt% loading fall into similar lines with nearly identical slopes (Fig. 3a) while those of catalysts from 1 to 20 wt% loading show a steady increase in slope (Fig. 3b). As a result, a similar activation energy (*E*_a_) of 62 ± 2 kJ mol^−1^ is obtained from 0.05 to 1 wt% beyond which the *E*_a_ increases from 62 to 130 kJ mol^−1^ at 20 wt% (Fig. 3c). Below 1 wt%, CO conversion has a near linear correlation with CuO loading and the EPR intensity of atomic Cu(II) site (Fig. 3d, e; Supplementary Fig. 9). This activity-spectroscopy relationship suggests that CO oxidation is promoted by atomic Cu site. The similar *E*_a_ below 1 wt% also indicates an atomic site catalytic behaviour, which is in agreement with the EPR quantification (Fig. 2h). The turnover frequency (TOF) at 353, 363 and 373 K as a function of CuO loading (Fig. 3c) shows similar values below 1 wt%. An average TOF of 0.028 ± 0.003 s^−1^ is obtained below 1 wt% at 373 K and 0.01 bar *P*_CO_. This is kinetic evidence that atomic Cu site is the main active species as a similar TOF is obtained regardless of CuO loading. The TOF achieved at 373 K is comparable with the atomic Pt(II) site on CeO_2_ in the literature^45^. Above 1 wt%, the TOF begins to drop and eventually falls below 0.001 s^−1^ at 353 K (Fig. 3c). Atomic Cu site is ten times more active than CuO clusters in these conditions. The increase of *E*_a_ and drop of the TOF above 1 wt% is due to the decrease of the atomic site density and formation of Cu clusters.

The atomic Cu(II) site are re-examined after catalysis. From 0.1 to 1 wt%, nearly identical EPR spectra are observed, (Supplementary Fig. 10) indicating that the chemical environment of atomic Cu(II) site stays the same. As a result, high catalytic stability is obtained with a conversion of 18% for 100 h (Fig. 3f). For 0.05 wt%, an increase of isolated components A1 and C1 is observed. This is associated with the decrease of the long range (>8 Å) coupled component B1, indicating a redistribution and further isolation of the atomic Cu(II) site during catalysis (Supplementary Fig. 7b).

The HOMO energy levels, which are consistent with the absorption energies of 1*s* → 3*d* transition in the Cu K edge XANES, are negatively correlated with TOF in CO oxidation activity (Fig. 3g). The lower HOMO energy level of Cu single-sites may lead to enhanced competence in O_2_ activation, which is then investigated via a series of in situ characterisation techniques below.

### Dynamics of O_2_ activation on atomic Cu site

**Fig. 4:**
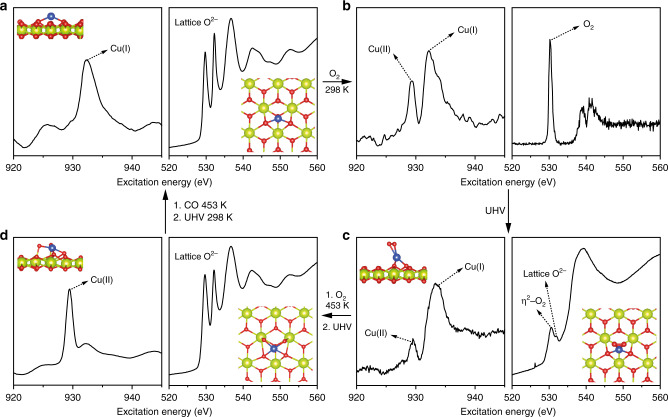
Activation of O_2_ at atomic Cu site. **a** DFT simulation of an atomic Cu(I) site over CeO_2_(111) surface and NEXAFS of 1 wt% CuO–CeO_2_ under UHV. The sample is treated with CO in situ under 453 K with subsequent cooling to 298 K under UHV. Left: Cu L_3_ edge; Right O K-edge, showing Cu(I) and lattice O, which is consistent with DFT simulation. **b** Upon O_2_ adsorption at 298 K, the Cu L_3_ edge reveals the formation of Cu(II) while O K-edge shows surface adsorbed O_2_. **c** Under UHV, the gas phase O_2_ is removed, leaving η^2^–O_2_ on the surface. The Cu(II) content is also reduced. **d** Upon heating to 453 K under O_2_ and then changing to UHV, the Cu is oxidised completely to Cu(II) while η^2^–O_2_ is either desorbed or forming lattice O^2−^.

The difference in catalytic behaviours between atomic Cu site and CuO clusters suggests different reaction mechanisms. We hypothesise that this is due to a change in 3*d* and 4*p* orbital energy levels in atomic Cu(II) site (Fig. 2e; Supplementary Fig. 5) caused by the neighbouring Ce^4+^. This can potentially change the O_2_ activation pathways during the reaction. NAP-NEXAFS and spin-polarised DFT simulations are performed to investigate the electronic structures of atomic Cu(II) site upon O_2_ adsorption. First, the DFT simulation shows that an atomic Cu(0) site is oxidised to Cu(I) (calculated effective charge *Q*_Cu_ = 0.62|e| at the (111) and 0.48|e| at the (110) surface), which coordinates with two lattice oxygen ions to form a [Cu(I)O_2_]^3−^ site (Fig. 4a inset, Supplementary Fig. 11a; Supplementary Tables 4 and 5). In the NEXAFS, a Cu(I) site is identified at the Cu L_3_ edge by in situ reduction under CO at 453 K with subsequent cooling to 298 K under ultrahigh vacuum (UHV) (Fig. 4a left)^46^. At the O K-edge, only the lattice O of CeO_2_ is observed (Fig. 4a right; Supplementary Fig. 12).

Upon O_2_ adsorption at 298 K, Cu(I) is partially oxidised to Cu(II) (Fig. 2b left) while the O K-edge of gaseous O_2_ (Fig. 4b right, 530.2 eV) is clearly different from that of the lattice O (Fig. 4a right, 529.7, 532.2 and 536.7 eV). UHV is then applied to remove gas phase O_2_ and any physically adsorbed O_2_ at the surface. The O K-edge NEXAFS shows two absorption peaks at 530.5 and 532.0 eV, respectively (Fig. 4c right). The former is a η^2^–O_2_ that is adsorbed on atomic Cu site, while the latter is the lattice O of CeO_2_ (Supplementary Fig. 12). In the meantime, the content of Cu(II) is reduced (Fig. 4c left)^47^. Raman spectroscopy of 1 wt% CuO–CeO_2_ reveals an O–O stretch at 830 cm^−1^, confirming the presence of η^2^–O_2_ (Supplementary Fig. 13). The shoulder band around 600 cm^−1^ is attributed to Ce^3+^^48^, which is absent for the Raman spectra of 20 wt% CuO–CeO_2_ and pure CeO_2_. The 20 wt% CuO–CeO_2_, the CuO standard and the CeO_2_ standard do not show this O–O stretch, suggesting that the η^2^–O_2_ is associated with atomic Cu site. DFT simulations show the formation of an electrophilic species [Cu(II)O_2_(η^2^–O_2_)]^4−^ upon O_2_ adsorption (Fig. 4c inset; Supplementary Fig. 11b; Supplementary Tables 4 and5), which is consistent with the NAP-NEXAFS and Raman results. The Cu–O bond lengths match the EXAFS data (Supplementary Tables 1, 4 and 5). The simulated O_2_ adsorption energy is −0.68, −1.25 and −0.03 eV for an atomic Cu site over CeO_2_(111), an atomic Cu site over CeO_2_(110) and the pure CeO_2_(111) surface, respectively, suggesting a strong chemical adsorption at atomic Cu site. In comparison, Cu coordinates with four surface lattice O^2−^ on CeO_2_(100) (Supplementary Fig. 14). O_2_ can only be physically adsorbed on such site with a distance at 3.01 Å based on DFT simulations. Such η^2^–O_2_ is not observed in the 20 wt% CuO–CeO_2_ (Supplementary Fig. 15).

Upon heating to 453 K under O_2_ with subsequent UHV, only lattice O is visible in the O K-edge NAP-NEXAFS, while almost all the Cu is oxidised to Cu(II) (Fig. 4d)^46^. The DFT simulations also indicate the cleavage of the O–O bond, forming a [Cu(II)O_4_]^6−^ site (Fig. 4d inset; Supplementary Fig. 11c). The calculated charge per O_ads_ atom decreases from −0.22 to −0.595 (Supplementary Table 8). The calculated barrier from [Cu(II)O_2_(η^2^–O_2_)]^4−^ to [Cu(II)O_4_]^6−^ is 1.41 eV (Supplementary Fig. 16). In comparison, decomposition of O_2_ on Cu_2_O(111) surface requires an energy barrier of 1.13 eV (Supplementary Fig. 17). The final state with fully dissociated O atoms is 0.3 eV more stable than the initial undissociated O_2_, whereas the final state of oxygen on Cu single site is 1.05 eV less stable than its initial state. These results suggest the dissociation of O_2_ on Cu_2_O clusters is easier than that on Cu single sites. The [Cu(II)O_4_]^6−^ can be further converted back to [Cu(II)O_2_(η^2^–O_2_)]^4−^ by just reducing the temperature to 298 K, suggesting the reversible conversion between these two Cu(II) sites (Supplementary Fig. 18). In the last step, CO reduces [Cu(II)O_4_]^6−^ at 453 K, to resume the original [Cu(I)O_2_]^3−^. Bader charge analysis is calculated to analyse the charge transfer between Cu(I/II) sites and Ce^4+^ on the CeO_2_(111) surface. In both [Cu(I)O_2_]^3−^ and [Cu(II)O_4_]^6−^ sites, a charge transfer from Cu at the value of +0.617 and +1.074 is observed, respectively. Ce receives charge at the value of −0.524 and −0.316 for 27 Ce^4+^ ions that participate in the calculation, respectively (Supplementary Figs. 19 and 20; Supplementary Tables 6–8). Based on the same calculations parameters for the Cu single-sites, the calculated Bader charges of Cu_2_O(111) and CuO(111) surface are +0.495 and +1.001, respectively (Supplementary Table 9). Compared with pure copper oxides, both Cu(I) and Cu(II) single sites on CeO_2_ are more positively charged. The increase of electron density of Ce in both models proves the role of Ce^4+^ as an electron acceptor for the atomic Cu(I/II) sites.

The O_2_ activation over [Cu(I)O_2_]^3−^ site can be understood by [Cu(I)O_2_]^3−^ + O_2_ + Ce^3+^ → [Cu(II)O_2_(η^2^–O_2_)]^4−^ + Ce^4+^ (Fig. 4a–c; Supplementary Fig. 21). One Ce^3+^ is required to donate one electron form the η^2^–O_2_ species. During heating at 453 K, η^2^–O_2_ is formally reduced via [Cu(II)O_2_(η^2^–O_2_)]^4−^ + 2Ce^3+^ → [Cu(II)O_4_]^6−^ + 2Ce^4+^ (Fig. 4c–d). We hypothesise that another two Ce^3+^ are required to balance the charges. Finally, during CO oxidation, [Cu(II)O_4_]^6−^ + 2CO + 3 Ce^3+^ → [Cu(I)O_2_]^3−^ + 2CO_2_ + 3Ce^4+^. Only a small amount of Ce participates the reaction for 1 wt% CuO–CeO_2_ catalysts, which is then difficult to detect via in situ spectroscopy. In addition to O_2_, activation of CO and the mobility of lattice O in CeO_2_ are also important for the CO oxidation. CO temperature programmed reduction and desorption are performed to compare the activation of CO with Cu single-site and clusters. The atomic Cu(II) sites are more active towards CO adsorption and oxidation than the majority of CuO sites on clusters as reflected by the shift of the reduction peak from 451 to 380 K (Supplementary Fig. 22a). The Cu(II) site shows the main desorption peak at 374 K, which is 6 K lower than that of the CuO clusters (Supplementary Fig. 22b). Higher Ce^3+^ content^49^ is found in the 1 wt% CuO–CeO_2_ comparing with 20 wt% CuO–CeO_2_ (Supplementary Fig. 23; Supplementary Table 10), which agrees with the shoulder band at 600 cm^−1^ in the Raman spectrum (Supplementary Fig. 13). According to the MvK mechanism, high Ce^3+^ content suggests more oxygen vacancy formation^50^, and thus improve the mobility of lattice O^51^. Therefore, the presence of the atomic Cu site promotes the O_2_ and CO activation and the lattice O mobility, leading to higher CO oxidation activity compared with CuO clusters (Fig. 3c).

### The role of Cu(I/II) single-sites in CO oxidation

The NAP surface experiments and DFT simulations identify the presence and transformation of [Cu(I)O_2_]^3−^, [Cu(II)O_2_(η^2^–O_2_)]^4^^−^ and [Cu(II)O_4_]^6^^−^ sites at low pressure. We hypothesise that they are the intermediate states under the CO oxidation conditions. We first aim to identify the [Cu(I)O_2_]^3−^ during the reaction under a reductive atmosphere.

**Fig. 5:**
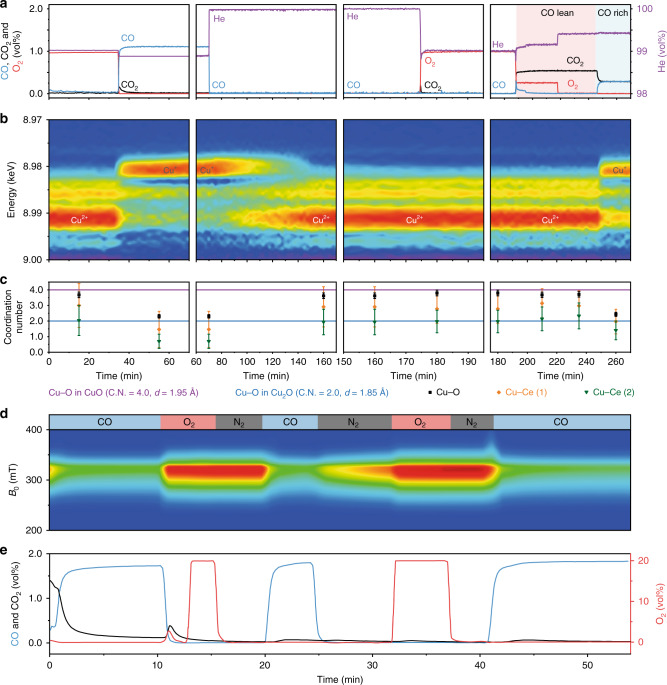
Operando XAFS at the Cu K-edge and EPR study of 1 wt% CuO–CeO_2_. **a** Gas concentration at the outlet of operando XAFS reactor as a function of time. **b** Contour map of the first derivative XANES spectra, showing the continuous changes of absorption edge fine structure. **c** Corresponding change of coordination number in Cu–O, Cu–Ce (1) and Cu–Ce (2) scattering as a function of time. **d** Contour map of the first integral of EPR intensity of atomic Cu(II) site as a function of time. **e** The corresponding gas concentrations.

The XAFS (Fig. 5a–c)/EPR (Fig. 5d) spectra and outlet concentration of CO, CO_2_ and O_2_ are recorded simultaneously. Under air and N_2_, EPR observes a low content of a well-defined isolated Cu(II) (*g*_||_ = 2.275, *g*_⊥_ = 2.050, *A*_||_ = 492 MHz, *A*_⊥_ = 34 MHz; signal C2 in Supplementary Fig. 8) that is highly sensitive to oxygen. Supplying 2% CO lowers the dominant EPR signal of atomic Cu(II) site, suggesting a reduction of Cu(II) (Fig. 5d). Simulations of the difference between EPR spectra recorded under CO and under air (signal B2 in Supplementary Fig. 8), indicate that the Cu(II) species coordination is of rhombic symmetry (*g*_z_ = 2.28, *g*_y_ = 2.132, *g*_x_ = 2.051, *A*_z_ = 472 MHz, *A*_x,y_ = 35 MHz) and compatible with a tetrahedral distorted square-planar ligand sphere. The corresponding XANES spectra show that the reduction reached and maintained Cu(I) without further reduction to Cu(0) (Fig. 5b). No Cu–Cu scattering is found in the EXAFS, suggesting a stable atomic Cu(I) site species rather than Cu_2_O clusters. The EXAFS spectra are fitted with a corrected Debye–Waller factor for the temperature of the reaction (Supplementary Fig. 24). The coordination number of Cu–O decreases from 3.93 ± 0.18 to 2.03 ± 0.11, while the bond distance is maintained at 1.90 ± 0.01 Å (Fig. 5c; Supplementary Figs. 25 and 26; Supplementary Table 11). The Cu–O bond distance agrees well with the DFT model of the [Cu(I)O_2_]^3−^ site on CeO_2_ surface (Supplementary Tables 4 and 5). As the Cu–O bond is not elongated to match the Ce–O bond length (2.38 Å) in bulk CeO_2_^52^, such atomic Cu sites are not likely to be stabilised by substituting Ce atoms in CeO_2_ lattice.

The [Cu(I)O_2_]^3−^ is stable under a reductive atmosphere, corroborating the DFT simulations in which a Cu(0) atom is immediately oxidised to Cu(I) by Ce^4+^ at the CeO_2_(111) surface (Fig. 4a; Supplementary Fig. 27). In comparison with CO atmosphere, atomic Cu(II) site is stable under H_2_ at 453 K (Supplementary Fig. 28), due to the lack of a metallic surface for the dissociation of H–H bond. XANES of 1 wt% CuO–CeO_2_ shows weaker absorption from 1*s* → 4*p* transition than that of 20 wt% and bulk Cu_2_O (Supplementary Fig. 29). The absorption energy of 1*s* → 4*p* transitions increases from 8980.6 eV for bulk Cu_2_O and Cu_2_O clusters at 20 wt% to 8980.9 eV for [Cu(I)O_2_]^3−^ at 1 wt%, respectively, indicating an increase of the 4*p* orbital energy level relative to the 1*s* orbital for [Cu(I)O_2_]^3^^−^ site. DFT calculation shows that the Cu(I) single-site on CeO_2_(111) has significantly lower valence band maximum compared with Cu_2_O(111) surface (−0.9451 eV vs. −0.2244 eV, Supplementary Table 12). Such a 0.7207 eV difference from calculations of Cu(I) species is very close to that of the experimentally measured difference in 1*s* → 3*d* transition energy of Cu(II) species (0.6 eV, Fig. 2e). This result further validates that both Cu(I) and Cu(II) single-sites on CeO_2_ have lower HOMO energy compared with Cu clusters.

Ultrapure N_2_ was introduced to remove the reducing CO. A slow increase in paramagnetic Cu(II) is observed in the EPR (Fig. 5d; Supplementary Fig. 8) and XANES (Fig. 5b), suggesting oxidation of Cu(I) by Ce^4+^^53^. As a result, increased coordination numbers of in Cu–O, Cu–Ce(1) and Cu–Ce(2) from 2.03 ± 0.11, 1.46 ± 1.16 and 0.70 ± 0.46 to 3.62 ± 0.18, 2.91 ± 1.29 and 1.94 ± 0.82 are found (Fig. 5c, Supplementary Figs. 25 and 26; Supplementary Table 11).

The CO oxidation is carried out at CO:O_2_ ratios of 1:1, 2:1 and 4:1, respectively. From the oxidative reaction condition at 1:1 to the reductive condition at 4:1, a reduction of Cu(II) to [Cu(I)O_2_]^3−^ is found and associated with the decrease of coordination numbers of Cu–O, Cu–Ce(1) and Cu–Ce(2) (Fig. 5b, c). Such a structural evolution of atomic Cu site under different reaction environments has also been reported for the Pt site system recently^54^. The comparison among the TOFs obtained with various Cu-based and Pt-based catalysts in CO oxidation shows that the atomic Cu sites in 1 wt% CuO–CeO_2_ is more active than the reported Cu clusters catalysts and the activity is comparable with that of the isolated Pt sites on CeO_2_^45^ (Supplementary Table 13; Supplementary Note 2).

To summarise the operando study and DFT simulations, a stable [Cu(I)O_2_]^3−^ site has been identified under reductive conditions. The 4*p* orbital energy level relative to that of the 1*s* orbital increases from bulk Cu_2_O, via Cu_2_O clusters to the [Cu(I)O_2_]^3−^ site, which proves the hypothesis that an atomic site has higher unoccupied orbital energy than the clusters and bulk materials (Fig. 1). In addition, a dynamic change of structure between [Cu(I)O_2_]^3−^ and [Cu(II)O_2_(η^2^–O_2_)]^4−^/[Cu(II)O_4_]^6−^ species has been found under CO rich and lean conditions.

## Discussion

In this work, we hypothesise and demonstrate the concept of using electronic structures to control catalytic activity. With the HOMO lower in energy, the atomic Cu site shows a ten times higher activity to that of CuO clusters with only half of the *E*a. An electrophilic [Cu(II)O_2_(η^2^–O_2_)]^4−^ species is obtained upon adsorption of molecular O_2_, which is the key intermediate state between reduced [Cu(I)O_2_]^3−^ site and oxidised [Cu(II)O_4_]^6−^ site. Such dynamics are revealed by a combination of ex situ XPS, in situ Soft X-rays (NEXAFS), hard X-rays (XAS), and EPR techniques supported by DFT simulations. The rarely reported [Cu(II)O_2_(η^2^–O_2_)]^4−^ site has high potential for selective oxidation reactions such as epoxidation and C–H activation. The ability to precisely design and control electronic structures of atomically dispersed active sites will be the key to selective chemical transformations, validated by the modified HOMO of this atomic Cu catalyst. Moreover, we provide a general strategy to use the interaction between atomic metal and supporting cations to achieve this goal, which could be extended to other supported catalysts.

## Methods

### Catalysts preparation

The CuO–CeO_2_ composites were synthesised by the flame spray pyrolysis (FSP) method. The copper and cerium precursor solutions with different Cu:Ce ratios were prepared by mixing appropriate amounts of cerium acetylacetonate (Sigma-Aldrich) with copper 2-ethylhexanoate (Sigma-Aldrich) in a solution of acetic acid (Fluka, >98.5%), methanol (Sigma-Aldrich, 99.9%), and xylene (Sigma-Aldrich, 95%) (25 vol% acetic acid, 25 vol% methanol, and 50 vol% xylene, volume ratio 1:1:2). The resulting total metal concentration was 0.1 mol/L. These precursor solutions were sprayed at 2 mL/min, dispersed with 8 L/min O_2_ (Pangas, 99,95%), and ignited by a premixed CH_4_/O_2_ ring-shaped flamelet (flow rates 1 and 2 L/min, respectively). The resulting flame-made materials were collected from the filter and were not subject to additional temperature treatment. CuO–CeO_2_ materials with CuO contents of 0, 0.05, 0.1, 0.2, 0.4, 0.8, 1, 2, 3, 4, 5, 10, 15 and 20 wt% (based on the weight percentage of CuO in the whole materials) were made by tailoring the ratio between copper 2-ethylhexanoate and cerium acetylacetonate.

### Transmission electron microscopy (TEM) investigations

Samples were prepared by sprinkling a small amount of dry sample powder on 300 mesh copper grids with formvar carbon supported film. TEM images were acquired on JEM 2100 (JEOL, Japan) operated at 200 kV acceleration voltage. The average particle size of CuO–CeO_2_ composites was calculated based on more than 100 particles for each sample.

### HAADF-STEM investigations

Samples were prepared by sprinkling a small amount of dry sample powder on 400 mesh gold grids with lacey carbon support film. High resolution aberration-corrected HAADF-STEM images were either obtained from the probe-corrected (CEOS) JEM ARM 200CF (JEOL, Japan) operated at 200 keV or the probe-corrected (JEOL—COSMO) JEM ARM 300CF (JEOL, Japan) operated at 300 keV. We used a 40 μm probe-forming aperture, resulting in 31.8 mrad probe convergence semi-angle. The HAADF signal was gathered at 2.5 cm STEM camera length, integrating the scattered electron intensity between 100 and 170 mrad. In order to mitigate the accumulation of carbon contamination during STEM imaging, the regions of interest were exposed to an intense electron “beam shower” for 15 min.

### EDS investigations

Data were obtained from the probe-corrected JEM ARM 200CF (JEOL, Japan) with large solid-angle dual EDS detectors for X-ray spectroscopy and elemental mapping. The EDS data acquisition was carried out in STEM imaging mode, with a probe current of 143 pA (probe size is 5 C) at 200 keV acceleration voltage. Each EDS spectrum image is 55 × 55 pixels in size, with 0.05 s exposure time per pixel. To improve the signal-noise ratio, the mapping procedure for each region was performed four times with special drifting correction before the mappings were merged. Gatan Microscopy Suite Software was used for EDS spectrum imaging data acquisition.

### XANES and EXAFS investigations

The analysis of the Cu K-edge (8.979 keV) was performed at 3.0 GeV with a beam current of 300 mA at the Beamline B18 of the Diamond Light Source (UK)^55,56^. A QEXAFS mode was set-up through a fast-scanning Si(111) double crystal monochromator, and Pt-coated branch of collimating and focus mirrors. A couple of Pt-coated harmonic rejection mirrors were inserted between the monochromator and ion chamber to cut off the photons with higher energy. The photon flux at 8 keV (near Cu K-edge at 8.797 keV) was 5 × 10^11^ ph/s with a beam size of 200(H) × 250 (V) μm. The time resolution of the spectra was 2.5 min/spectrum (*k*_max_ = 17, step size 0.3 eV). The XAFS spectra of all samples were measured in an energy range of 8780–10150 keV.

### HERFD-XANES investigations

The measurements were performed at the I20-Scanning beamline at Diamond Light Source (UK)^57,58^. X-ray beam was introduced via Rh coated optic hutch mirrors and Si(111) scanning four bounce monochromators for selecting incident energy^59^. The HERFD-XANES spectra were acquired by scanning the incident energy from 8800.00 to 9400.00 eV with 0.15 eV resolution and monitoring the intensity of the Cu Kβ_1,3_ line (8905.30 eV) by three 100 mm Si (642) spherical crystal analyser. Cu_2_O, CuO, 1 wt% CuO–CeO_2_ and 20 wt% CuO–CeO_2_ were diluted with boron nitride and pressed into a pellet (*d* = 13 mm) for measurement. The XANES analysis was conducted with Demeter software package^60^.

Ex situ XAFS measurements were performed in transmission mode using ion chamber detectors (for samples loading more than 5 wt% CuO) and fluorescence mode using 36-element Ge solid-state detector system (for samples loading less than 5 wt% CuO). Cu foil standard was used for energy shift calibration. CuO, Cu_2_O standards and CuO-CeO_2_ samples (>5 wt% loading) were diluted with cellulose and pressed into a 0.8 mm diameter pellets for transmission measurement. CuO–CeO_2_ samples (<5 wt% loading) were directly pressed into pellets for fluorescence measurement. In order to improve the signal-noise ratio, the spectrum of each sample was measured 3 times for transmission mode and 10–60 times for fluorescence mode.

### Operando XANES and EXAFS investigations

The measurements of CuO–CeO_2_ catalysts were performed in a plug-flow microreactor with the same X-ray beam setup and data acquisition parameters. The catalysts powder was packed into a Kapton foil reaction tube (diameter 6 mm) with quartz wool at both ends. The reaction tube was connected to the gas supply system. A K-type thermal couple was inserted into the catalysts bed to monitor the temperature. A hot air gun was placed under the reaction tube to heat the catalysts bed (heating and cooling ramp rate of 10 °C min^−1^ and 20 °C min^−1^, respectively). The heating zone was sheathed with an additional ceramic drivepipe to improve the heat conductivity and prevent uneven heating. Two 3 mm × 15 mm windows were placed on both sides of the ceramic drivepipe to let X-rays passing through.

Totally, 1, 5 and 20 wt% CuO–CeO_2_ sample powders were measured in fluorescence mode. Boron nitride was used to dilute the 20 wt% sample to minimise the self-absorption effect. During the reaction, XAFS spectra were acquired every 150 s continuously.

CO oxidation reaction was carried out under 1–5 vol% CO/He and 1–5 vol% O_2_/He over approximately 10 mg of the catalysts (WHSV = 6 × 10^5^ mL_CO_ g_CuO_^−1^ h^−1^). Outlet gases were sampled continuously with the Quadrupole Mass Spectrometer Quantitative Gas Analyser (Hiden Analytical, UK). The Hiden QGA can continuously sample and scan atomic mass range from 1 to 200 AMU with 500 times/s for measurement speed. The analysis sensitivity is 100% to 100 PPB subject to spectral interference. The gas profile was simulated and analysed by automatic subtraction of spectral overlaps.

XAFS data were analysed by Demeter software package (including Athena and Artemis, version 0.9.25)^60^. Athena software was used for data extraction and XANES analysis. Artemis software was used to fit the *k*^3^-weighted EXAFS data (3.0 Å^−1^ < *k* < 12.5 Å^−1^) with 1.0 Å < *R* < 4.0 Å (0.05–5 wt%) or 1.0 Å < *R* < 3.0 Å (5–20 wt%). The calculated amplitude reduction factor S_0_^2^ from EXAFS analysis of Cu foil was 0.809, which was used as a fixed parameter for EXAFS fitting. The increase of the Debye–Waller factor *σ*^2^, indicating the relative displacement of absorber and backscatter atoms, was calculated based on a linear fitting of *σ*^2^ value as a function of temperature. The same changing rate (slope) of *σ*^2^ value was applied to the spectra collected during the CO oxidation experiment while the initial *σ*^2^ value was determined by the fitting of the spectra collected at room temperature.

### X-band EPR investigations

The experiments were performed in continuous-wave (cw) mode on a Bruker E580 X-band EPR spectrometer equipped with a Bruker ER4122-SHQE cavity. Totally, 20 mg powder of each sample was loaded into a high purity quartz EPR tube (4.0 mm o.d., 3.0 mm i.d.) for measurement. All the cw EPR spectra were acquired at room temperature over a wide magnetic field range. Typical spectrometer parameters were: sweep time (300 s), centre field (300 mT), sweep width (300 mT), modulation frequency (100 kHz), microwave frequency (9.87 GHz), microwave power (2.0 mW). Operando EPR spectra were recorded using a fixed-bed continues-flow reactor setup^53^ inserted in the EPR cavity with ~80 mg of the powder catalyst exposed to the flow of reactive/inert gases with WHSV of 60,000 mL h^−1^ g^−^^1^ at 393 K. Typical spectrometer parameters were: sweep time (160 s), centre field (270 mT), sweep width (320 mT), modulation frequency (100 kHz), microwave frequency (9.32 GHz) and microwave power (20.0 mW). The EPR spectra were simulated and analysed using the Easyspin^61^ toolbox running in MATLAB.

### XRD investigations

The measurement was performed on Bruker D8 diffractometer with a voltage of 40 kV at 30 mA, using a Cu source with *K*_α1_ = 1.540562 Å and *K*_α2_ = 1.544398 Å. The contributions of *K*_α2_ line in the XRD patterns were subtracted.

### SXPD investigations

The measurement of CuO–CeO_2_ catalysts was carried out at Beamline I11 in Diamond Light Source (UK)^62^. A monochromatic beam with calibrated wavelength at 0.826115(10) Å from Si (SRM640c) standard was used to obtain X-ray diffraction patterns (from 2*θ* = 2–92° with 0.004° step size). The powder patterns were obtained using the fast position sensitive detector^63^.

### Nitrogen adsorption investigations

The adsorption-desorption isotherms were recorded at 77 K using a Micromeritics 3Flex surface characterisation analyser. The samples were degassed in vacuum at 200 °C overnight for removal of any adsorbates. Specific surface areas were determined according to the BET model.

### XPS investigations

The measurement was performed on a Thermo Fisher Scientific NEXSA spectrometer. The samples were analysed using a micro-focused monochromatic Al X-ray source (72 W) over an area of approximately 400 microns. Data were recorded at the pass energies of 200 eV for survey scans (1.0 eV step) and 50 eV for the high-resolution scans (0.1 eV step). Charge neutralisation of the sample was achieved using a combination of both low energy electrons and argon ions. C 1*s* electron at 284.8 eV was used as the standard reference to calibrate the photoelectron energy shift. XPS spectra in Ce 3*d* region from 840 to 940 eV was collected and fitted to identify the ratio of Ce in different oxidation states. Data analysis were performed on the CasaXPS software (version: 2.3.18PR1.0).

### Catalytic tests

The catalytic performance in CO oxidation was evaluated on a FD-2000 fix-bed reactor (Huasi, China). Approximately, 10–100 mg catalyst powder was packed into a quartz tube. A mixture of gases (1 vol% CO, 10 vol% O_2_, 89 vol% N_2_) was introduced to the reactor via 4 MFCs. The exhaust gas was analysed by AO2000 Series Advance Optima Continuous Gas Analyser (ABB, Germany) equipped with three individual sensors: IR spectrometer for CO and CO_2_, superparamagnetic O_2_ analyser and thermal conductivity detector for H_2_.

### CO-TPR and CO-TPD investigations

The measurements were performed on the same FD-2000 reactor. Typically, 100 mg CuO–CeO_2_ was put into a quartz tube. The content of CO and CO_2_ in the exhaust gas was quantified by the same AO2000 analyser. To remove any potential carbon contamination, the sample was pre-oxidised in 100 mL/min 5% O_2_/He at 673 K for 30 mins. After oxidation, the sample was cooling down to room temperature in He. For CO-TPR, the sample was kept in 2% CO/N_2_ flow (100 mL/min) and heated up to 473 K with a rate at 5 K/min. For CO-TPD, the sample adsorbed CO in 2% CO/N_2_ (100 mL/min) at room temperature for 30 min and was blown with He (100 mL/min) for another 30 min to remove physically adsorbed CO. The CO adsorbed sample was then heated up to 673 K in He with a rate at 5 K/min.

### NAP-NEXAFS investigations

In situ NAP-NEXAFS experiments were accomplished at the ISISS beamline of BESSY II in Berlin (Germany). The X-ray is sourced from a bending magnet (D41) and a plane grating monochromator (PGM) with an energy range from 80 to 2000 eV (soft X-ray range) and flux of 6 × 10^10^ photons/s with 0.1 A ring current using a 111 µm slit and an 80 µm × 200 µm beam spot size. The in situ measurements were accomplished in the ambient pressure X-ray end-station using a Faraday-cup to collect the NEXAFS spectra in the O K-edge and Cu L-edge at different partial pressures. The reaction products were online monitored using an electron impact mass spectrometer (“PRISMA”, PFEIFFER VACUUM GmbH, Asslar (Germany)) connected directly to the main experimental chamber by a leak valve. The pressure in the specimen chamber was precisely controlled (UHV or 0.1–1 mbar) by simultaneous operations of several mass flow controllers for reactive gases and a PID-controlled throttle valve for pumping gas out. Sample pellets (8 mm diameter) was heated uniformly from the back side by a focused infra-red laser. A stainless-steel plate was placed behind the pellet to improve the heat transfer. The temperature was monitored by a K-type thermocouple and regulated by a PID controller connected to the laser power source.

NEXAFS spectra at Cu L_3_/L_2_ edge (920–960 eV), Ce M_5_/M_4_ edge (860–920 eV) and O K-edge (520–560 eV) were measured in either total electron yield (TEY) mode or Auger electron yield (AEY) mode. AEY mode, which has a worse signal to noise ratio compared with TEY mode, was only used for the O K-edge measurement to avoid gas phase absorption signal while gas-phase O_2_ or CO was present. The excitation energy scale was calibrated for the Cu L-edge using the absorption edge of metallic Cu (932.67 eV at the adsorption edge inflexion point). In the case of the O K-edge, it was calibrated using the π* transition of gas-phase O_2_ (absorption peak at 530.8 eV).

### Computational methods

Spin-polarised DFT + *U* calculations were carried out with the generalised gradient approximation with the Perdew–Burke–Ernzerhof (GGA-PBE) functional using the Vienna Ab initio Simulation Package (VASP)^64,65^. The projector augmented wave (PAW) pseudopotentials were employed to describe the electron–core interaction^66,67^. The wave functions were expanded in plane waves with a kinetic energy cut-off of 400 eV for all calculations. The *U* value for the Coulomb interaction correction was set to 5.0 eV to describe the electronic property of Ce appropriately. The calculated lattice parameters of bulk ceria using the DFT + *U* method (5.448 Å) is in good agreement with the experimental value (5.411 Å). The CeO_2_(111) surface was modelled using a nine-atomic-layer slab with the *p*(3 × 3) supercell. The bottom three layers were fixed at the bulk parameters, while the upper six layers were allowed to fully relax. The CeO_2_(110) surface was modelled using a nine-atomic-layer slab with the *p*(2 × 2) supercell. The bottom three layers were fixed at the bulk parameters, while the upper six layers were allowed to fully relax. The CeO_2_(100) surface was modelled using an eight-atomic-layer slab with the *p*(2 × 2) supercell. The bottom two layers were fixed at the bulk parameters, while the upper six layers were allowed to fully relax. The CuO(111) and Cu_2_O(111) surfaces were constructed using *p*(2 × 2) supercell with four layers, in which the bottom two layers were fixed and the upper two layers were fully relaxed. A vacuum region of 15 Å was used for all surface models in order to remove the interactions between the periodic images along the *c*-axes. The CeO_2_(111) surface is selected as the representative support for the Cu single-sites. To simulate the Cu(I/II) single-sites, two models are constructed based on their structures in the forms of [Cu(I)O_2_]^3−^ and [Cu(II)O_4_]^6−^, which are identified by in situ XAFS (Fig. 5). In the model for [Cu(I)O_2_]^3−^ site, an initial neutral Cu atom is put on CeO_2_(111) surface and coordinates with two lattice oxygen atoms in CeO_2_. As revealed by the NAP-NEXAFS, the [Cu(I)O_2_]^3−^ site can adsorb one O_2_ molecule and evolve into the [Cu(II)O_4_]^6−^ site (Fig. 4). In the model for [Cu(II)O_4_]^6−^ site, there are four Cu–O bonds in total to stabilise the structure and maintain the +2 oxidation states. The two O atoms provided by CeO_2_ support are inherited from the [Cu(I)O_2_]^3−^ structure (adding Cu atom on CeO_2_ surface) whereas the other two Cu–O bonds are introduced by the adsorption of molecular O_2_ which is available in the reaction gas. There are four Ce–O–Cu bridges. The calculation shows the formation of additional two Ce–O bonds in [Cu(II)O_4_]^6−^ structure origins from the cleavage of the O–O bond in [Cu(II)O_2_(η^2^–O_2_)]^4−^ (Supplementary Fig. 27). As a result, the newly formed Cu–O–Ce bridges give similar signals to that of lattice oxygen species in the O K edge NAP-NEXAFS (Fig. 4d, right). All calculations were converged until the force on each atom was less than 0.02 eV Å^−1^. The Brillouin zone integration was performed using a 2 × 2 × 1 Monkhorst–Pack (MP) k-points for surface structure optimisations and transition state (TS) calculations, while a 12 × 12 × 1 MP k-points for the Bader charge analysis and charge density difference calculations. The adsorption energy (*E*_ads_) was defined as *E*_ads_ = *E*_total_ − *E*_adsorbate_ − *E*_slab_, with *E*_adsorbate_, *E*_slab_ and *E*_total_ denoting the total energy of the adsorbate, the surface, and the complex of surface and adsorbate respectively. The TS was located by the climbing image nudged elastic band method^68,69^. The effective charges (*Q*_X_) were calculated by Bader’s charge population analysis with the equation: *Q*_X_ = *Z*_X_ − *q*_Bader,X_, with *Z*_X_ and *q*_Bader,X_ denoting the number of valence electrons and the calculated Bader charge of X atom, respectively.

## Supplementary information

Supplementary Information

Peer Review File

## Data Availability

More experimental details and additional data can be found in the Supplementary Information (Supplementary Figs. 1–29, Supplementary Tables 1–13 and Supplementary Notes 1 and 2). The data that support the findings of this study are available from the corresponding authors B.W. (email: bolun.wang@ucl.ac.uk) or F.R.W. (email: ryan.wang@ucl.ac.uk) upon reasonable request.
