# Supplementary material for: Adsorption and activation of molecular oxygen over atomic copper(I/II) site on ceria
Source: Nat Commun. 2020 Aug 11;11:4008. doi: 10.1038/s41467-020-17852-8 (PMC7419315; doi:10.1038/s41467-020-17852-8)
Supplement: Supplementary file 1 — Supplementary Information [file 41467_2020_17852_MOESM1_ESM.pdf]

Supplementary Information

Adsorption and activation of molecular oxygen over atomic copper(I/II) site on ceria

Kang et al.

Supplementary Figures

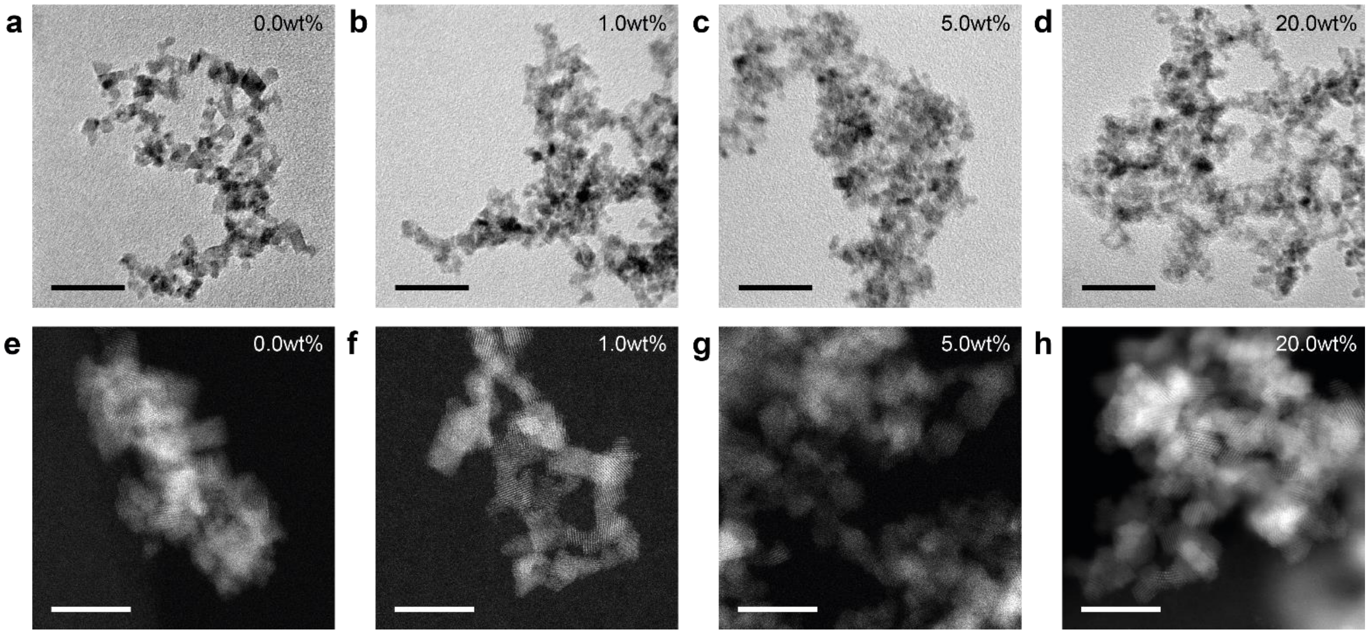

Supplementary Figure 1. Transmission Electron Microscopy (TEM) and High Resolution Aberration-Corrected High Angle Annular Dark Field-Scanning Transmission Electron Microscopy (HAADF-STEM) images of CuO-CeO_2 catalysts. TEM images of **a**, 0wt%, **b**, 1wt%, **c**, 5wt% and **d**, 20wt% CuO-CeO_2. HAADF-STEM images of **e**, 0wt%, **f**, 1wt%, **g**, 5wt% and **h**, 20wt% CuO-CeO_2. Scale bars: 25 nm in **a-d** and 10 nm in **e-h**.

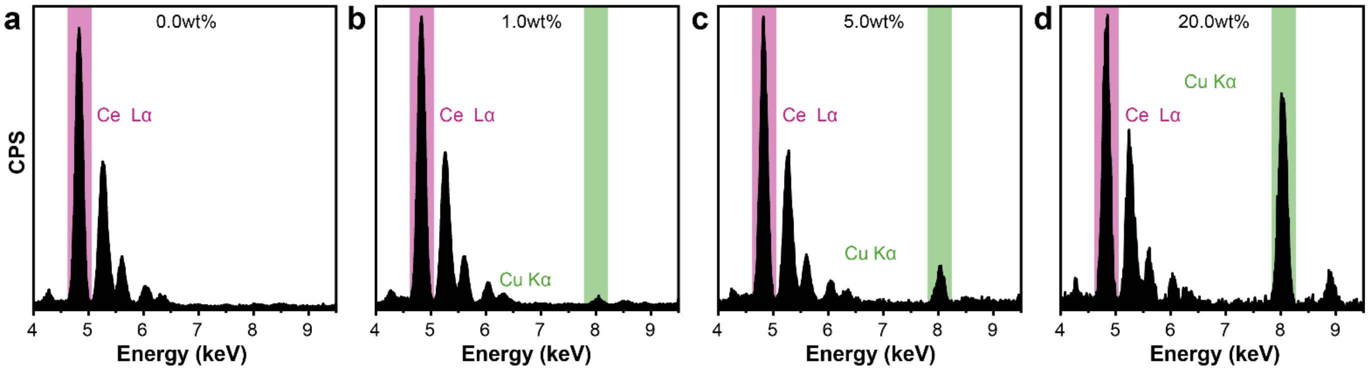

Supplementary Figure 2. Energy-Dispersive X-Ray Spectroscopy (EDS) of CuO-CeO_2 catalysts. EDS of **a**, 0wt%, **b**, 1wt%, **c**, 5wt% and **d**, 20wt% CuO-CeO_2, collected from same areas in Supplementary Figure 1e-h. Peaks in the pink region and green region correspond to Ce Lα and Cu Kα X-ray emission.

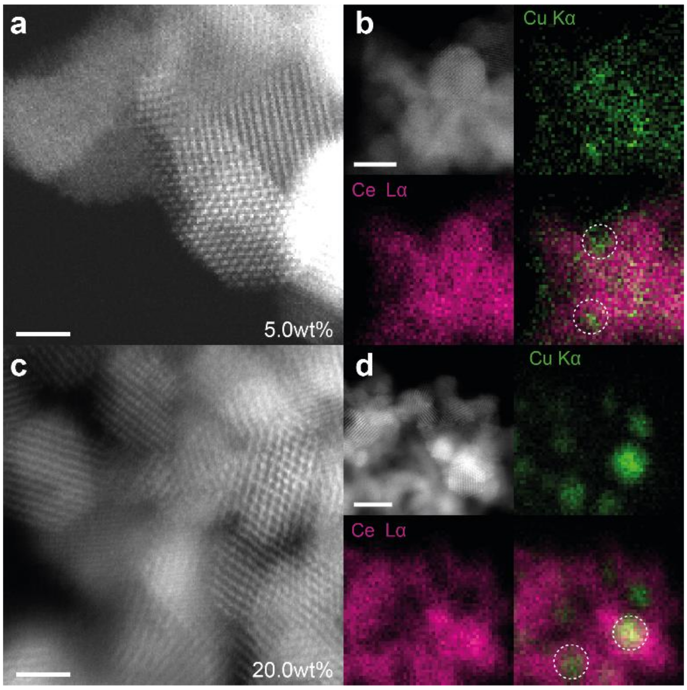

Supplementary Figure 3. HAADF-STEM image and EDS mapping for CuO-CeO2. a,**b**, HAADF-STEM images of CuO-CeO_2 at 5wt% and 20wt% CuO loading. **c**,**d**, Corresponded EDS mapping. Red: Ce; Green: Cu. The white circles suggest the CuO cluster/nanoparticle domains. Scale bars: 2 nm in **a**,**c** and 5 nm in **b**,**d**.

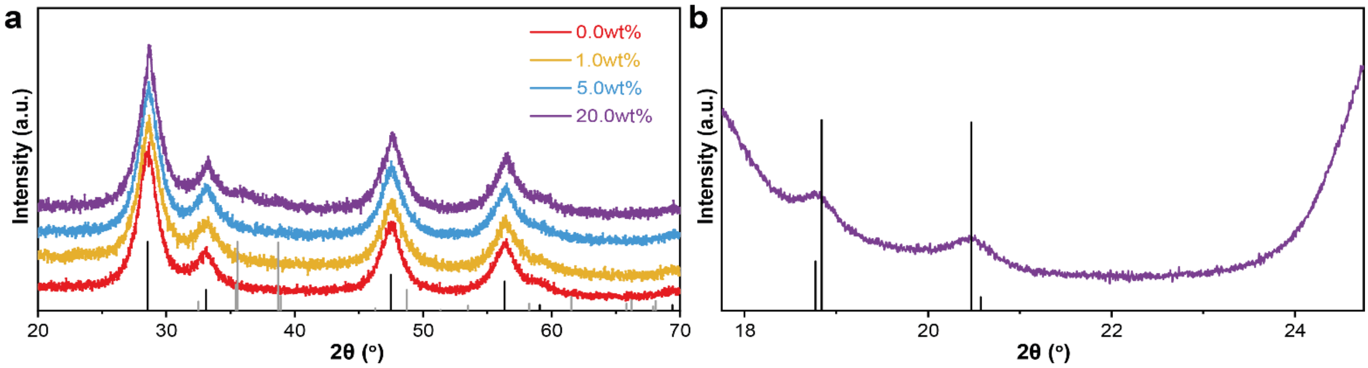

Supplementary Figure 4. X-ray diffraction (XRD) and Synchrotron X-ray Powder Diffraction (SXPD) patterns of CuO-CeO2. a, XRD patterns of CuO-CeO_2 catalysts with 0wt% (red curve), 1wt% (orange curve), 5wt% (blue curve) and 20wt% (purple curve) loading concentration. The standard reference pattern of cubic ceria (ICDD PDF 00-034-0394: black bars) and monoclinic CuO (ICDD PDF 00-048-1548: grey bars) are shown as well. **b**, SXPD data of 20wt% CuO-CeO_2 obtained at beamline I11 (λ=0.826115(10) Å). The diffraction peak positions for monoclinic CuO are marked at 18.78°(002), 18.84°(11$\bar{1}$), 20.47°(111) and 20.57°(200).

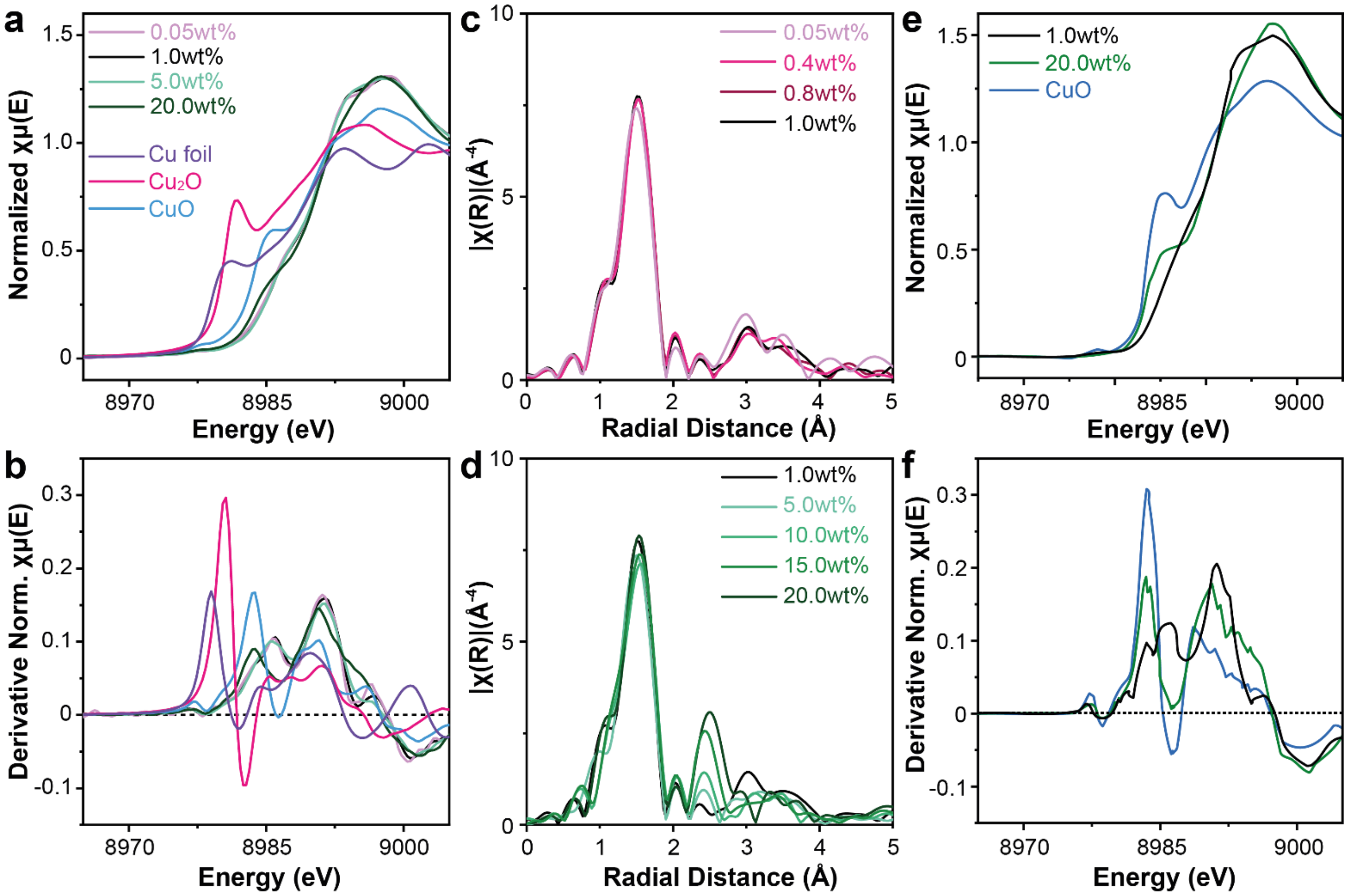

Supplementary Figure 5. *Ex situ* X-ray Absorption Near Edge Structure (XANES) and Extended X-ray Absorption Fine Structure (EXAFS) of CuO-CeO_2 catalysts. a, XANES spectra of 0.05wt%, 1wt%, 5wt%, 20wt% CuO-CeO_2, and Cu foil, Cu_2O, CuO standards. **b**, First derivatives of the XANES spectra. The peak positions of 1*s* → 3*d* and 1*s* → 4*p* transition are listed in Supplementary Table 2. **c**, EXAFS spectra of 0.05wt%, 0.4wt%, 0.8wt% and 1wt% CuO-CeO_2. **d**, EXAFS spectra of 1wt%, 5wt%, 10wt%, 15wt% and 20wt% CuO-CeO_2. **e**, High Energy Resolution Fluorescence Detected X-ray Absorption Near Edge Spectroscopy (HERFD-XANES) spectra of 1wt%, 20wt% CuO-CeO_2 and CuO standard. **f**, First derivatives of the XANES spectra.

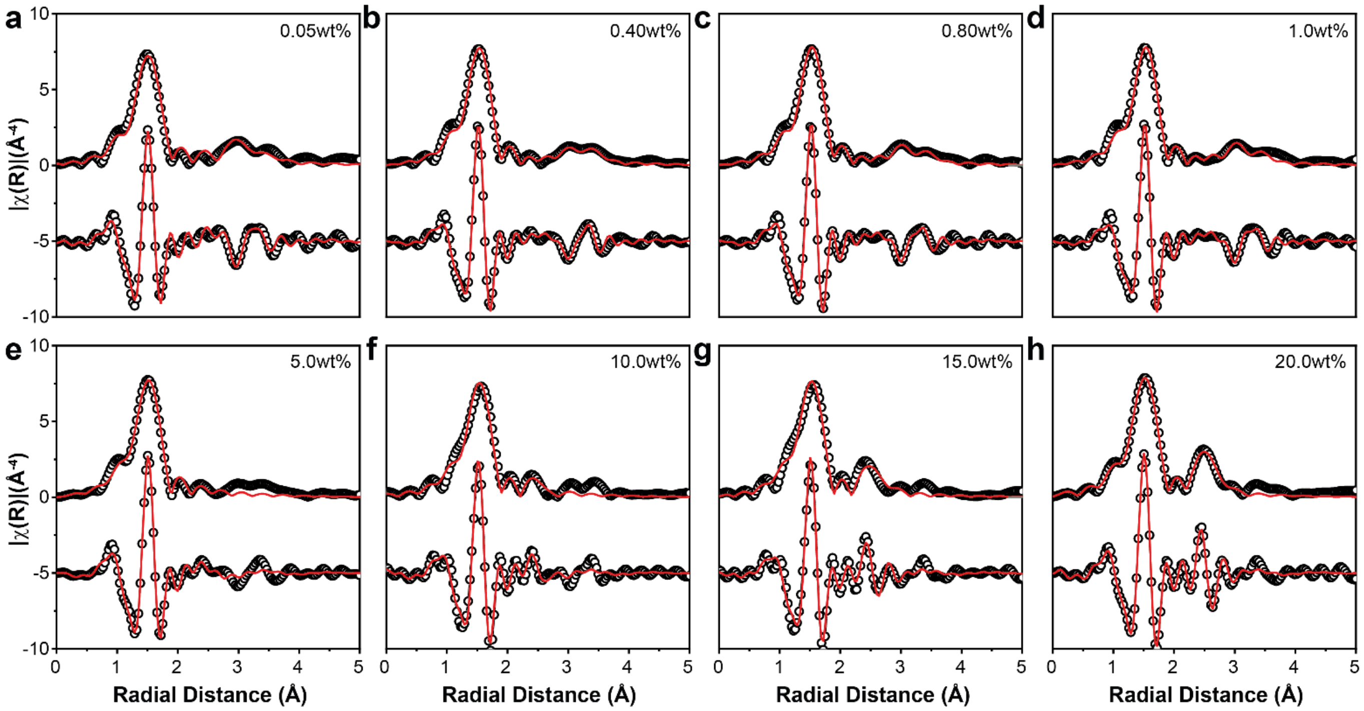

Supplementary Figure 6. EXAFS fitting results of CuO-CeO2 catalysts at different loadings. The k^3-weighted Fourier Transform EXAFS data (without phase correction) are shown together with fitting results (red curves).

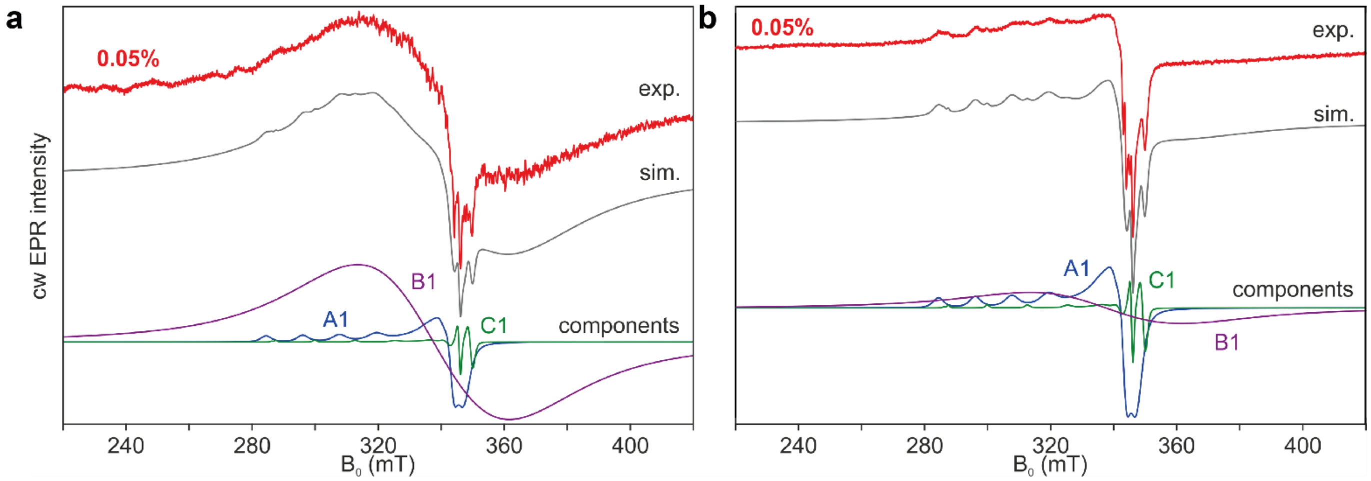

Supplementary Figure 7. The X-band cw Electron Paramagnetic Resonance (EPR) spectra of 0.05wt% CuO-CeO2. a, before and **b**, after catalysis (red lines) recorded at 298 K, together with the simulated composite spectra (grey lines) and calculated spectra of the individual components (red, green, blue lines). Spin Hamiltonian parameters are summarised in Supplementary Table 3.

Supplementary Note 1. Signals of the well-defined Cu(II) oxo dimers with 3.6 Å inter-copper distances become evident in the spectra above 1wt% (Fig. 2g, $d_\perp$ lines at 310, 380 mT, signal K in

reported literature and our previous study[1, 2]). At 5wt% and 20wt%, the paramagnetic Cu(II) content is equivalent to that of 0.4wt% and 0.05wt% CuO-CeO_2. At higher Cu(II) loadings, the probability of Cu(II)-Cu(II) interactions increases. Considering that the Cu(II) ions in crystalline CuO couple anti-ferromagnetically,[3] the microcrystalline CuO domains or well-arranged clusters are EPR silent. Therefore, we conclude that atomic Cu(II) site is the dominant species below 1wt% and CuO clusters are the dominant species above 5wt%.

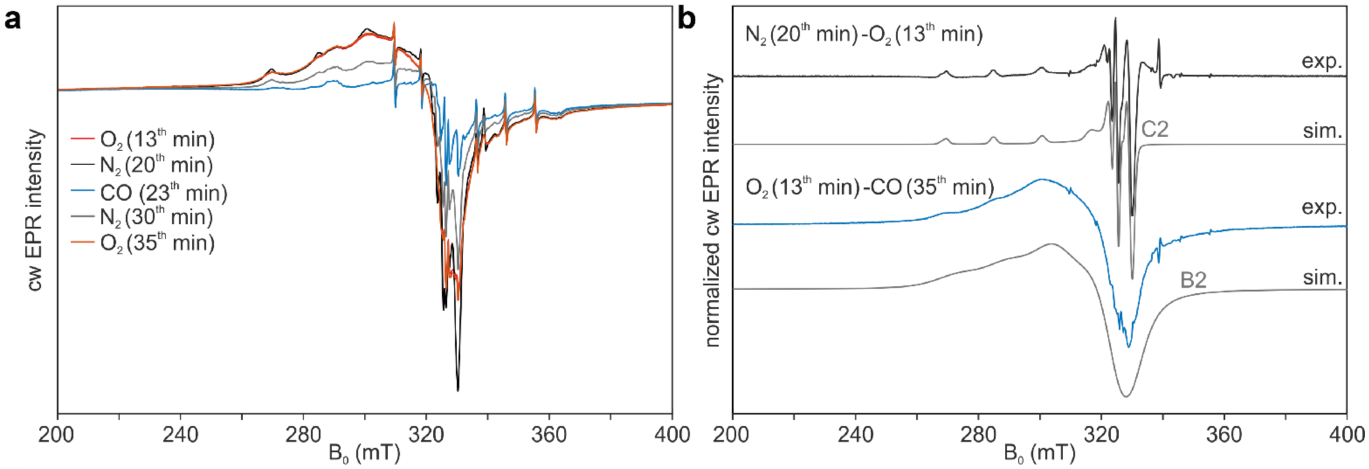

Supplementary Figure 8. Changes in the EPR spectra under O_2/N_2/CO. a, X-band cw EPR spectra of 1wt% CuO−CeO_2 recorded at 393 K during the treatment with O_2/N_2/CO/N_2/O_2. **b**, The plot of the normalised difference spectra and simulations for N_2-O_2 signals and O_2-CO signals.

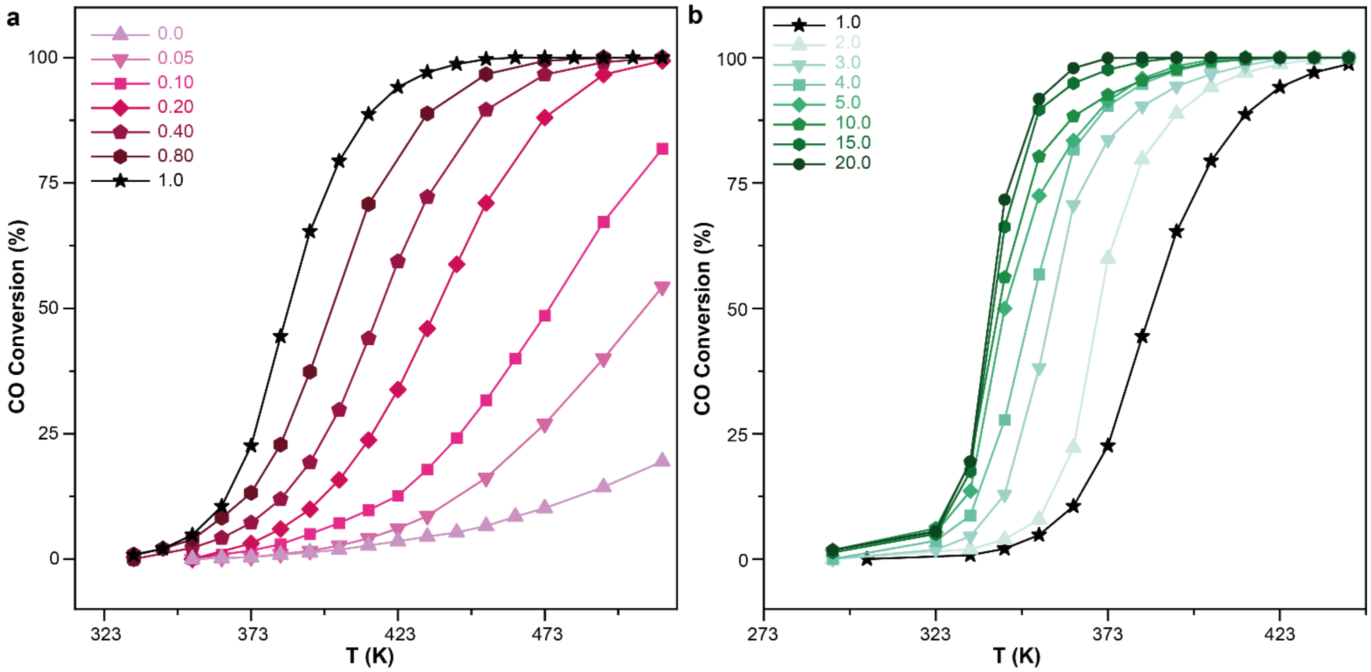

Supplementary Figure 9. Conversion of CO as a function of temperature and CuO loading (0.0wt% to 20.0wt% CuO). Weight hourly space velocity (WHSV) per gram of catalysts: 1,500 $mL_{CO} \cdot h^{-1} \cdot g^{-1}$, 1% CO + 10% O_2.

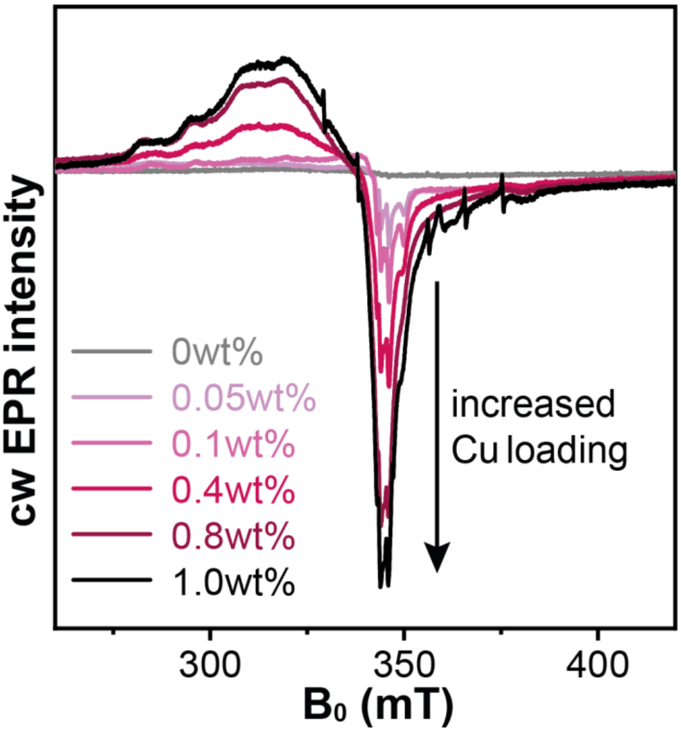

Supplementary Figure 10. X-band cw EPR spectra for CuO-CeO_2 with CuO loading from 0wt% to 1wt% after catalysis.

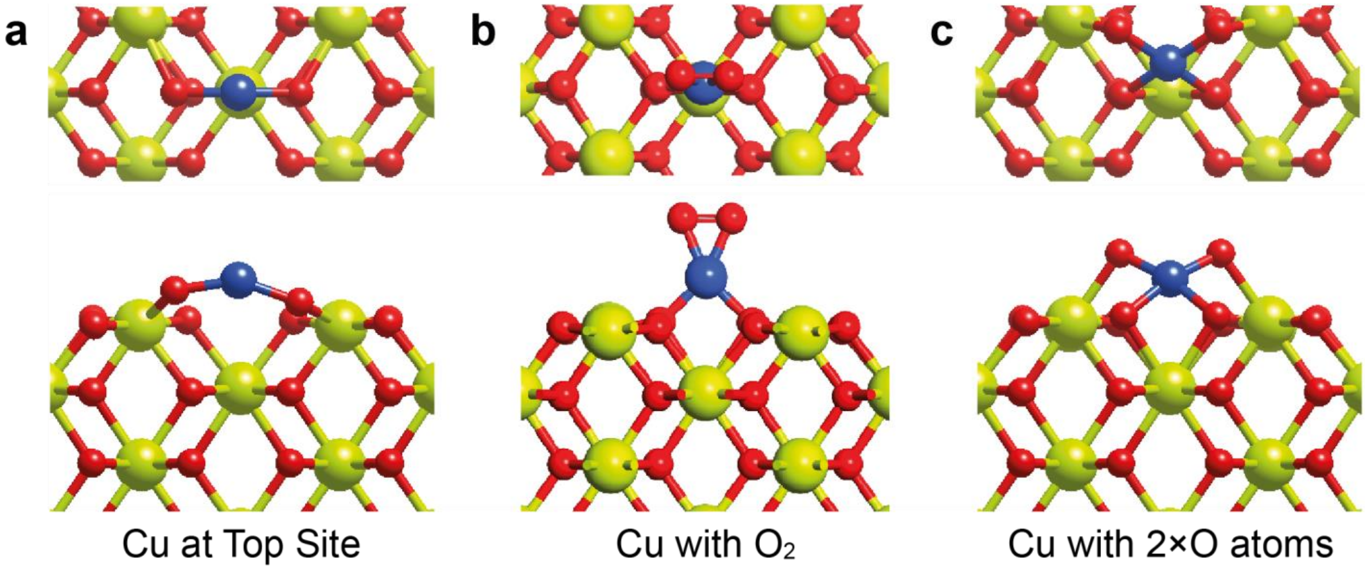

Supplementary Figure 11. Optimised structures of atomic Cu sites on CeO_2(110) surface based on DFT+*U* simulations. **a**, $[Cu(I)O_2]^{3-}$ site. **b**, Adsorption of O_2 forms $[Cu(II)O_2(\eta^2\text{-}O_2)]^{4-}$ site. **c**, Dissociated $[Cu(II)O_4]^{6-}$.

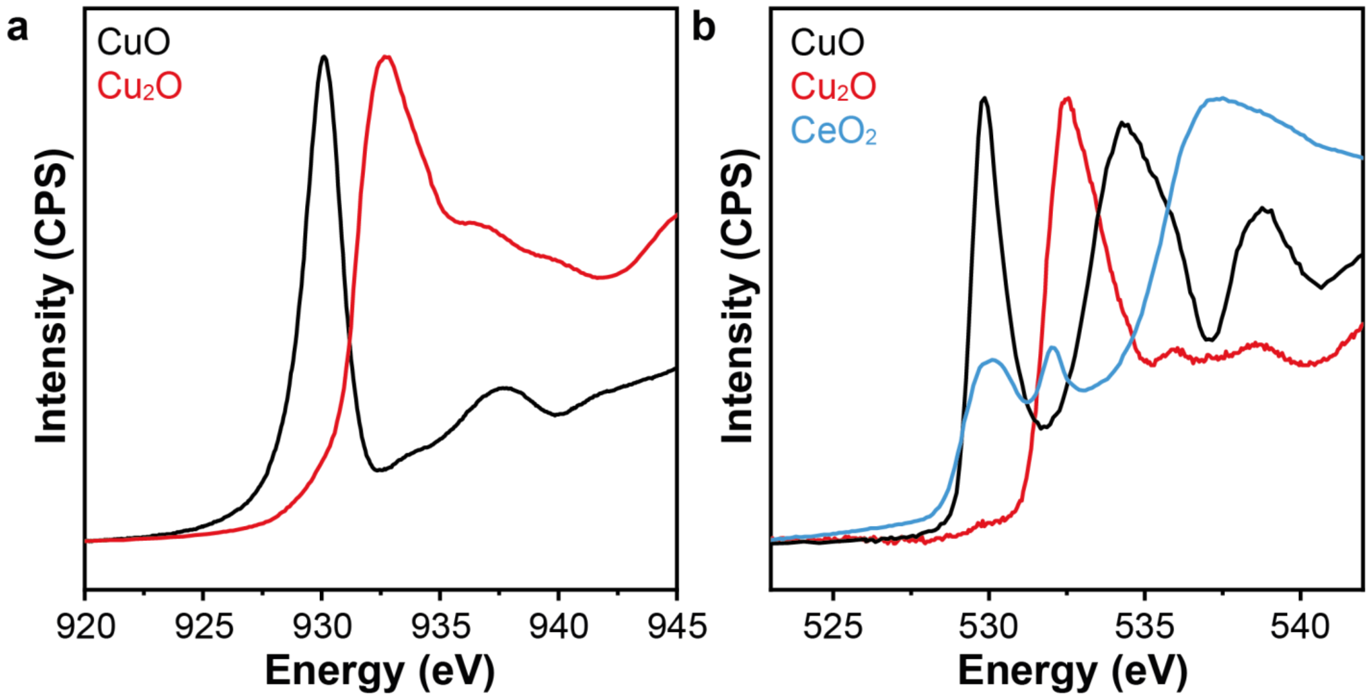

Supplementary Figure 12. Cu L_3-edge and O K-edge NEXAFS spectra of CuO, Cu_2O and CeO_2 standards.

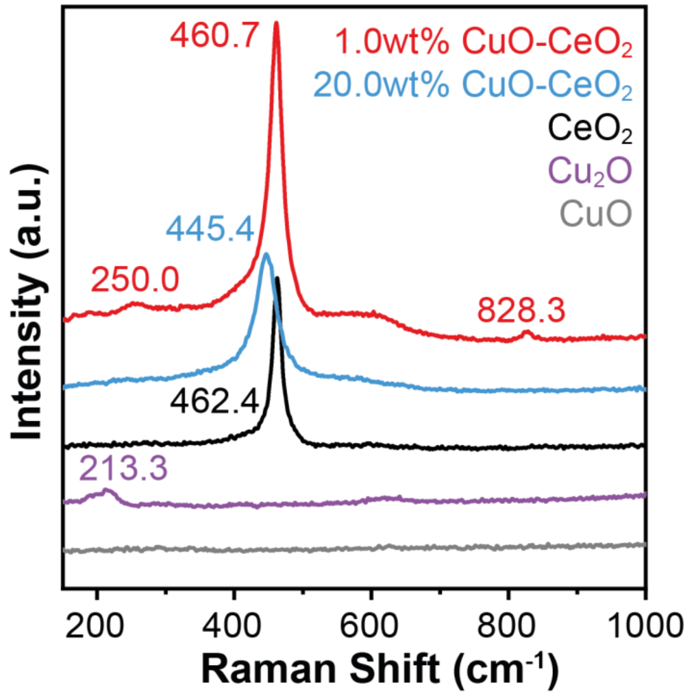

Supplementary Figure 13. Raman spectra of CuO-CeO_2 and CeO_2, Cu_2O, CuO standards using 515 nm laser.

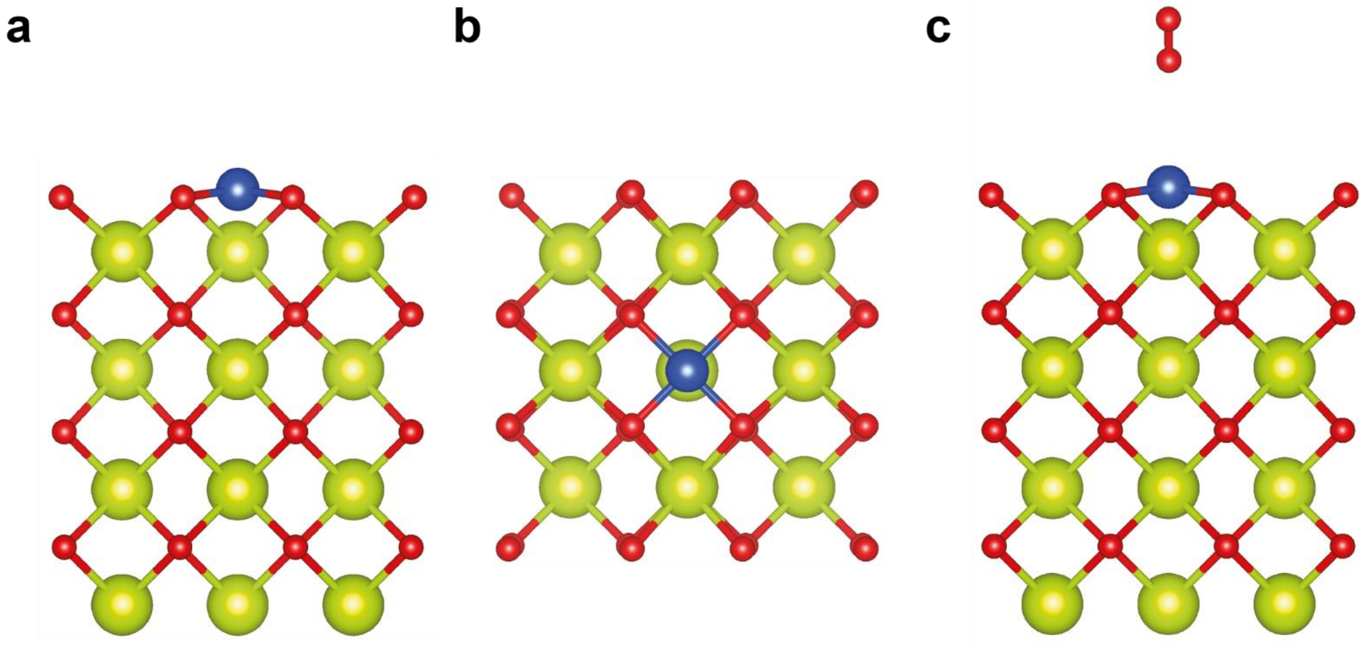

Supplementary Figure 14. Optimised structures of atomic Cu sites on CeO_2(100) surface based on DFT+*U* simulations. a, Side view of a 4-fold coordinated Cu site. **b**, Top view of a 4-fold coordinated Cu site. **c**, Side view of a physically adsorbed O_2 molecule on a 4-fold coordinated Cu site.

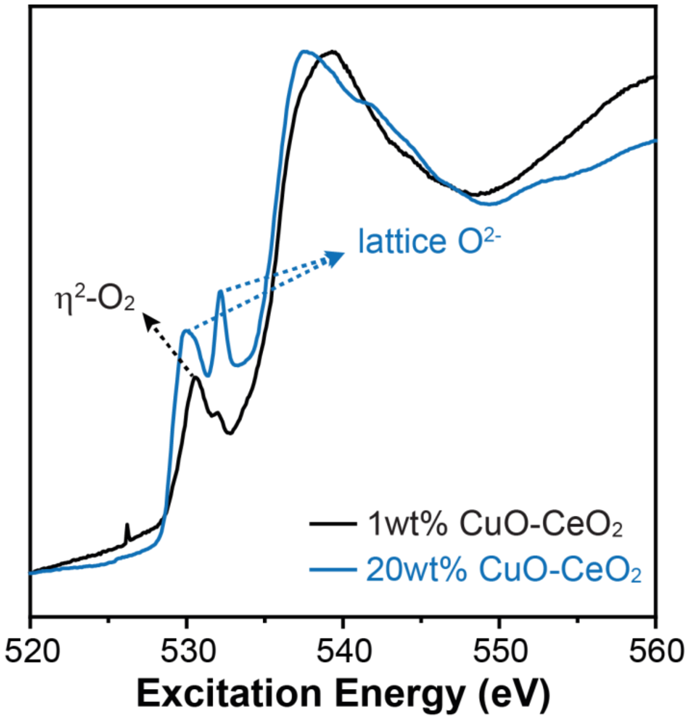

Supplementary Figure 15. The O K-edge NEXAFS of different surface oxygen species on 1wt% CuO-CeO_2 and 20wt% CuO-CeO_2 under UHV at 298 K.

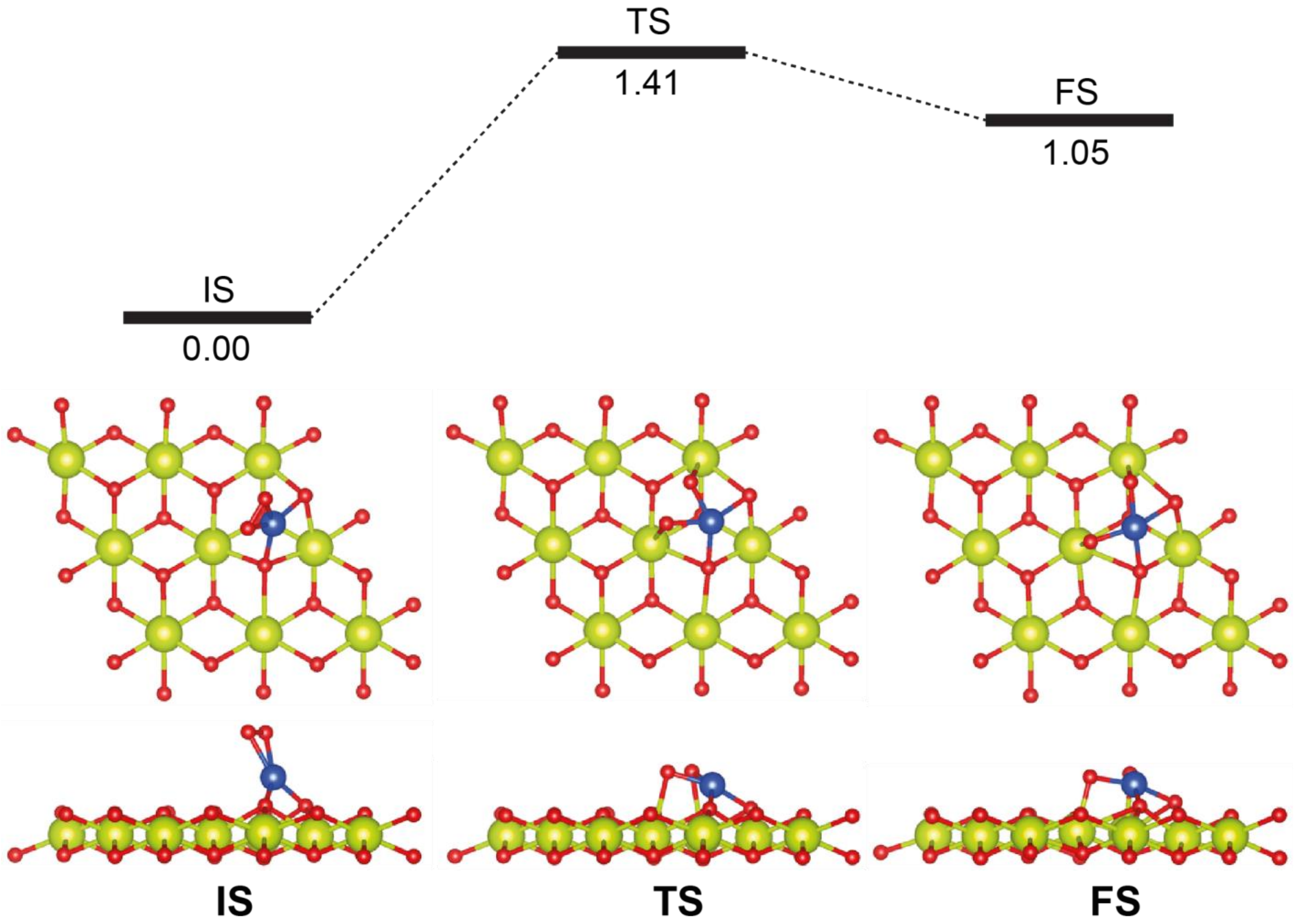

Supplementary Figure 16. Reaction pathway for the decomposition of adsorbed O_2 molecule on CeO_2(111) surface supported Cu single site. Initial state (IS): adsorbed O_2 molecule; transition state (TS): decomposing state of O_2; final state (FS): double adsorbed O atoms. Colour schemes: yellow, Ce; blue, Cu; red, O.

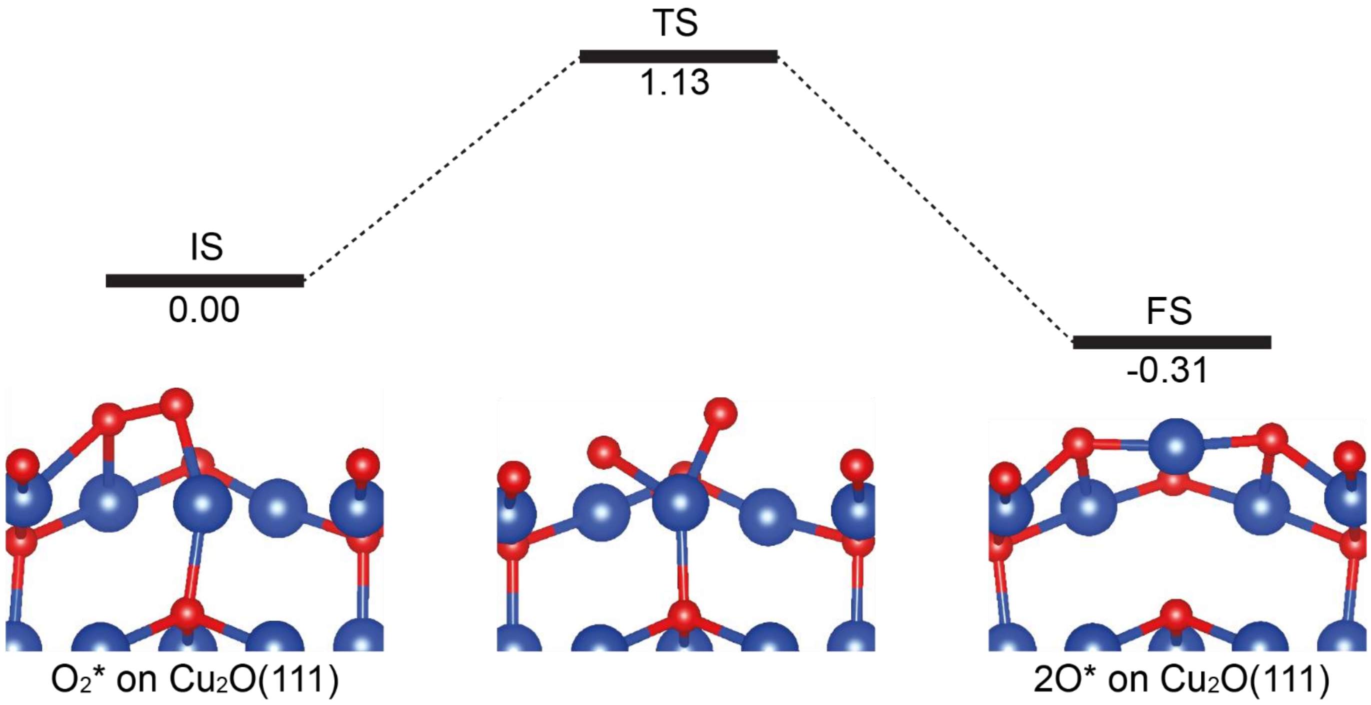

Supplementary Figure 17. Reaction pathway for the decomposition of adsorbed O_2 molecule on $Cu_2O(111)$. Initial state (IS): adsorbed O_2 molecule on $Cu_2O(111)$; transition state (TS): decomposing state of O_2; final state (FS): double adsorbed O atoms on $Cu_2O(111)$. Colour schemes: blue, Cu; red, O.

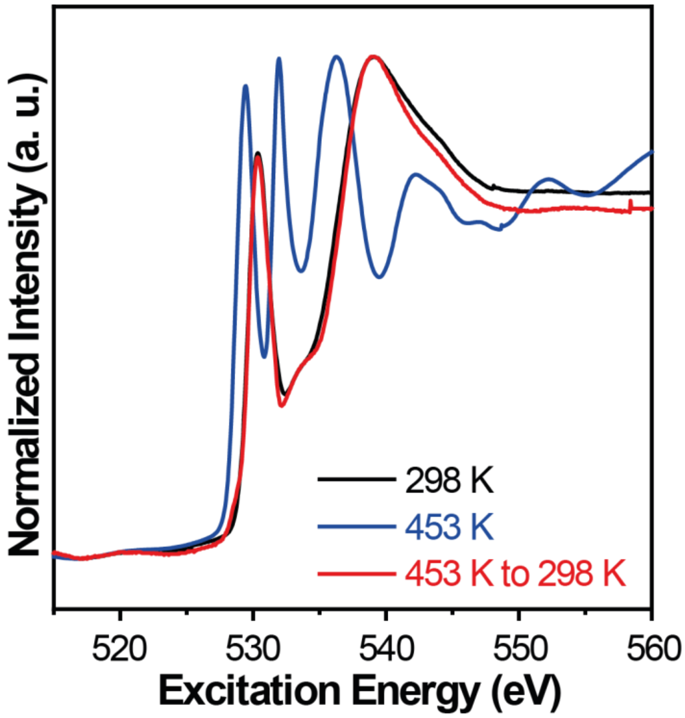

Supplementary Figure 18. The NEXAFS of 1wt% $CuO-CeO_2$ on O K-edge at UHV. It shows the reversible conversion between $[Cu(II)O_2(\eta^2-O_2)]^{4-}$ (298 K) and $[Cu(II)O_4]^{6-}$ (453 K).

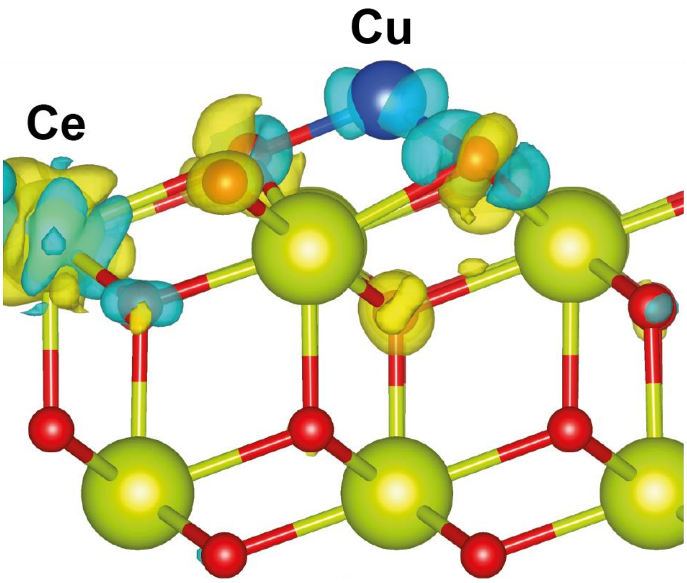

Supplementary Figure 19. The Charge density difference (CDD) plot of atomic Cu(I) site on CeO_2(111). In the CDD plot, yellow isosurfaces denote electron gain and blue isosurfaces mean the electron lost.

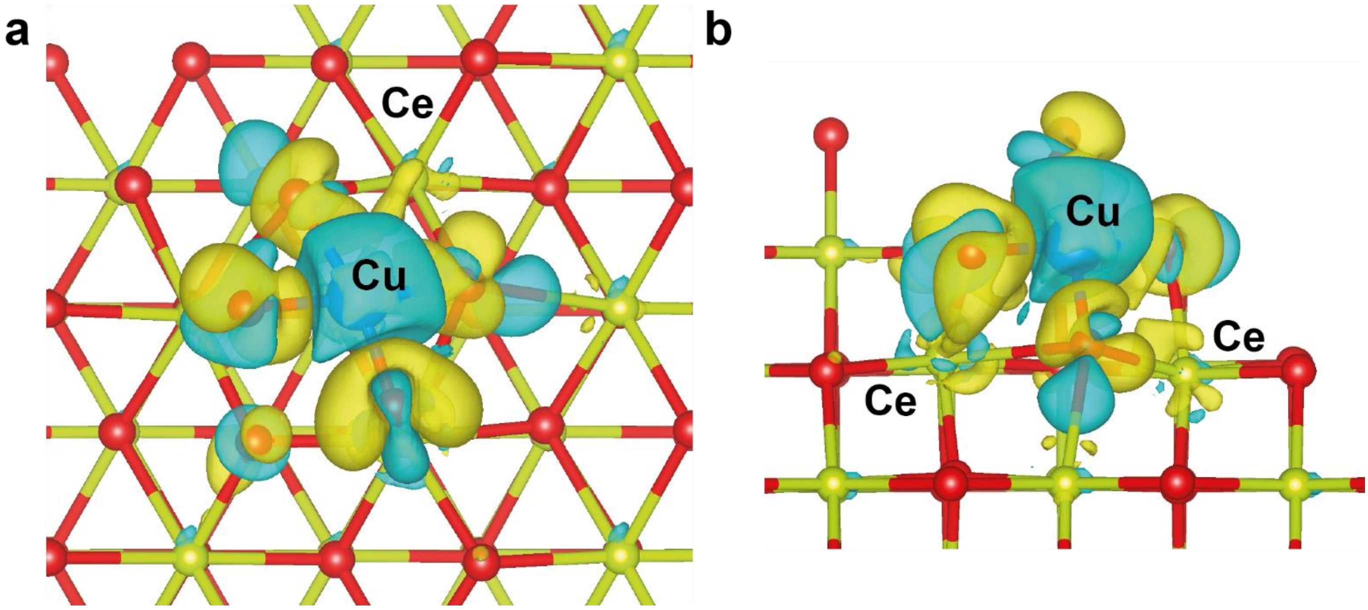

Supplementary Figure 20. The CDD plot of atomic Cu(II) on CeO_2(111) with adsorbed O atoms. In the CDD plots, yellow isosurfaces denote electron gain and blue isosurfaces mean the electron lost.

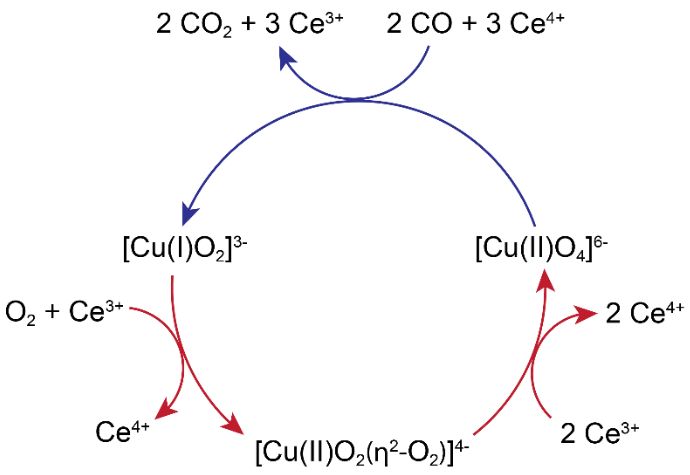

Supplementary Figure 21. Evolution of the atomic Cu site and Ce(III/IV) redox pair during O_2 activation and CO oxidation.

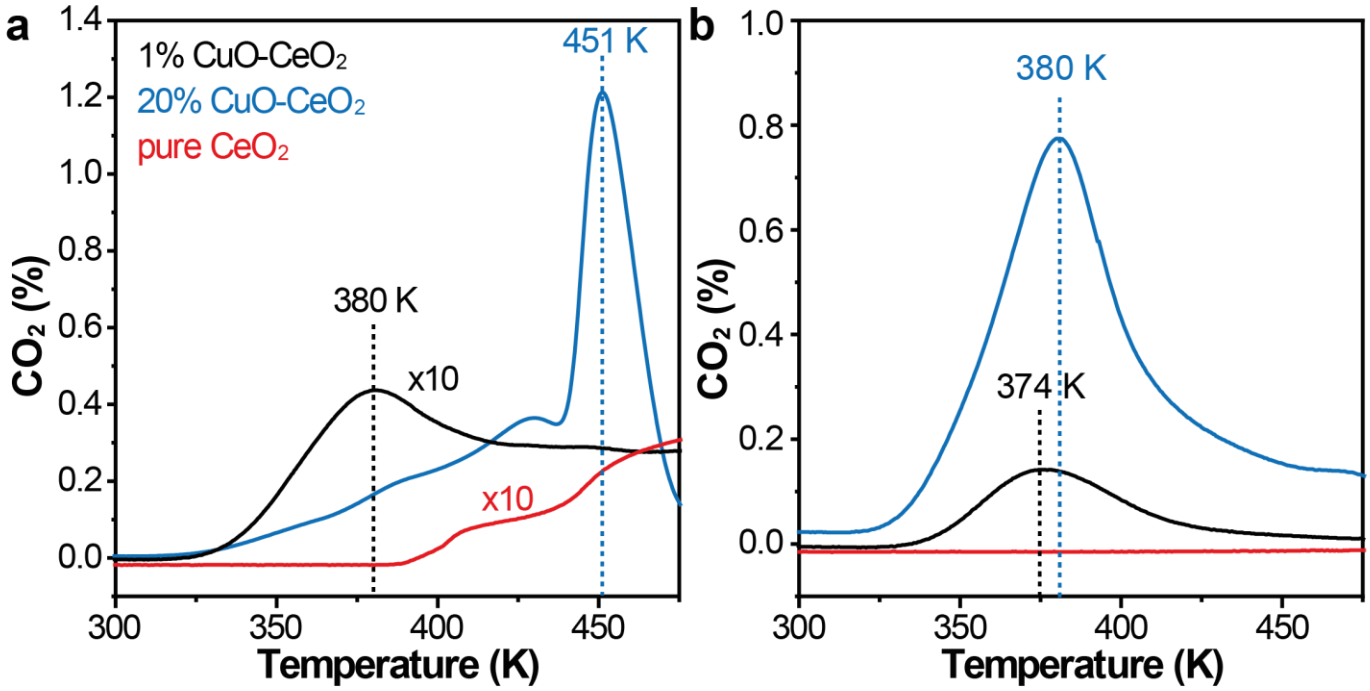

Supplementary Figure 22. The evolution profile of CO_2 during the CO temperature programmed reduction (a) and desorption (b) of 1wt% CuO-CeO_2, 20wt% CuO-CeO_2 and pure CeO_2.

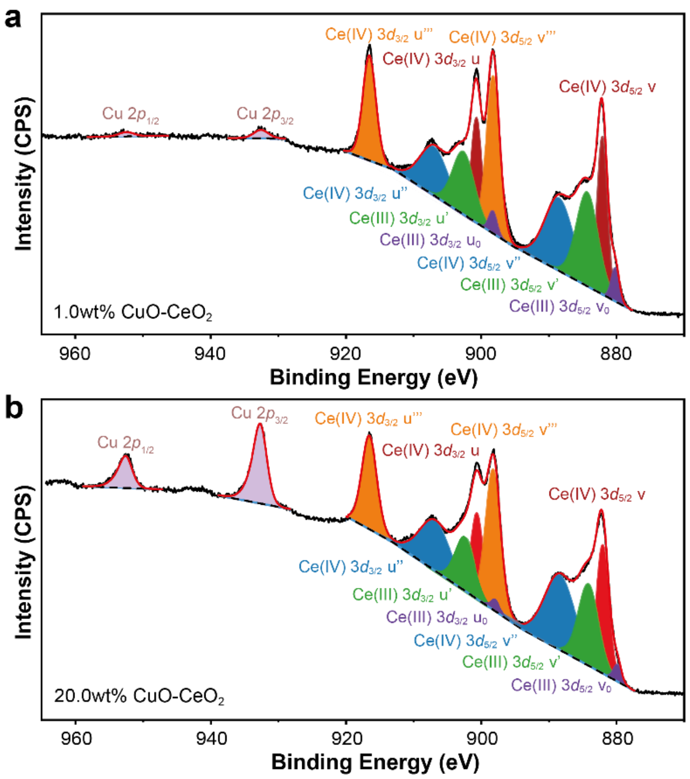

Supplementary Figure 23. XPS of 1wt% and 20wt% CuO-CeO$_2$ catalysts in Cu 2*p*$_{3/2}$ region. a, 1wt% CuO-CeO$_2$, **b**, 20wt% CuO-CeO$_2$.

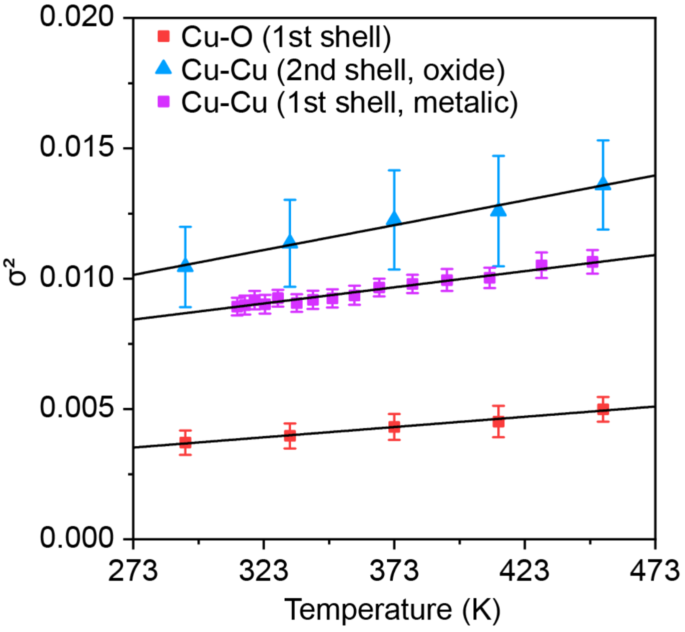

Supplementary Figure 24. Change of Debye-Waller Factor with different reaction temperatures. The changes in σ^2 with temperature for Cu-O scattering in CuO (red scatters), metallic Cu-Cu scattering (purple scatters) and Cu-Cu scattering in CuO (blue scatters) are plotted respectively. The values were obtained by fitting fresh and reduced 20wt% CuO-CeO_2 at a different temperature, while the error bars indicate the fitting errors. The same rate of changes in σ^2 value with temperature was applied to the spectra collected during the *Operando* CO oxidation experiment but starting from the σ^2 value determined from the fitting of the spectra collected at room temperature, using the same slope and intercept obtained from the linear fit.

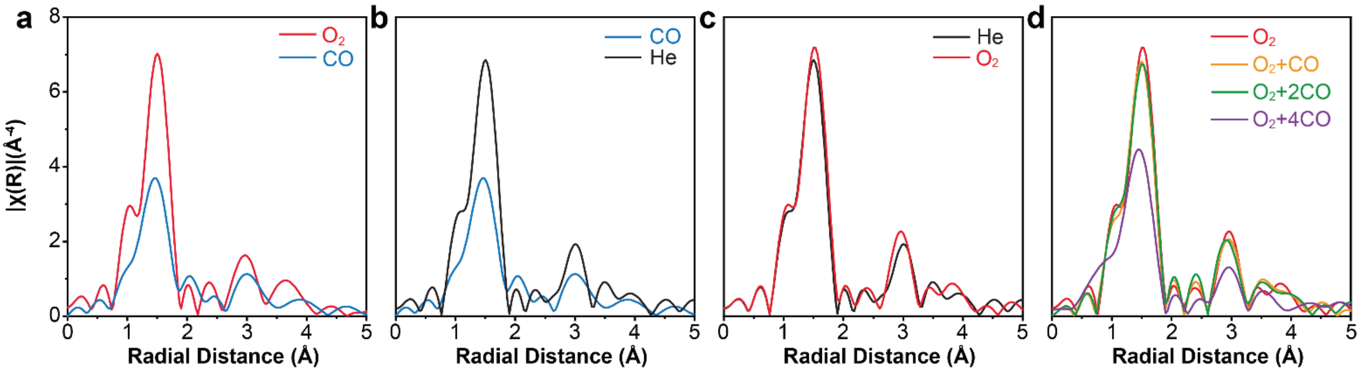

Supplementary Figure 25. ***k*^{3}-weighted R space *operando* EXAFS results of 1wt% CuO-CeO_2 catalysts at Cu K-edge.** The four figures showed the coordination environment changes (without phase correction) under same experiment condition and the gas profile of Figure 5. **a**, the reduction process by CO showed the first shell Cu-O decrease significantly, without any signal of metallic Cu-Cu scattering at 2.56Å. **b**, the reduced Cu species was re-oxidised with a significant increase in the first shell Cu-O coordination. The scattering between 3.0 Å and 4.0 Å slightly changed compared to the initial states in figure a. **c**, the further oxidation by O_2 did not make changes to the coordination, indicating the re-oxidation by CeO_2 support was sufficient. **d**, the changes of Cu species under CO lean, CO stochiometric and CO rich atmosphere. It is obvious that only under CO rich conditions would the Cu species be reduced with less Cu-O coordination.

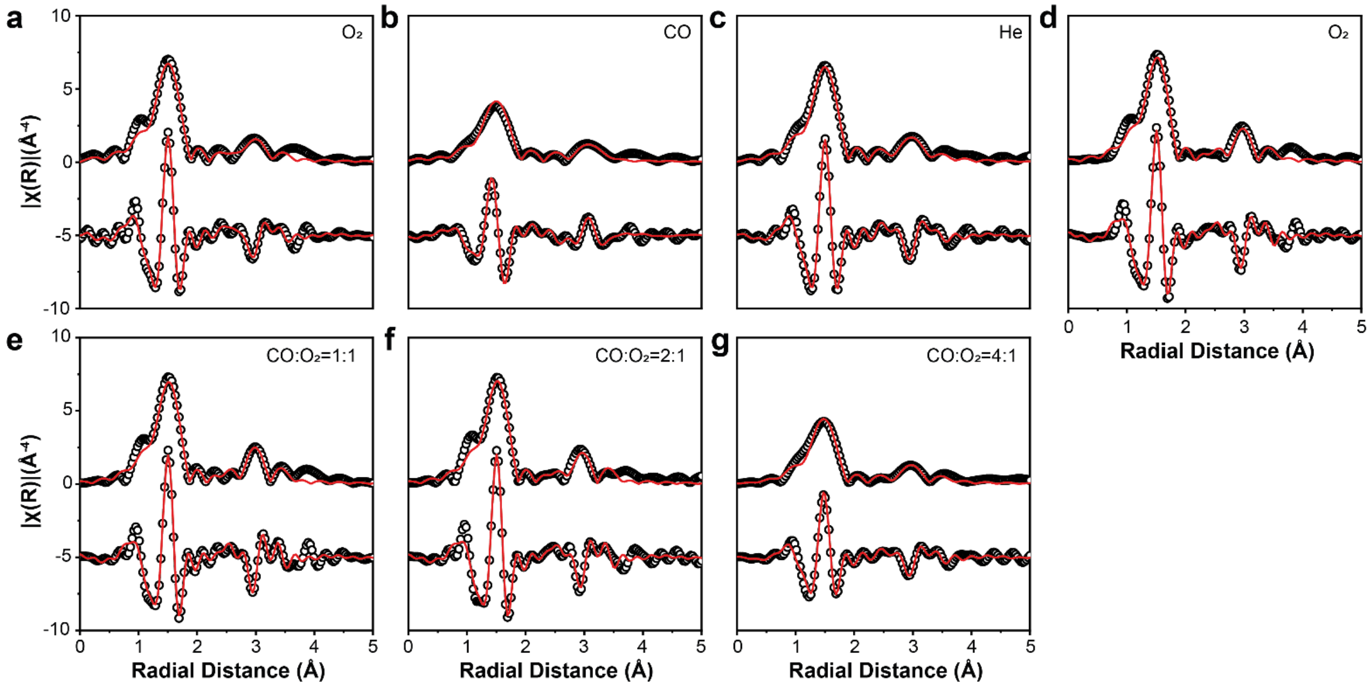

Supplementary Figure 26. ***Operando*** **EXAFS fitting results of 1wt% CuO-CeO$_2$ catalysts under different reaction conditions. a**, 1vol% O$_2$. **b**, 1vol% CO. **c**, He. **d**, 1vol% O$_2$. **e**, 0.5vol% CO + 0.5vol% O$_2$. **f**, 0.5vol% CO + 0.25vol% O$_2$. **g**, 0.5vol% CO + 0.125vol% O$_2$. The k^3-weighted Fourier Transform EXAFS data (plot without phase correction) are plotted together with fitting results (red curves). The real part of the data and fittings are showed as well.

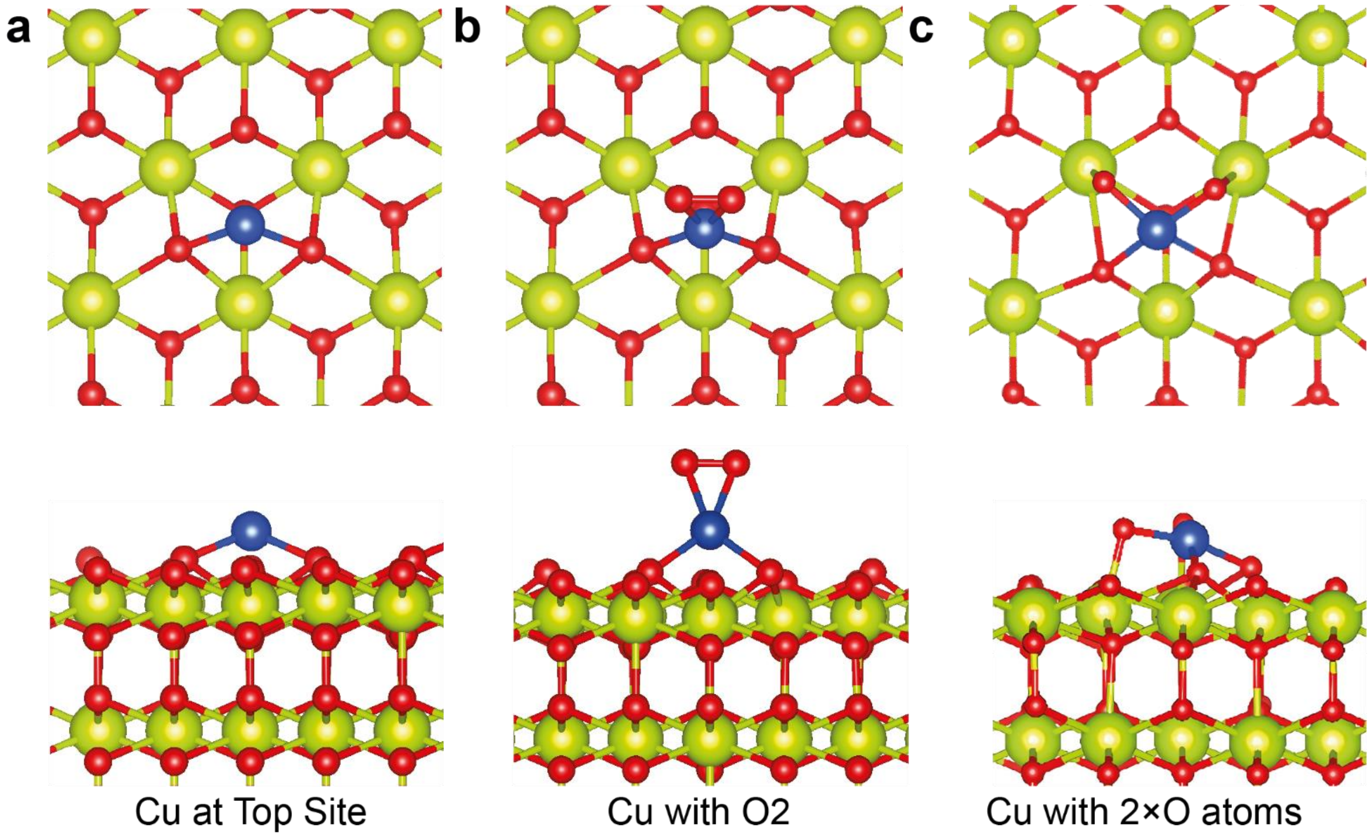

Supplementary Figure 27. Optimised structures of atomic Cu sites on CeO_2(111) surface based on DFT+*U* simulations. **a**, $[Cu(I)O_2]^{3-}$ site. **b**, Adsorption of O_2 forms $[Cu(II)O_2(\eta^2\text{-}O_2)]^{4-}$ site. **c**, Dissociated $[Cu(II)O_4]^{6-}$.

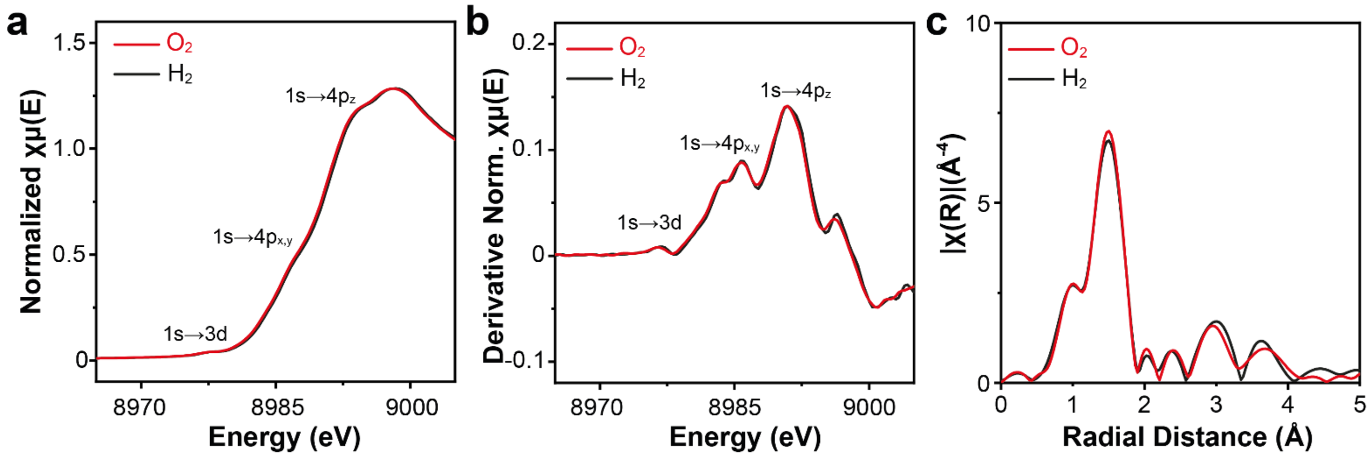

Supplementary Figure 28. *Operando* XAFS characterization of 1wt% CuO-CeO_2 under 5vol% H_2 and 10vol%O_2 at 473 K. **a**, XANES spectra. **b**, First derivative of XANES spectra. **c,** k^3-weighted R space EXAFS spectra. The oxidation states and coordination environment remained unchanged, indicating the H_2 molecules cannot be dissociated by the atomic Cu site.

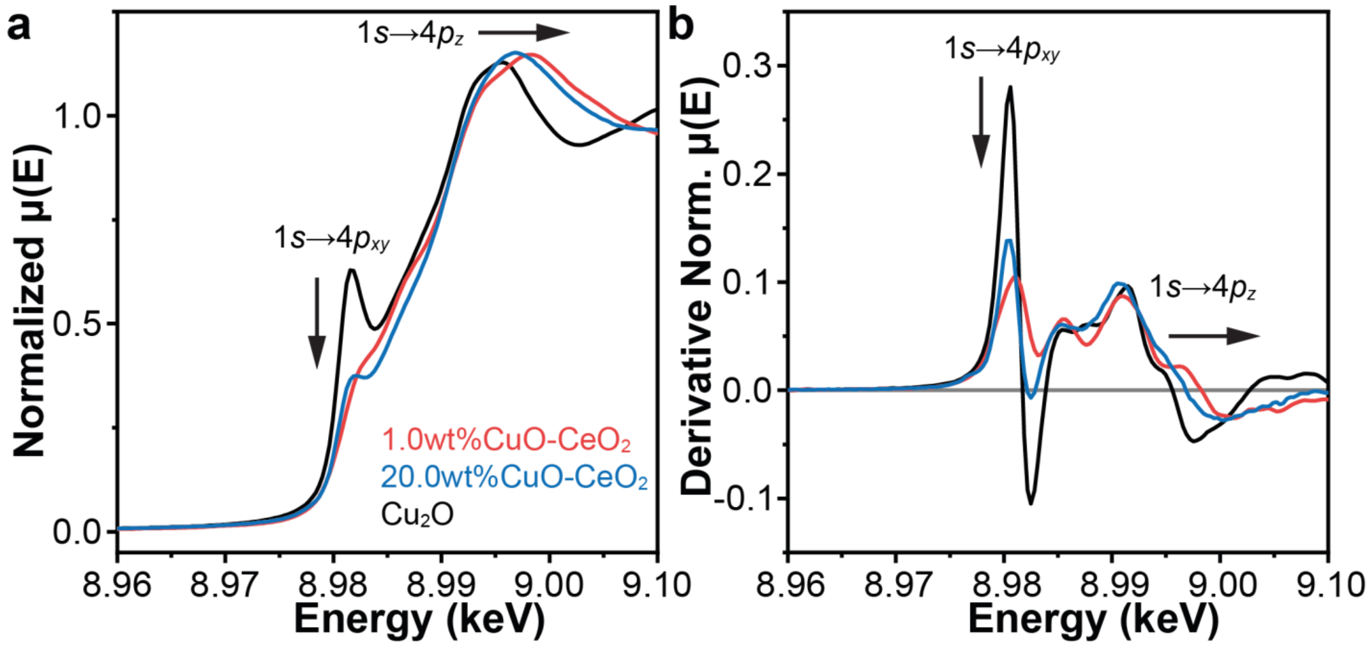

Supplementary Figure 29. XANES spectra of reduced 1wt%, reduced 20wt% CuO-CeO_2 and standard Cu_2O samples. a, Cu K-edge XANES spectra. **b**, First derivatives of XANES spectra.

Supplementary Tables

Supplementary Table 1. EXAFS fitting parameters and fitted results of $CuO-CeO_2$ catalysts[1]

Sample	Scattering	C.N.	*d* (Å)	σ^2	E_0 (eV)
Cu foil STD	Cu-Cu	12	2.56		
Cu_2O STD	Cu-O	2	1.85		
	Cu-Cu	12	3.01		
CuO STD	Cu-O	4	1.95		
	Cu-Cu	4	2.88		
		4	3.07		
		2	3.16		
0.05wt% $CuO-CeO_2$	Cu-O	3.82 ± 0.33	1.95 ± 0.01	0.004 ± 0.001	-1.50 ± 0.95
	Cu-Ce (1)	2.04 ± 0.45	3.31 ± 0.03	0.007 ± 0.003	
	Cu-Ce (2)	1.91 ± 0.48	3.49 ± 0.03		
0.4wt% $CuO-CeO_2$	Cu-O	3.89 ± 0.16	1.95 ± 0.01	0.005 ± 0.002	-1.03 ± 0.86
	Cu-Ce (1)	1.23 ± 0.51	3.34 ± 0.04	0.007 ± 0.003	
	Cu-Ce (2)	1.49 ± 0.70	3.51 ± 0.03		
0.8wt% $CuO-CeO_2$	Cu-O	3.85 ± 0.16	1.95 ± 0.01	0.005 ± 0.002	-1.64 ± 0.89
	Cu-Ce (1)	1.45 ± 0.67	3.34 ± 0.04	0.009 ± 0.005	
	Cu-Ce (2)	1.64 ± 0.73	3.51 ± 0.04		
1wt% $CuO-CeO_2$	Cu-O	3.79 ± 0.14	1.95 ± 0.01	0.005 ± 0.002	-0.68 ± 0.77
	Cu-Ce (1)	1.72 ± 0.71	3.35 ± 0.03	0.009 ± 0.006	
	Cu-Ce (2)	1.71 ± 0.70	3.51 ± 0.04		
5wt% $CuO-CeO_2$	Cu-O	4.07 ± 0.15	1.94 ± 0.01	0.005 ± 0.001	-2.27 ± 0.52
10wt% $CuO-CeO_2$	Cu-O	3.70 ± 0.24	1.95 ± 0.01	0.004 ± 0.001	-1.14 ± 0.90
	Cu-Cu (1)	0.50 ± 0.13	2.87 ± 0.04	0.008 ± 0.005	
15wt% $CuO-CeO_2$	Cu-O	3.85 ± 0.31	1.95 ± 0.01	0.005 ± 0.001	-0.53 ± 1.06
	Cu-Cu (1)	2.18 ± 0.70	2.90 ± 0.02	0.010 ± 0.005	
	Cu-Cu (2)	1.31 ± 0.91	3.12 ± 0.04		
20wt% $CuO-CeO_2$	Cu-O	3.74 ± 0.09	1.94 ± 0.01	0.005 ± 0.001	-0.71 ± 0.31
	Cu-Cu (1)	3.82 ± 0.60	2.90 ± 0.01	0.010 ± 0.005	
	Cu-Cu (2)	2.84 ± 0.53	3.12 ± 0.02		

[1] The calculated amplitude reduction factor S_0^2 from EXAFS analysis of Cu foil was 0.809, which was used as a fixed parameter for EXAFS fitting. The coordination number (C.N.) and bond length (d) were calculated based on standard crystal structure previously reported from Crystal open database: Copper (No. 9013014), Cuprite (No. 1010963) and Tenorite (No. 1011148).

Supplementary Table 2. Peak position of XANES spectra of CuO-CeO_2.

Sample	Absorption peak position (keV)					White line position (keV)
	1*s* to 3*d* [a]	1*s* to $4p_z$ [a]	1*s* to $4p_{xy}$ [a]	1*s* to $4p_z$ [b]	1*s* to 4*p* [b]	
Cu		8979.0				8993.5
Cu (HERFD)		8979.0				
Cu_2O		8980.6			8981.6	8995.6
Cu_2O (HERFD)		8980.6			8981.6	8995.6
CuO	8977.1	8983.7	8990.8	8986.0		8997.5
CuO (HERFD)	8977.2	8983.7	8988.6	8985.4		8997.1
0.05wt%	8976.6	8985.9	8991.3	8988.0		8998.3
0.4wt%	8976.7	8985.9	8991.4	8987.9		8998.2
0.8wt%	8976.5	8986.0	8991.4	8987.9		8998.2
1wt%	8976.5	8985.9	8991.3	8987.9		8998.1
1wt% (HERFD)	8976.9	8985.9	8991.3	8987.8		8998.0
5wt%	8976.5	8985.6	8991.2	8987.5		8997.8
10wt%	8976.5	8985.5	8991.1	8987.4		8997.6
15wt%	8976.8	8984.0	8990.9	8987.1		8997.6
20wt%	8977.2	8983.7	8990.8	8986.7		8997.5
20wt% (HERFD)	8977.2	8983.6	8990.7	8986.5		8997.4
1wt% reduced		8980.9			8983.1	8998.2
20wt% reduced		8980.6			8982.1	8996.9

[a] Peak position in first derivative XANES.
[b] Peak position in XANES.

Supplementary Table 3. Spin Hamiltonian parameters from EPR simulation. The Spin Hamiltonian parameters of Cu^{2+} (S=1/2) species, obtained from the simulations of the 0.05wt% CuO-CeO_2 EPR spectra at 298 K and the operando EPR records of 1wt% CuO-CeO_2 at 393 K.

Signal	g_z	g_y	g_x	Az (MHz)	Ay (MHz)	Ax (MHz)
	$g_{\parallel}$	$g_{\perp}$		A‖ (MHz)	A⊥(MHz)	
A1	2.327	2.048		372	55	
B1		<g> = 2.10 [a]				
B2	2.280 [a]	2.132 [a]	2.051 [a]	470	35 [a]	35 [a]
C1	2.293	2.036		402	92	
C2	2.275	2.050		492	34	

[a] Broad signal, estimated values with large uncertainty.

Supplementary Table 4. Geometric and electronic properties of Cu on CeO_2(111) surface. [a]

Surface state	C.N.	E_{ads} (eV)	Q_{Cu}	*d*(Cu-O) (Å)	*d*(Cu-Ce) (Å)
Cu at bridge site	2	Cu: -2.43	+0.62 (+0.58)	Sur: 1.95 × 2, (1.89 × 2)	2.76, 3.06 × 2
Cu with adsorbed O_2	4	O_2: -0.68	+0.91	Sur: 2.00 × 2, Ads: 1.98 × 2	2.94, 3.35 × 2
Cu with double adsorbed O atoms	4	per O: -3.14	+1.08	Sur: 1.94 × 2, Ads: 1.81 × 2	2.91, 2.95, 3.21

[a] C.N.: the coordination number of Cu atom; E_{ads}: the adsorption energy with respect to the isolated Cu atom and the bare slab model of CeO_2(111); Q_{Cu}: calculated effective charge on Cu atom, *d*(Cu-O): the distance between the Cu atom and the bonding O atoms, Sur: surface oxygen, Ads: adsorbed oxygen; *d*(Cu-Ce): distance between the Cu atom and the nearest surface Ce atoms. (data between parentheses correspond to the ref. [4]).

Supplementary Table 5. Geometric and electronic properties of Cu on CeO_2(110) surface. [a]

Surface state	C.N.	E_{ads} (eV)	Q_{Cu}	*d*(Cu-O) (Å)	*d*(Cu-Ce) (Å)
Cu at top site	2	Cu: -3.26	+0.48	Sur: 1.75, 1.79	2.92, 3.42 × 2 (3.30)
Cu with adsorbed O_2	4	O_2: -1.25	+0.94	Sur: 1.86 Ads: 1.85	3.32, 3.67 × 2
Cu with double adsorbed O atoms	4	per O: -4.86	+1.12	Sur: 1.86 × 2, Ads: 1.81 × 2	3.12, 3.19 × 2

[a] C.N.: the coordination number of Cu atom; E_{ads}: the adsorption energy with respect to the isolated Cu atom and the bare slab model of CeO_2(110); Q_{Cu}: calculated effective charge on Cu atom, *d*(Cu-O): the distance between the Cu atom and the bonding O atoms, Sur: surface oxygen, Ads: adsorbed oxygen; *d*(Cu-Ce): distance between the Cu atom and the nearest surface Ce atoms. (data between parentheses correspond to the ref. [5]).

Supplementary Table 6. Bader charge analysis results of CeO_2(111). The model contains 27 Ce and 54 O atoms. [a]

Element	Valence Electrons	Charge
Ce	9.600	+2.400
O	7.200	-1.200

[a] In the charge of elements, '+' and '-' denote positive and negative charges relative to the valence electron of neutral atoms (Ce: 12 and O: 6).

Supplementary Table 7. Bader charge analysis results of atomic Cu(I) site on CeO_2(111). The model contains 27 Ce, 54 O and 1 Cu atoms. [a]

Element	Valence Electrons	Charge	Difference from the Reference	Charge Transfer
Ce	9.619	+2.3806	-0.0194 [b]	-0.524
O	7.202	-1.2017	-0.0017 [b]	-0.092
Cu	10.383	+0.617	+0.617 [c]	+0.617

[a] Charge Transfer is calculated based on the number of lost or gained electrons relative to the valence electron of neutral atoms (Ce: 12; O: 6 and Cu: 11). - denotes gaining electrons, while + is losing electrons. In total, 27 Ce gain 27 × - 0.0194 = - 0.524 and 54 O gain 54 × - 0.0017 = - 0.092. [b] Compared with pure CeO_2. [c] Compared with neutral Cu atom.

Supplementary Table 8. Bader charge analysis results of atomic Cu(II) site on CeO_2(111). The model contains 27 Ce, 54 O and 1 Cu atoms. [a]

Element	Valence Electrons	Charge	Difference from the Reference	Charge Transfer
Ce	9.612	+2.3883	-0.0117 [b]	-0.316
O	7.175	-1.1920	+0.008 [b]	+0.432
O_{ads}	6.595	-0.595	-0.595 [c]	-1.190
Cu	9.926	+1.074	+1.074 [d]	+1.074

[a] Charge Transfer is calculated based on the number of lost or gained electrons relative to the valence electron of neutral atoms (Ce: 12; O: 6 and Cu: 11). + denotes gaining electrons, while - is losing electrons. In total, 27 Ce gain 27 × - 0.0117 = - 0.316 and 54 O lose 54 × + 0.0008 = + 0.432. Two O_{ads} gain 2 × - 0.595 = - 1.190 in total. [b] Compared with pure CeO_2. [c] Compared with neutral O atom. [d] Compared with neutral Cu atom.

Supplementary Table 9. The Bader charge analysis results of Cu(I) and Cu(II)

Species	Model	Bader Charge	Difference
Cu(I)	$Cu_2O(111)$	+0.495	+0.122
	Cu(I) single-site on $CeO_2(111)$	+0.617	
Cu(II)	CuO(111)	+1.001	+0.063
	Cu(II) single-site on $CeO_2(111)$	+1.074	

Supplementary Table 10. Deconvolution results for the XPS of 1wt% and 20wt% CuO-CeO₂ catalysts.

				**1wt% CuO-CeO$_2$**			**20wt% CuO-CeO$_2$**		
**Species**	**Peak**	**label**	**Final state**	**B.E. [eV]**	**Area**		**B.E. [eV]**	**Area**	
**Ce (III)**	$3d_{5/2}$	v_0	Ce $3d^9 4f^2$ O $2p^5$	880.2	2.0%	28.6%	879.9	1.0%	22.8%
	$3d_{3/2}$	u_0		898.3	1.3%		898.0	0.7%	
	$3d_{5/2}$	v'	Ce $3d^9 4f^1$ O $2p^6$	884.3	15.1%		884.2	12.6%	
	$3d_{3/2}$	u'		902.6	10.1%		902.5	8.4%	
**Ce (IV)**	$3d_{5/2}$	v	Ce $3d^9 4f^0$ O $2p^6$	882.1	11.8%	71.4%	882.0	12.1%	77.2%
	$3d_{3/2}$	u		900.7	7.9%		900.6	8.1%	
	$3d_{5/2}$	v''	Ce $3d^9 4f^1$ O $2p^5$	888.5	15.5%		888.4	17.9%	
	$3d_{3/2}$	u''		907.0	10.4%		906.9	12.0%	
	$3d_{5/2}$	v'''	Ce $3d^9 4f^2$ O $2p^4$	898.2	15.5%		898.3	16.3%	
	$3d_{3/2}$	u'''		916.5	10.4%		916.6	10.9%	
**Cu**	$2p_{3/2}$	N/A	Cu $2p^5$ O $2p^6$	932.6	66.7%		932.7	66.7%	
	$2p_{1/2}$			952.5	33.3%		952.6	33.3%	

Supplementary Table 11. *Operando* EXAFS fitting parameters and fitted results of 1wt% CuO-CeO_2 catalysts.

Reaction Condition	Scattering	C.N.	*d* (Å)	σ^2	E_0 (eV)
453K<br>1vol% O_2	Cu-O	3.68 ± 0.18	1.94 ± 0.01	0.005	-2.04 ± 1.01
	Cu-Ce (1)	3.00 ± 1.41	3.30 ± 0.03	0.013 ± 0.006	
	Cu-Ce (2)	2.04 ± 0.97	3.51 ± 0.06		
453K<br>1vol% CO	Cu-O	2.03 ± 0.11	1.90 ± 0.01	0.005	-6.93 ± 1.20
	Cu-Ce (1)	1.46 ± 1.16	3.29 ± 0.06	0.010 ± 0.009	
	Cu-Ce (2)	0.70 ± 0.46	3.47 ± 0.11		
453K<br>He	Cu-O	3.62 ± 0.18	1.94 ± 0.01	0.005	-1.74 ± 1.01
	Cu-Ce (1)	2.91 ± 1.29	3.30 ± 0.03	0.011 ± 0.006	
	Cu-Ce (2)	1.94 ± 0.82	3.50 ± 0.05		
453K<br>1vol% O_2	Cu-O	3.80 ± 0.18	1.94 ± 0.01	0.005	-1.78 ± 0.91
	Cu-Ce (1)	2.78 ± 0.93	3.29 ± 0.02	0.010 ± 0.004	
	Cu-Ce (2)	1.97 ± 0.72	3.48 ± 0.04		
453K<br>0.5vol% O_2 +<br>0.5vol% CO	Cu-O	3.68 ± 0.17	1.94 ± 0.01	0.005	-1.61 ± 0.88
	Cu-Ce (1)	3.14 ± 0.93	3.29 ± 0.02	0.010 ± 0.003	
	Cu-Ce (2)	2.14 ± 0.76	3.50 ± 0.03		
453K<br>0.25vol% O_2 +<br>0.5vol% CO	Cu-O	3.71 ± 0.17	1.95 ± 0.01	0.005	-1.48 ± 0.90
	Cu-Ce (1)	2.98 ± 0.96	3.29 ± 0.02	0.011 ± 0.004	
	Cu-Ce (2)	2.33 ± 0.83	3.28 ± 0.03		
453K<br>0.125vol% O_2 +<br>0.5vol% CO	Cu-O	2.43 ± 0.13	1.92 ± 0.01	0.005	-1.86 ± 1.10
	Cu-Ce (1)	1.97 ± 0.79	3.30 ± 0.03	0.010 ± 0.005	
	Cu-Ce (2)	1.40 ± 0.60	3.50 ± 0.04		

Supplementary Table 12. The valence band maximum (VBM) of Cu(I) single-site and $Cu_2O(111)$ surface.

Model	VBM (eV)
$Cu_2O(111)$	-0.2244
Cu(I) single-site on $CeO_2(111)$	-0.9451

Supplementary Table 13. Comparison of the CO oxidation activity of various active metal sites.

<table>
<tr><th rowspan="2">Active Sites</th><th colspan="2">Gas</th><th rowspan="2">Conversion</th><th rowspan="2">WHSV [a] ($mL \cdot g_{cat}^{-1} \cdot h^{-1}$)</th><th rowspan="2">TOF at ($x10^{-2}$ s^{-1})</th></tr>
<tr><th>[CO] vol%</th><th>[O_2] vol%</th></tr>
<tr><td>Atomic Cu sites on CeO_2 [b]</td><td rowspan="2">1%</td><td rowspan="2">10%</td><td>20.9%</td><td rowspan="2">120,000</td><td>2.48</td></tr>
<tr><td>Cu clusters on CeO_2 [c]</td><td>4.0%</td><td>0.47</td></tr>
<tr><td>12mol% CuO on CeO_2[6]</td><td>1%</td><td>1%</td><td>50.0% [d]</td><td>120,000</td><td>1.01</td></tr>
<tr><td>$Cu_{0.1}CeO_{2-x}$[7]</td><td>1%</td><td>2.5%</td><td>10.0% [d]</td><td>78,000</td><td>0.16</td></tr>
<tr><td>9mol% CuO on CeO_2[8]</td><td>1%</td><td>10%</td><td>35.0% [d]</td><td>60,000</td><td>0.47</td></tr>
<tr><td>Isolated Pt sites on CeO_2[9]</td><td>0.4%</td><td>10%</td><td>12.6% [d]</td><td>200,000</td><td>2.43</td></tr>
<tr><td>Pt_{iso}/TiO_2 reduced at 523 K[10]</td><td rowspan="2">1%</td><td rowspan="2">1%</td><td>0.03%</td><td rowspan="2">480,000</td><td>0.14 [e]</td></tr>
<tr><td>Pt_{iso}/TiO_2 reduced at 723 K[10]</td><td>0.11%</td><td>0.49 [e]</td></tr>
<tr><td>Pt_1/CeO_2-a[11]</td><td rowspan="2">0.1%</td><td rowspan="2">5%</td><td>0.8%</td><td rowspan="2">2,400,000</td><td>1.7 [f]</td></tr>
<tr><td>Pt-O-Pt/CeO_2-a[11]</td><td>91.9%</td><td>197 [f]</td></tr>
</table>

[a] WHSV = Weight hourly space velocity per gram of catalysts. [b] Reactivity of 1wt% CuO-CeO_2 at 373 K in this work. [c] Reactivity of 20wt% CuO-CeO_2 at 373 K in this work. [d] The conversion data at 373 K read from the published work. [e] The reactivity data obtained at 423 K in the published work, data at 373 K is not shown by the authors. [f] The reactivity data obtained at 423 K shown in the published work, the reactivity of Pt_1/CeO_2-a at 373 K is too low for comparison with Pt-O-Pt/CeO_2-a.

Supplementary Note 2. The reactivity of single sites in oxidation might be further improved if a more suitable structure can be adopted. The enhanced activity of isolated active sites in oxidation and the comparison of various turnover frequency (TOF) with Cu-based and Pt-based catalysts are discussed below.

The atomic Cu(I) sites studied in this work are favourable in the formation of an electrophilic η^2-O_2 species (Fig. 4c), which is rarely reported with other Cu-based catalysts. We hypothesise that such electrophilic η^2-O_2 species can attract electrons from the CO, thus improve the activity of CO oxidation.

In CO oxidation, we demonstrate that the isolated Cu(I)/(II) sites are ten times more active than CuO clusters in terms of TOF (Fig. 3c). The TOF in CO oxidation of atomic Cu sites (2.48×10^{-2} s^{-1}) is also higher than that of Cu-based catalysts in the literature and is comparable with that of the isolated Pt sites on CeO_2[9] (2.43×10^{-2} s^{-1}, Supplementary Table 11). Below 1wt% CuO loading, the CuO-CeO_2 catalysts exhibit similar TOF and *E*a, indicating identical isolated active sites with the same intrinsic activity. When the CuO loading is above 1wt%, the catalytic activity of the CuO-CeO_2 catalysts declines as reflected by the decreased TOF and increased *E*a. The continuous decrease of the normalised activity suggests that the cluster sites are generally less active compared with isolated Cu sites.

The Pt-O-Pt dimers that built from isolated Pt sites on CeO_2 exhibit 100-1000 times TOF (197×10^{-2} vs 1.7×10^{-2} s^{-1}) and much lower activation energy (40 vs 86 $kJ\cdot mol^{-1}$) in CO oxidation.[11] This work also

claims that dinuclear Pt-O-Pt structure is not always more active than atomic Pt species in oxidation since the exceptionally active Pt-O-Pt dimers in this work rely on special coordination environment and high dispersion on ceria. Similar activity improvement of atomically dispersed Pt-TiO_2 catalysts is also found when the isolated Pt single-site (0.14×10^{-2} s^{-1}, 78 $kJ\cdot mol^{-1}$) was converted into an atomic Pt-OH site (0.49×10^{-2} s^{-1}, 48 $kJ\cdot mol^{-1}$) after being reduced at 723 K in 5% H_2/Ar.[10] The following work on similar Pt-CeO_2 catalysts found that the isolated Pt species with low loadings exhibited high resistance to sintering up to 773 K in H_2 as these Pt species preferentially occupied the most thermodynamically stable adsorption sites on ceria.[12] Therefore, the catalytic behaviour of metal single sites highly relies on the inherent surface property of supports and local coordination environment. The most active structure could vary with different combinations of active metal centres and support materials.

Supplementary References

1. Kais, A. A., Bennani, A., Aissi, C. F., Wrobel, G. & Guelton, M. Reduction effect on cerium oxide catalysts doped with copper(II) ions - an electron-paramagnetic resonance study. *J. Chem. Soc. Faraday Trans.* **88**, 1321-1325 (1992).
2. Wang, F. *et al. In situ* EPR study of the redox properties of CuO-CeO_2 catalysts for preferential CO oxidation (PROX). *ACS Catal.* **6**, 3520-3530 (2016).
3. Mehran, F., Barnes, S. E., Chandrashekhar, G. V., Mcguire, T. R. & Shafer, M. W. Absence of excited triplets in the EPR of the high-T_c superconductors and the antiferromagnetic insulator CuO. *Solid State Commun.* **67**, 1187-1189 (1988).
4. Branda, M. M., Hernandez, N. C., Sanz, J. F. & Illas, F. Density functional theory study of the interaction of Cu, Ag, and Au atoms with the regular CeO_2 (111) surface. *J. Phys. Chem. C* **114**, 1934-1941 (2010).
5. Chutia, A. *et al.* The adsorption of Cu on the CeO_2(110) surface. *Phys. Chem. Chem. Phys.* **19**, 27191-27203 (2017).
6. Luo, M.-F., Ma, J.-M., Lu, J.-Q., Song, Y.-P. & Wang, Y.-J. High-surface area CuO–CeO_2 catalysts prepared by a surfactant-templated method for low-temperature CO oxidation. *J. Catal.* **246**, 52-59 (2007).
7. Elias, J. S., Risch, M., Giordano, L., Mansour, A. N. & Shao-Horn, Y. Structure, Bonding, and Catalytic Activity of Monodisperse, Transition-Metal-Substituted CeO_2 Nanoparticles. *J. Am. Chem. Soc.* **136**, 17193-17200 (2014).
8. Chen, G. *et al.* Facile and Mild Strategy to Construct Mesoporous CeO_2–CuO Nanorods with Enhanced Catalytic Activity toward CO Oxidation. *ACS Appl. Mater. Interfaces* **7**, 23538-23544 (2015).
9. Nie, L. *et al.* Activation of surface lattice oxygen in single-atom Pt/CeO_2 for low-temperature CO oxidation. *Science* **358**, 1419-1423 (2017).
10. DeRita, L. *et al.* Structural evolution of atomically dispersed Pt catalysts dictates reactivity. *Nat. Mater.* **18**, 746-751 (2019).
11. Wang, H. *et al.* Surpassing the single-atom catalytic activity limit through paired Pt-O-Pt ensemble built from isolated Pt1 atoms. *Nat. Commun.* **10**, 3808 (2019).
12. Resasco, J. *et al.* Uniformity Is Key in Defining Structure–Function Relationships for Atomically Dispersed Metal Catalysts: The Case of Pt/CeO_2. *J. Am. Chem. Soc.* **142**, 169-184 (2019).
