# Supplementary material for: Adsorption and activation of molecular oxygen over atomic copper(I/II) site on ceria
Source: Nat Commun. 2020 Aug 11;11:4008. doi: 10.1038/s41467-020-17852-8 (PMC7419315; doi:10.1038/s41467-020-17852-8)
Supplement: Supplementary file 2 — Peer Review File [file 41467_2020_17852_MOESM2_ESM.pdf]

Reviewers' comments:

Reviewer #1 (Remarks to the Author):

In this work, the authors have synthesized Cu doped CeO_2 nanoparticles with different weight loadings via flame spray pyrolysis. They have tried to selectively activate O_2 through nanoparticles with low weight loadings of Cu. Through operando XAS, EPR, NAP-NEXAFS, and DFT calculations, the authors have concluded that it is the well-dispersed $[Cu(I)O_2]^{3-}$ site that plays an essential role in selective activation of O_2. However, there are some inconsistent results reported in the manuscript. In addition, solid justifications for the main conclusion should be further provided. Thus, I have to reserve my recommendation for its publication in Nature Communications. Specifically:

Major points:

1.In line 75-81 (or Figure 1), the authors suggested that the increasing HOMO and LUMO gap from bulk CuO to CuO cluster and to atomic Cu(I/II) sites is due to the electron withdrawing effect from Ce^{4+}. This may be right. However, the size effect of the CuO clusters can also influence the HOMO and LUMO gap. Although Ce^{4+} can withdraw electrons from metallic Cu(0) (ref 28), it doesn't mean that Ce^{4+} can withdraw electrons from Cu(II).

2.The causality between the HOMO energy and the activity of the Cu sites are not clear. Considering the MvK mechanism is the most widely accepted mechanism (Chem. Rev. 113, 4391–4427 (2013), the highest activity of the atomic dispersed Cu(I/II) sites would be due to the observation that "CeO_2 is easier to be reduced under UHV with atomic Cu site (line 254-255)". To support the main conclusion of the current manuscript, the authors should provide more convincing experimental or theoretical investigations revealing the causality between the HOMO energy and the activity of the Cu sites.

3.In line 74-75 (or Figure 1), the authors stated that "the surface atomic metal site has discrete electronic structures compared with the continuous band structures found in clusters." And line 82-84 the discrete electronic structures of Cu on Ag/Cu systems were mentioned again. Why are the discrete electronic structures important, which shall be emphasized?

4.The authors assigned discrete HOMO as occupied 3d orbitals of Cu (Figure 1). However, the transitions of 1s → 3d shown in Figure 2e indicated that 3d orbitals are not fully occupied and/or not discrete. The authors should provide justification for their description of discrete HOMO.

5.In Figure 4, the authors assigned the pure Cu(II) species as $[Cu(II)O_4]^{6-}$, where all oxygen atoms become lattice O^{2-}. However, according to the DFT calculations shown in Supplementary Figure 15, this structure is 1.05 eV less stable than $[Cu(II)O_2(\eta^2\text{-}O_2)]^{4-}$. The authors should provide justification for the existence of the unstable Cu(II) species.

6.The selective activation of O_2 was not well defined and determined in this work. The selectivity can be among different activated oxygen species, like O2-, O2-, O22-. The selectivity also can be among the activation of O_2 and the other reactants.

Minor points:

1.Line 61, "group 8" should be "group 10" or "group VIII".
2.Line 69, "d9 and d10" should be "d10 and d9".
3.Line 81, the author state that "the energy of the LUMO increases, weakening its propensity to form a bond with O_2". Because O_2 often plays as an oxidant, this statement should be revised.
4.Line 93, Refs. 31 and 32 were cited for the $[Cu(II)O_2(\eta^2\text{-}O_2)]^{4-}$. However, both references are related to Ag rather than Cu. The proper references should be cited here.
5.In Figure 2h, the smallest wt% is less than 0, which should be corrected.

6.Line 163, the "tetragonally distorted octahedral" structure should be clarified.
7.Line 243, the Raman band located around 830 cm-1 in Supplementary Fig. 13 is associated with the η2-O2. What is the more intense shoulder band around 600 cm-1?
8.In Supplementary Materials line 208, the method for PDOS was provided. However, there is no PDOS reported in this work.
9.In Supplementary Materials line 369, is it "CeO2(111)" or "CeO2(110)"?

Reviewer #2 (Remarks to the Author):

The authors describe an interesting study on the activation of dioxygen at isolated Cu single sites supported on ceria by a multitechnique approach combining operando XAS, EPR, NAP-NEXAFS and DFT simulations. They provided evidence for the presence of an electrophilic [Cu(II)O2(η2-O2)]4- species as a key intermediate state that should account for the high catalytic activity and selectivity of related oxidation reactions. In particular, the authors proposed the concept of using electronic structures to control the catalytic performance. Overall, these results allow to gain a fundamental insight into the structural, electronic and reactive properties of ceria-supported Cu single site catalysts. This work could be interesting to a broad readership. However, I cannot recommend this manuscript for publication in Nature Commun. in its current form. I have the following major concerns:

1) With regards to the stable structure of Cu single atoms on ceria, the DFT simulations are insufficiently described. It is known that the morphologies of different ceria surfaces play a crucial role in the interaction between metal and support. As mentioned in the paper, the surface Ce3+ species are involved in the activation of dioxygen. In this context, the oxygen vacancy formation energy depends strongly on the orientation of ceria surfaces (see e.g., Chem. Rev., 2013, 113, 3949). The surface structures of the ceria NPs should be taken into account.
2) In addition to Cu adatoms, single Cu atoms also could be stabilized by substituting a cerium atom on the surface and/or in the bulk under oxidative conditions.
3) A number of groups have reported that metal single sites are not active for oxidation reactions (see e.g. Nat. Commun. 2019, 10: 3808; Nat. Mater. 2019, 18, 746). A recent work showed that the ultrafine Cu clusters (bilayer) on ceria account for the high catalytic activity for the water gas shift reaction (Nat. Catal. 2019, 2, 334).
4) For the CO oxidation reaction, a synergistic effect should be more important than the activation of single reactant, i.e., a site cooperation allowing the activation of both CO and O2.

Response to Referee 1:

To the comments of Reviewer 1

Overall comments: In this work, the authors have synthesized Cu doped CeO_2 nanoparticles with different weight loadings via flame spray pyrolysis. They have tried to selectively activate O_2 through nanoparticles with low weight loadings of Cu. Through operando XAS, EPR, NAP-NEXAFS, and DFT calculations, the authors have concluded that it is the well-dispersed $[Cu(I)O_2]^{3-}$ site that plays an essential role in selective activation of O_2. However, there are some inconsistent results reported in the manuscript. In addition, solid justifications for the main conclusion should be further provided. Thus, I have to reserve my recommendation for its publication in Nature Communications. Specifically:

We thank this referee for the insightful comments. We address all the issues below to explain the results and justify the main conclusion.

Major points:

Question 1: In line 75-81 (or Figure 1), the authors suggested that the increasing HOMO and LUMO gap from bulk CuO to CuO cluster and to atomic Cu(I/II) sites is due to the electron withdrawing effect from Ce^{4+}. This may be right. However, the size effect of the CuO clusters can also influence the HOMO and LUMO gap. Although Ce^{4+} can withdraw electrons from metallic Cu(0) (ref 28), it doesn't mean that Ce^{4+} can withdraw electrons from Cu(II).

Response 1: We thank the reviewer for the comments on the size effect and the electron withdrawing effect on Ce^{4+}. We have explained the size effect more clearly and performed additional calculations to show the Ce^{4+} ions do withdraw electrons from the system.

The size of CuO plays a very important role in the HOMO/LUMO gap. We acknowledge this in line 78-80 that "The energy splitting between the highest occupied molecular orbital (HOMO) and the lowest unoccupied molecular orbital (LUMO) of Cu species is therefore increased by the interaction between Cu and Ce (Fig. 1), and is maximised in isolated and atomically dispersed Cu sites,". This is because that, when the size of CuO is decreasing, the number of Cu-O-Cu bonds decreases and the number of Cu-O-Ce bond increases.

To strengthen the role of size effect, we add the following sentence at the paragraph before Fig. 2.

"Thus, the decrease of CuO size leads to the stronger interaction between Cu-O-Ce and less interaction between Cu-O-Cu, increasing the gap between HOMO and LUMO."

In order to further prove the electron withdrawing effect of Ce^{4+} to atomic Cu(I/II) sites, we use Bader charge analysis to evaluate the charge transfer among Cu, O and Ce for the $[Cu(I)O_2]^{3-}$ and $[Cu(II)O_4]^{6-}$ sites.

A pure CeO_2 (111) model which comprises 27 Ce atoms and 54 O atoms is utilised as a

reference (Table. X1). The calculated charge of Ce and O are +2.40 and -1.20, respectively.

Table X1. Bader charge analysis results of CeO_2(111). [a]

Element	Valence Electrons	Charge
Ce	9.600	+2.400
O	7.200	-1.200

[a] In the charge of elements, '+' and '-' denote positive and negative charges relative to the valence electron of neutral atoms (Ce: 12 and O: 6).

According to the Charge Density Difference (CDD) plot of atomic Cu(I) on CeO_2(111) (Fig. X1) which simulates the $[Cu(I)O_2]^{3-}$ site (Fig. 4a inset and Supplementary Table 4), Cu is the only electron donor (-0.617 charge) (Table X2). On average, each 27 Ce atoms receive -0.0194 charge, with the total charge transfer from Cu to Ce at -0.524. Though the charge transfer to individual Ce atom cannot be calculated, we hypothesise that the neighbouring Ce atoms receive more charges than Ce atoms which are further away with Cu. In comparison, the total charge transfer to 54 O atoms is -0.092. The electron withdrawing effect of Ce^{4+} is clearly validated by the fact that 85% of the negative charge from Cu is transferred to Ce^{4+} (+0.524).

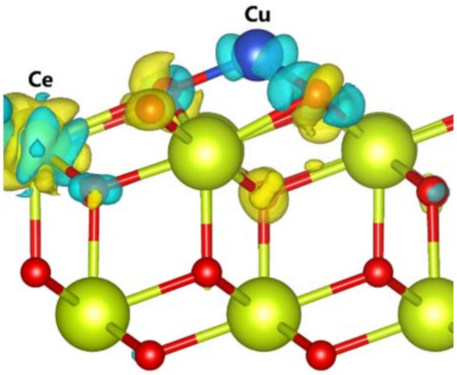

Figure X1. The CDD plot of atomic Cu(I) on CeO_2(111). In the CDD plot, yellow isosurfaces denote electron gain and blue isosurfaces mean the electron lost.

Table X2. Bader charge analysis results of atomic Cu(I) site on CeO_2(111). [a]

Element	Valence Electrons	Charge	Difference from the Reference	Charge Transfer
Ce	9.619	+2.3806	-0.0194 [b]	-0.524
O	7.202	-1.2017	-0.0017 [b]	-0.092
Cu	10.383	+0.617	+0.617 [c]	+0.617

[a] Charge Transfer is calculated based on the number of lost or gained electrons relative to the valence electron of neutral atoms (Ce: 12; O: 6 and Cu: 11). - denotes gaining electrons, while + is losing electrons. In total, 27 Ce gain 27 × - 0.0194 = - 0.524 and 54 O gain 54 × - 0.0017 = - 0.092. [b] Compared with pure CeO_2. [c] Compared with neutral Cu atom.

To simulate the electron transfer of atomic Cu(II) on CeO_2(111), we select the $[Cu(II)O_4]^{6-}$ site as the model, which is converted from the $[Cu(II)O_2(\eta^2\text{-}O_2)]^{4-}$ site at 453 K. More charges are donated by Cu (+ 1.074 vs + 0.617) while the lattice O also loses charges (+ 0.432). Ce and the 2 adsorbed O receive -0.316 and -1.190 charges, respectively (Fig. X2 and Table X3).

Therefore, the increase in the electron density of Ce gained from both Cu(I) and Cu(II) sites proves the role of Ce^{4+} as an electron acceptor for the atomic Cu(I/II) sites.

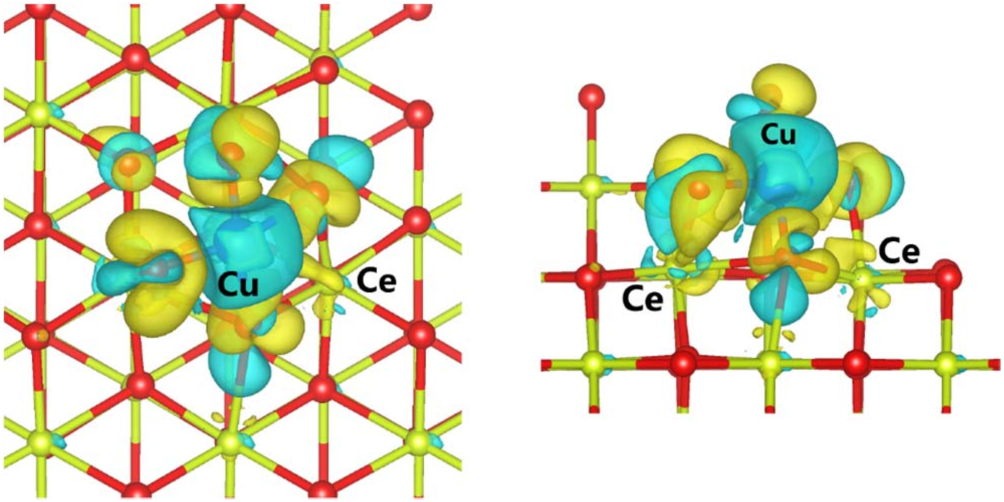

Figure X2. The CDD plot of atomic Cu(II) on CeO_2(111) with adsorbed O atoms. In the CDD plots, yellow isosurfaces denote electron gain and blue isosurfaces mean the electron lost.

Table X3. Bader charge analysis results of atomic Cu(II) site on CeO_2(111). [a]

Element	Valence Electrons	Charge	Difference from the Reference	Charge Transfer
Ce	9.612	+2.3883	-0.0117 [b]	-0.316
O	7.175	-1.1920	+0.008 [b]	+0.432
O_{ads}	6.595	-0.595	-0.595 [c]	-1.190
Cu	9.926	+1.074	+1.074 [d]	+1.074

[a] Charge Transfer is calculated based on the number of lost or gained electrons relative to the valence electron of neutral atoms (Ce: 12; O: 6 and Cu: 11). + denotes gaining electrons, while - is losing electrons. In total, 27 Ce gain 27 × - 0.0117 = - 0.316 and 54 O lose 54 × + 0.0008 = + 0.432. Two O_{ads} gain 2 × - 0.595 = - 1.190 in total. [b] Compared with pure CeO_2. [c] Compared with neutral O atom. [d] Compared with neutral Cu atom.

Supplementary Figs. 18, 19 and Tables 6-8 are added to show the CDD of Cu(I) and Cu(II) sites over CeO_2. The method of calculation is added to the supplementary materials.

The following sentences are added to the paragraph after Fig. 4.

"Bader charge analysis is calculated to analyse the charge transfer between Cu(I/II) sites and Ce^{4+} on the CeO_2 (111) surface. In both $[Cu(I)O_2]^{3-}$ and $[Cu(II)O_4]^{6-}$ sites, a charge transfer from Cu at the value of + 0.617 and + 1.074 is observed, respectively. Ce receives charge at the value of -0.524 and -0.316 for 27 Ce^{4+} ions that participate in the calculation, respectively (Supplementary Figs. 18, 19 and Tables 6-8). The increase of electron density of Ce in both models proves the role of Ce^{4+} as an electron acceptor for the atomic Cu(I/II) sites."

Question 2: The causality between the HOMO energy and the activity of the Cu sites are not clear. Considering the MvK mechanism is the most widely accepted mechanism (Chem. Rev. 113, 4391–4427 (2013), the highest activity of the atomic dispersed Cu(I/II) sites would be due to the observation that "CeO_2 is easier to be reduced under UHV with atomic Cu site (line 254-255)". To support the main conclusion of the current manuscript, the authors should provide more convincing experimental or theoretical investigations revealing the causality between the HOMO energy and the activity of the Cu sites.

Response 2: We thank the reviewer for the insightful comments on the role of HOMO energy and reaction activity. We performed additional NEXAFS measurement and CO temperature programmed reduction/desorption experiments to reinforce the credibility of this causal relationship.

The main conclusion of the manuscript is that low HOMO energy enables the selective O_2 adsorption and activation, which then improves the CO oxidation activity. CO oxidation over the Cu site on CeO_2 is a complicated process which involves the activation of CO and O_2

molecules, oxygen mobility and transfer in the CeO_2 lattice, and other elemental steps in the reaction coordinates. To justify the causality between HOMO energy and CO oxidation activity, activation CO and O_2, together with the oxygen transfer are discussed below.

1. The ability of atomic Cu (I/II) sites and their neighbouring Ce cations to activate O_2.

Upon O_2 adsorption on atomic Cu(I) site (1wt% CuO-CeO_2 reduced by CO), an electrophilic η^2-O_2 species is formed (Fig. 4c). In comparison, the adsorption of O_2 over CuO/Cu_2O clusters (20wt% CuO-CeO_2 reduced by CO) only forms nucleophilic lattice O^{2-} (Fig. X3). We hypothesise that the η^2-O_2 is favourable to be formed on atomic Cu(I) sites due to its lowered HOMO energy so electrons from the Cu HOMO tends to flow into the $\pi_g^*(2p)$ of O_2, and break only the O-O π bond. The HOMO energy is not high enough comparing with the $\sigma_u^*(2p)$ in O_2, thus the O-O σ bond is preserved. For Cu_2O and Cu clusters with much higher HOMO energy, the electrons on the Cu HOMO flow into both the $\pi_g^*(2p)$ and the $\sigma_u^*(2p)$, breaking the O=O σ and π bond and forming nucleophilic O^{2-}. In the CO oxidation, such electrophilic η^2-O_2 species will attract electrons from the CO, and thus improve the activity.

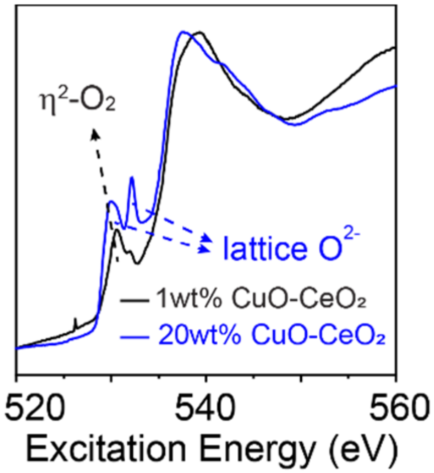

Figure X3. The O K-edge NEXAFS of different surface oxygen species on 1wt% CuO-CeO_2 and 20wt% CuO-CeO_2 under UHV after the adsorption of O_2 (1 mbar) at 298 K.

2. The ability of atomic Cu (I/II) sites to adsorb and activate CO.

In order to compare the difference in activating CO with atomic Cu sites and CuO clusters, we performed CO temperature programmed reduction (CO-TPR) and desorption (CO-TPD) with 1wt% CuO-CeO_2, 20wt% CuO-CeO_2 and pure CeO_2 (Fig. X4). The content of CO and CO_2 in the exhaust gas was quantified by IR detectors. To remove any potential carbon contamination, the sample (100 mg) was pre-oxidised in 100 mL/min 5% O_2/He at 673 K for 30 mins before cooling down to room temperature in He. For CO reduction, the sample was kept flowing with 2% CO/N_2 (100 mL/min) and heated up to 473 K at the rate of 5 K/min. The reduction peak of 20wt% CuO-CeO_2 is 451 K (Fig. X4a), which is similar to the reduction profile of CuO nanoparticles to metallic Cu (*J. Phys. Chem. C* **117**, 14588-14599 (2013)). The small amount of CO_2 released at low-temperature range comes from the reduction of more oxidative Cu sites on the surface of the CuO clusters. In comparison, the reduction peak of 1wt% CuO-CeO_2 is shifted to 380 K, indicating the atomic Cu(II) sites are

more active towards CO oxidation than the majority of the CuO sites on clusters. As unveiled by *operando* XAFS, the atomic Cu(I) sites will not be further reduced to metallic Cu (Fig. 5a-c, first column). Therefore, the tail of the CO_2 release profile is mainly contributed by the reduction of CeO_2 support, as the pure CeO_2 prepared by flame spray pyrolysis is gradually reduced above 400 K. Furthermore, the electron withdrawing effect of Ce^{4+} is enhanced for atomic Cu sites compared with CuO clusters, making it easier to reduce Cu(II) to Cu(I) with 1wt% CuO-CeO_2.

The CO-TPD experiments were performed to study the CO adsorption/desorption behaviour on Cu and identify the most oxidative Cu sites under insufficient CO environment. The sample adsorbed CO in 2% CO/N_2 (100 mL/min) at room temperature for 30 mins, before being blown with He (100 mL/min) for another 30 mins to remove physically adsorbed CO. The CO adsorbed sample was then heated up to 673 K in He with a rate at 5 K/min. Most of the desorbed CO have been oxidised to CO_2 as the amount of CO in the exhaust gas is negligible. Only a trace amount of CO is adsorbed on pure CeO_2, which is oxidised to CO_2 above 550 K (not shown here). The CO_2 desorption peak (374 K) from 1wt% CuO-CeO_2 is 6 K lower than the peak (380 K) from 20wt% CuO-CeO_2 (Fig. X4b). The 6 K difference is reproducible and solid. The lower CO_2 desorption temperature of 1wt% CuO-CeO_2 could either result from reduced CO desorption temperature or enhanced oxidation ability of the Cu-O species on the surface which are both beneficial to CO oxidation.

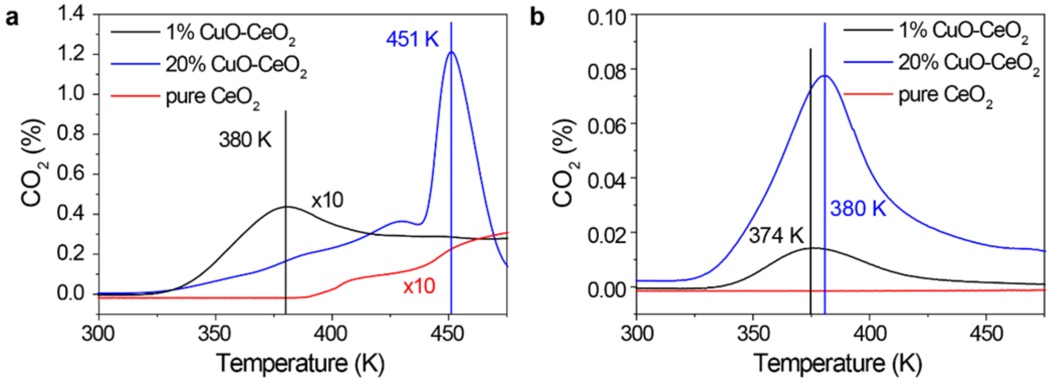

Figure X4. The evolution profile of CO_2 during the CO temperature programmed reduction (**a**) and desorption (**b**) of 1wt% CuO-CeO_2, 20wt% CuO-CeO_2 and pure CeO_2.

3. The oxygen transfer rate of the ceria modified by atomic Cu (I/II) sites.

We agree with the reviewer that the MvK mechanism is important for the CO oxidation over CeO_2. The indispensable role of Ce(III/IV) pair in the redox of atomic Cu sites (Supplementary Scheme 1) is validated by the re-oxidation of Cu(I) sites by Ce(IV) in He (Fig. 5 a-c, second column). The literature provided by the reviewer (Chem. Rev. 113, 4391–4427 (2013)) shows that a low valence state dopant can activate the adjacent lattice oxygen and reduce the oxygen vacancy formation energy (ΔE_v) of CeO_2, which then facilitate the transfer of oxygen species. Such oxygen vacancies can be correlated with the formation of Ce^{3+}. Thus, higher Ce^{3+} content suggests the easier formation of oxygen vacancy, which are resulted from the reduction of ΔE_v. We performed the XPS measurement of the 1wt% (Supplementary Fig. 21a) and 20wt% (Supplementary Fig. 21b) CuO-CeO_2 to quantify the content of Ce^{3+}. The results show 28.6% and 22.8% of Ce^{3+} for 1wt% and 20wt% (Table X4).

Thus, with a higher content of the Ce^{3+} associated with the presence of atomic Cu(I/II) sites, the higher oxygen transfer rate is predicted.

Table X4. Deconvolution results for the XPS of 1wt% and 20wt% CuO-CeO_2 catalysts.

				**1wt% CuO-CeO$_2$**			**20wt% CuO-CeO$_2$**		
**Species**	**Peak**	**label**	**Final state**	**BE [eV]**	**Area**		**BE [eV]**	**Area**	
**Ce (III)**	$3d_{5/2}$	v_0	Ce $3d^9 4f^2$ O $2p^5$	880.2	2.0%	28.6%	879.9	1.0%	22.8%
	$3d_{3/2}$	u_0		898.3	1.3%		898.0	0.7%	
	$3d_{5/2}$	v'	Ce $3d^9 4f^1$ O $2p^6$	884.3	15.1%		884.2	12.6%	
	$3d_{3/2}$	u'		902.6	10.1%		902.5	8.4%	
**Ce (IV)**	$3d_{5/2}$	v	Ce $3d^9 4f^0$ O $2p^6$	882.1	11.8%	71.4%	882.0	12.1%	77.2%
	$3d_{3/2}$	u		900.7	7.9%		900.6	8.1%	
	$3d_{5/2}$	v''	Ce $3d^9 4f^1$ O $2p^5$	888.5	15.5%		888.4	17.9%	
	$3d_{3/2}$	u''		907.0	10.4%		906.9	12.0%	
	$3d_{5/2}$	v'''	Ce $3d^9 4f^2$ O $2p^4$	898.2	15.5%		898.3	16.3%	
	$3d_{3/2}$	u'''		916.5	10.4%		916.6	10.9%	
**Cu**	$2p_{3/2}$	N/A	Cu $2p^5$ O $2p^6$	932.6	66.7%		932.7	66.7%	
	$2p_{1/2}$			952.5	33.3%		952.6	33.3%	

In conclusion, the atomic Cu sites on ceria not only promote the adsorption and activation of O_2, but also show advantages in the CO activation and the transfer of oxygen species in the ceria support. To establish the causal relations between HOMO energy and the CO oxidation activity, the turnover frequency (TOF) of the reaction is plotted as the function of the $1s \rightarrow 3d$ transition energy (Fig. X5). A negative relationship is observed, suggesting that low $1s \rightarrow 3d$ transition energy leads to high TOF.

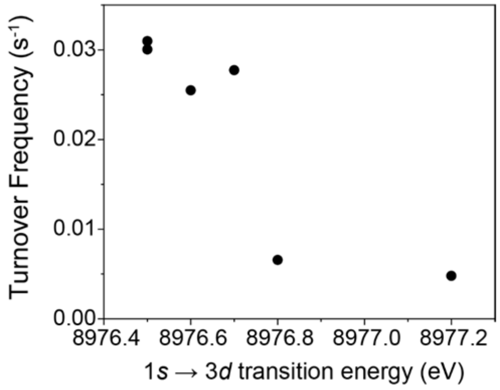

Figure X5. The CO oxidation TOF of various CuO-CeO_2 catalysts with different absorption energies of $1s \rightarrow 3d$ transition.

Supplementary Fig. 15 is added to compare the NEXAFS spectra on O K-edge of 1wt% and 20wt% $CuO-CeO_2$.

Supplementary Fig. 20 is added for the CO-TPR and CO-TPD.

Supplementary Table 9 is added to show the deconvolution results of XPS data to determine Ce^{3+} content.

CO-TPR and CO-TPD methods are added to the methods section.

The following sentence is added at the second paragraph before Fig. 4.

"Such η^2-O_2 is not observed in the 20wt% $CuO-CeO_2$, possibly due to the higher HOMO level of the CuO clusters which breaks the O=O double bond (Supplementary Fig. 15)."

The following sentences are added in the second paragraph after Fig. 4
"In addition to O_2, activation of CO and the mobility of lattice O in CeO_2 are also important for the CO oxidation. CO temperature programmed reduction and desorption are performed to compare the activation of CO with Cu single-site and clusters. The atomic Cu(II) sites are more active towards CO adsorption and oxidation than the majority of CuO sites on clusters as reflected by the shift of the reduction peak from 451 K to 380 K (Supplementary Fig. 20a). The Cu(II) site shows the main desorption peak at 374 K, which is 6 K lower than that of the CuO clusters (Supplementary Fig. 20b). Higher Ce^{3+} content[49] is found in the 1wt% $CuO-CeO_2$ comparing with 20wt% $CuO-CeO_2$ (Supplementary Fig. 21 and Table 9), which agrees with the shoulder band at 600 cm^{-1} in the Raman spectrum (Supplementary Fig. 13). According to the MvK mechanism, high Ce^{3+} content suggests more oxygen vacancy formation,[50] and thus improve the mobility of lattice O.[51] Therefore, the presence of the atomic Cu site promotes the O_2 and CO activation and the lattice O mobility, leading to higher CO oxidation activity compared with CuO clusters (Fig. 3c)."

Question 3: In line 74-75 (or Figure 1), the authors stated that "the surface atomic metal site has discrete electronic structures compared with the continuous band structures found in clusters." And line 82-84 the discrete electronic structures of Cu on Ag/Cu systems were mentioned again. Why are the discrete electronic structures important, which shall be emphasized?

Response 3: We thank the reviewer for the comments on the discrete electronic structures. The importance lies in 1) the structure and energy match between discrete orbitals and reaction molecules; 2) the ability to control and modify individual orbitals.

The possibility of achieving unprecedented reactivity and selectivity with single-site catalyst lies in its unique electronic structures. As illustrated in Fig. 1, the downward size effect makes Cu single sites exhibit discrete energy structure like a molecule. Therefore, the atomic

site interacts with reactants on the bases of the structure and energy match. For example, the highly diluted Cu_1Ag_{35} alloy shows additional π bonds with adsorbed O due to its free-atom-like narrow *d* states, resulting in a smaller activation barrier in methanol reforming compared with bulk Cu. (Ref. 29) The single-atom-layer PdCo alloy film exhibits splitting unoccupied *d* orbitals, leading to bulk-like stability and superior activity in ORR. (*Chem* **6**, 431-447 (2019).).

In addition, the discrete electronic structures can be more precisely modulated to meet the requirement of the desired reaction pathway, suppressing side reactions and thus enhancing selectivity in preferential oxidation of CO (*Nat. Chem.* **3**, 634-641 (2011).), hydroformylation of olefins (*Angew. Chem. Int. Ed.* **55**, 16054-16058 (2016); *Nat. Commun.* **7**, 14036 (2016).), semi-hydrogenation of acetylene (*ACS Catal.* **9**, 11030-11041 (2019).), selective hydrogenation of 1,3-Butadiene (*J. Am. Chem. Soc.* **137**, 10484-10487 (2015).) and chemoselective hydrogenation (*Nat. Commun.* **5**, 5634 (2014).).

Here in the Cu single-site over CeO_2 surface, the discrete orbitals of the Cu site lead to the formation of new molecular orbitals with O_2, forming the η^2-O_2 species. Such species is not observed on the CuO clusters, suggesting the importance of the discrete orbitals.

We add the following sentences to the second paragraph
"Their interactions with reactants are based on their structure and energy match."

"Thus, the ability to precisely control the electronic structures of atomic sites is important to achieve the desired reaction pathway."

Question 4: The authors assigned discrete HOMO as occupied 3*d* orbitals of Cu (Figure 1). However, the transitions of 1*s* → 3*d* shown in Figure 2e indicated that 3*d* orbitals are not fully occupied and/or not discrete. The authors should provide justification for their description of discrete HOMO.

Response 4: We thank the reviewer for the suggestions. The justification of 3*d* states of the atomic Cu site is presented below with an explanation of the discrete and occupied states. We performed additional High Energy Resolution Fluorescence Detected X-ray Absorption Near Edge Spectroscopy (HERFD-XANES) study to validate our conclusion.

Occupied state: Cu(II) site is $3d^9$ configuration. Despite the difference in coordination symmetry, there is always a half-filled orbital. Most of the Cu(II) species in this work are 4-fold coordinated as evidenced by EXAFS fitting. The d^9 electron configurations of Cu(II) complexes in a 4-fold coordinated crystal field with square planar and tetrahedral structure are shown in Figure X6. For both coordination structures, there is a half-filled orbital ($2b_{1g}$ or $2t_2$) originated from the $3d^9$ orbital of Cu(II). Therefore, the absorption energy of the transition 1*s* → 3*d* depends on the energy level of this half-empty orbital which is the only destination of the excited electron to 3*d* orbitals. We consider this orbital as the highest occupied molecular orbital (HOMO) or the singly occupied molecular orbital (SOMO).

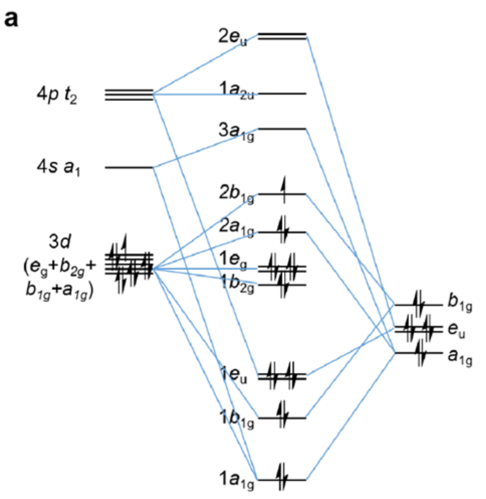

Figure X6. The molecular orbital diagrams of atomic 4-fold coordinated Cu(II) species with **a**, square planar and **b**, tetrahedral symmetries.

Discrete nature: The absorption of X-ray not only happens when the energy exactly matches the gap of the transition, but also occurs at both pre- and post-edge with relatively low absorption coefficient. Therefore, the XANES profile is a consecutive curve, which does not indicate the 3*d* orbitals are non-discrete. The absorption profile shows peaks at the different transition edges on its first derivative as the absorption coefficient maximised there. We have performed HERFD-XANES measurement, showing the details of such 1*s* → 3*d* transition (Fig. X7).

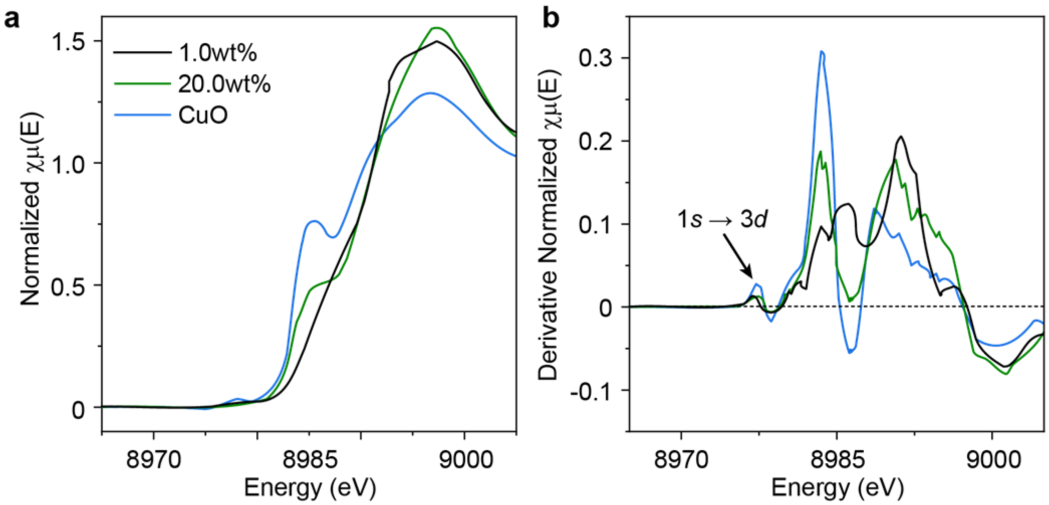

Figure X7. HERFD-XANES spectra (**a**) and the corresponding first derivatives (**b**) of 1wt%, 20wt% $CuO-CeO_2$ and CuO standard.

Supplementary Fig. 5e,f and Table 2 is updated for the HERFD-XANES study.

We add the following sentences to the paragraph before Fig. 2

"The Cu(II) site has the $3d^9$ configuration with four O^{2-} ligands. The absorption of 1*s* → 3*d* depends on the energy level of the half-empty orbital which is the only destination of the

excited electron to 3*d* orbitals. This is considered to be the HOMO or the singly occupied molecular orbital (SOMO)."

"High Energy Resolution Fluorescence Detected X-ray Absorption Near Edge Spectroscopy (HERFD-XANES) is performed to validate such change of 1*s* → 3*d*, *1s* → $4p_{xy}$ and *1s* → $4p_z$ energy from clusters to atomic sites, showing the similar trend (Supplementary Fig. 5e,f and Table 2)."

Question 5. In Figure 4, the authors assigned the pure Cu(II) species as $[Cu(II)O_4]^{6-}$, where all oxygen atoms become lattice O^{2-}. However, according to the DFT calculations shown in Supplementary Figure 15, this structure is 1.05 eV less stable than $[Cu(II)O_2(\eta^2\text{-}O_2)]^{4-}$. The authors should provide justification for the existence of the unstable Cu(II) species.

Response 5: We thank the reviewer for the comments on the stability of the Cu(II) species. We performed additional NEXAFS experiments to understand the stability of $[Cu(II)O_2(\eta^2\text{-}O_2)]^{4-}$ and $[Cu(II)O_4]^{6-}$ (Fig. X8).

The $[Cu(II)O_2(\eta^2\text{-}O_2)]^{4-}$ site is obtained after it is cooled down from 453 K to 298 K in 0.35 mbar O_2 and switched to UHV. The initial $[Cu(II)O_2(\eta^2\text{-}O_2)]^{4-}$ (black) is converted into $[Cu(II)O_4]^{6-}$ (blue) when the temperature is increased from 298 K to 453 K under UHV. When the temperature decreases back to 298 K, the original $[Cu(II)O_2(\eta^2\text{-}O_2)]^{4-}$ (red) site is observed again. This shows the reversible conversion between $[Cu(II)O_2(\eta^2\text{-}O_2)]^{4-}$ and $[Cu(II)O_4]^{6-}$ sites from 298 K to 453 K. As the most stable site at 298 K, $[Cu(II)O_2(\eta^2\text{-}O_2)]^{4-}$ should have lower energy than $[Cu(II)O_4]^{6-}$ site. This corresponds well with the DFT calculation of 1.05 eV difference. We admit that the DFT calculation is conducted at 0 K, which is different from the NEXAFS experiments.

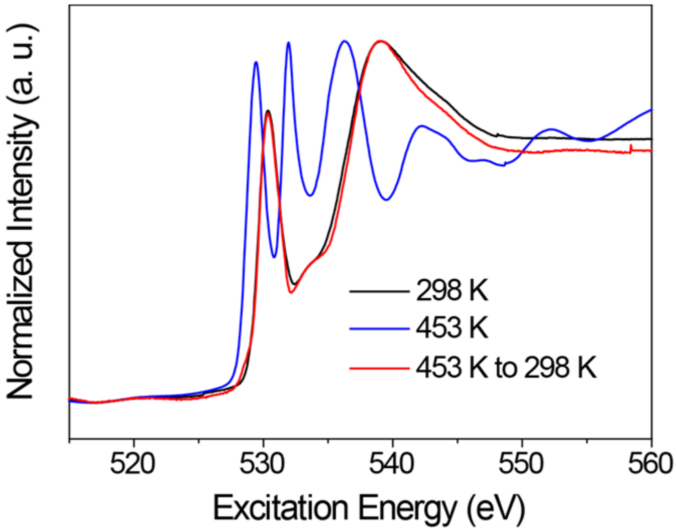

Figure X8. The NEXAFS of 1wt% $CuO\text{-}CeO_2$ on O K-edge under UHV, showing the reversible conversion between $[Cu(II)O_2(\eta^2\text{-}O_2)]^{4-}$ (298 K) and $[Cu(II)O_4]^{6-}$ (453 K).

Supplementary Fig. 17 is added for the additional NEXAFS experiments on the reversible

conversion of the O species.

We add the following sentences to the paragraph after Fig. 4.

"The $[Cu(II)O_4]^{6-}$ can be further converted back to $[Cu(II)O_2(\eta^2\text{-}O_2)]^{4-}$ by just reducing the temperature to 298 K, suggesting the reversible conversion between these two Cu(II) sites (Supplementary Fig. 17)."

Question 6. The selective activation of O_2 was not well defined and determined in this work. The selectivity can be among different activated oxygen species, like O^{2-}, O_2^-, O_2^{2-}. The selectivity also can be among the activation of O_2 and the other reactants.

Response 6: We thank the reviewer for pointing this out. In the manuscript, the selective activation at Cu single-site means that O_2 is selectively activated to electrophilic η^2-O_2 species at low temperature and nucleophilic O^{2-} species at high temperature. In comparison, the CuO clusters will mainly form nucleophilic O^{2-} species regardless of temperature. The η^2-O_2 can be either O_2^- or O_2^{2-}. The Raman shift at 830 cm^{-1} suggests that such η^2-O_2 should be the O_2^{2-} species.

We performed calculations on the change of charge on the adsorbed oxygen species. Upon the cleavage of the O-O bond, the charge per O_{ads} atom decreases from -0.22 to -0.595, suggesting the presence of O_2^{2-} species.

We rephrase the following sentence in the abstract
"*Operando* XAS, EPR, NAP-NEXAFS and DFT simulations are used to demonstrate that a $[Cu(I)O_2]^{3-}$ site selectively adsorbs molecular O_2, forming a rarely reported electrophilic η^2-O_2 species at 298 K."

We rephrase the following sentence in the paragraph before Fig. 1
"As part of our search for catalytic active centres in selective O_2 adsorption and activation towards electrophilic or nucleophilic oxygen species"

We add the following sentence with the calculated charge in the paragraph after Fig. 4

"The calculated charge per O_{ads} atom decreases from -0.22 to -0.595 (Supplementary Table 8)."

Minor points:

1.Line 61, "group 8" should be "group 10" or "group VIII".

We have changed it to group 10.

2.Line 69, "d9 and d10" should be "d10 and d9".

We have changed it to d^{10} and d^{9}.

3.Line 81, the author state that "the energy of the LUMO increases, weakening its propensity to form a bond with O_2". Because O_2 often plays as an oxidant, this statement should be revised.

We have deleted this sentence.

4.Line 93, Refs. 31 and 32 were cited for the $[Cu(II)O_2(\eta^2\text{-}O_2)]^{4-}$. However, both references are related to Ag rather than Cu. The proper references should be cited here.
Refs. 31 and 32 were cited for proving the electrophilic oxygen species formed on Ag-based catalysts are more suitable oxidants for the epoxidation of ethylene. The η^2-O_2 formed on atomic Cu(I) sites obtained in this work shows similar electrophilicity. Thus, it is hypothesised to improve the selectivity of oxidation reactions. We have moved these two references to the following sentence because they describe the electrophilic oxygen species.

"As part of our search for catalytic active centres in selective O_2 adsorption and activation towards electrophilic or nucleophilic oxygen species,[31,32]"

5.In Figure 2h, the smallest wt% is less than 0, which should be corrected.

We have corrected this mistake and upload a new Figure 2.

6.Line 163, the "tetragonally distorted octahedral" structure should be clarified.
The structure is indicated as a distorted octahedral due to the Jahn-Teller effect. The "tetragonally distorted octahedral" term is used to describe the structure resolved from signal A1. In this structure, the two possible anions are elongated from the z-axis. Such two anions at the z-axis cannot be detected *via* EXAFS, due to the long scattering length. Therefore, the EXAFS spectra only show four coordinated O ligands.

We have rephrased the following sentence:
"isolated atomic Cu(II) site in tetragonally distorted octahedral due to Jahn-Teller effect"

7.Line 243, the Raman band located around 830 cm^{-1} in Supplementary Fig. 13 is associated with the η^2-O_2. What is the more intense shoulder band around 600 cm^{-1}?
The intense shoulder band around 600 cm^{-1} is attributed to the presence of Ce^{3+}, as the ionic radius of Ce^{3+} is much larger than that of Ce^{4+} (*J. Colloid Interface Sci.* **389**, 16-22 (2013).). The absence of this feature for Ce^{3+} on the spectra of 20wt% CuO-CeO_2 and pure CuO-CeO_2 validates the role of atomic Cu sites in reducing the oxygen vacancy formation energy of ceria.

We add the following sentence to the second paragraph before Fig. 4

“The shoulder band around 600 cm^{-1} is attributed to Ce^{3+},[48] which is absent for the Raman spectra of 20wt% $CuO-CeO_2$ and pure CeO_2.”

8.In Supplementary Materials line 208, the method for PDOS was provided. However, there is no PDOS reported in this work.

We have removed the method for PDOS. Sorry for the mistake.

9.In Supplementary Materials line 369, is it “CeO_2(111)” or “CeO_2(110)”?

Thanks for pointing it out. We have corrected to CeO_2(110).

Reviewer #2 (Remarks to the Author):

Overall comments: The authors describe an interesting study on the activation of dioxygen at isolated Cu single sites supported on ceria by a multitechnique approach combining operando XAS, EPR, NAP-NEXAFS and DFT simulations. They provided evidence for the presence of an electrophilic $[Cu(II)O_2(\eta^2\text{-}O_2)]^{4-}$ species as a key intermediate state that should account for the high catalytic activity and selectivity of related oxidation reactions. In particular, the authors proposed the concept of using electronic structures to control the catalytic performance. Overall, these results allow to gain a fundamental insight into the structural, electronic and reactive properties of ceria-supported Cu single site catalysts. This work could be interesting to a broad readership. However, I cannot recommend this manuscript for publication in Nature Commun. in its current form. I have the following major concerns:

We thank this referee for the positive comments that “these results allow to gain a fundamental insight into the structural, electronic and reactive properties of ceria-supported Cu single site catalysts” and other constructive suggestions. We address all the major concerns below to explain the results and justify the main conclusion.

Question 1: With regards to the stable structure of Cu single atoms on ceria, the DFT simulations are insufficiently described. It is known that the morphologies of different ceria surfaces play a crucial role in the interaction between metal and support. As mentioned in the paper, the surface Ce^{3+} species are involved in the activation of dioxygen. In this context, the oxygen vacancy formation energy depends strongly on the orientation of ceria surfaces (see e.g., Chem. Rev., 2013, 113, 3949). The surface structures of the ceria NPs should be taken into account.

Response 1: We thank the reviewer for the comments on the DFT simulations on the stable structure of atomic Cu sites and the effect from the morphologies of different ceria surfaces. We agree that different exposed facets of ceria play a crucial role in the interaction between metal and support, especially for the ceria with regular morphologies. We attempt to correlate the activity of atomic Cu sites and the ceria exposed facets from both theoretical and experimental aspects.

1. DFT simulations of atomic Cu sites on $CeO_2(111)$, $CeO_2(110)$, and $CeO_2(100)$.

The interactions between atomic Cu sites and CeO_2 surface is facet dependent. To prove this, we conducted DFT simulations of the stable structure of atomic Cu sites on $CeO_2(111)$, $CeO_2(110)$ and $CeO_2(100)$ surfaces. The 2-fold coordinated Cu sites on $CeO_2(111)$ and $CeO_2(110)$ surfaces show similar stable structures and evolution of the Cu-O species (Supplementary Figs. 11 and 25, Tables 4 and 5). In comparison, the most stable structure of the Cu site coordinated with 4 surface lattice O^{2-} on $CeO_2(100)$ (Fig. X9). As a result, oxygen can only be physically adsorbed on this Cu site with a distance at 3.01 Å (Fig. X9c), whereas the Cu(I) site on $CeO_2(111)$ or $CeO_2(110)$ surface chemically adsorb O_2 with an adsorption energy of -0.68 eV and -1.25 eV respectively.

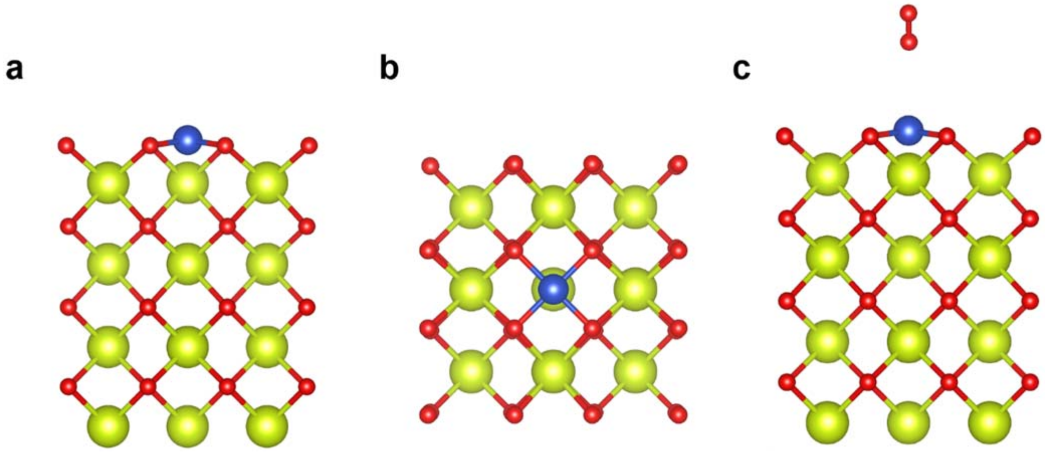

Figure X9. Optimised structures of atomic Cu sites on CeO_2 (100) surface based on DFT+U simulations. **a**, Side view of a 4-fold coordinated Cu site. **b**, Top view of a 4-fold coordinated Cu site. **c**, Side view of a physically adsorbed O_2 molecule on a 4-fold coordinated Cu site.

2. Experimental evidence of higher Ce^{3+} content in ceria modified by atomic Cu sites.

It is generally accepted that Ce^{3+} ions are accompanied by the oxygen vacancies according to the review provided by the referee (*Chem. Rev.* **113**, 3949-3985 (2013)). Thus, higher Ce^{3+} content indicates more oxygen vacancy formation, which is resulted from the reduction of ΔE_v. We performed the XPS measurement of the 1wt% (Supplementary Fig. 21a) and 20wt% (Supplementary Fig. 21b) CuO-CeO_2 to quantify the content of Ce^{3+}. Additional XPS measurement for pure CeO_2, spherical CeO_2 and 1wt% CuO on spherical CeO_2 was conducted to study the influence of ceria morphologies (Fig. X10). The results show 28.6%, 22.8%, 22.4% of Ce^{3+} for 1wt% CuO-CeO_2, 20wt% CuO-CeO_2 and pure CeO_2, respectively (Table X5). In comparison, the spherical CeO_2 and spherical CeO_2 with 1wt% CuO have same Ce^{3+} content (19.8%, Table X5), which is lower than that of the ceria used in this work. Thus, with a higher content of the Ce^{3+} associated with the presence of atomic Cu(I/II) sites, the higher oxygen transfer rate is predicted. The intense shoulder band around 600 cm^{-1} on the Raman spectrum of 1wt% CuO-CeO_2 (Supplementary Fig. 13) is attributed to the presence of Ce^{3+}, as the ionic radius of Ce^{3+} is much larger than that of Ce^{4+} (*J. Colloid Interface Sci.* **389**, 16-22 (2013)). The absence of this feature for Ce^{3+} on the spectra of 20wt% CuO-CeO_2 and pure CuO-CeO_2 validates the role of atomic Cu sites in reducing the oxygen vacancy formation energy of ceria.

According to the calculated energy of oxygen vacancy formation (ΔE_v) in the literature (Chem. Rev. 113, 4391–4427 (2013)), it is relatively hard to transfer oxygen to Cu sites from $CeO_2(111)$ compared with $CeO_2(110)$ or $CeO_2(100)$. The atomic Cu(I/II) site as a low-valence dopant can activate the adjacent lattice oxygen on CeO_2 and reduce the ΔE_v of the support, which then facilitates the transfer of oxygen species.

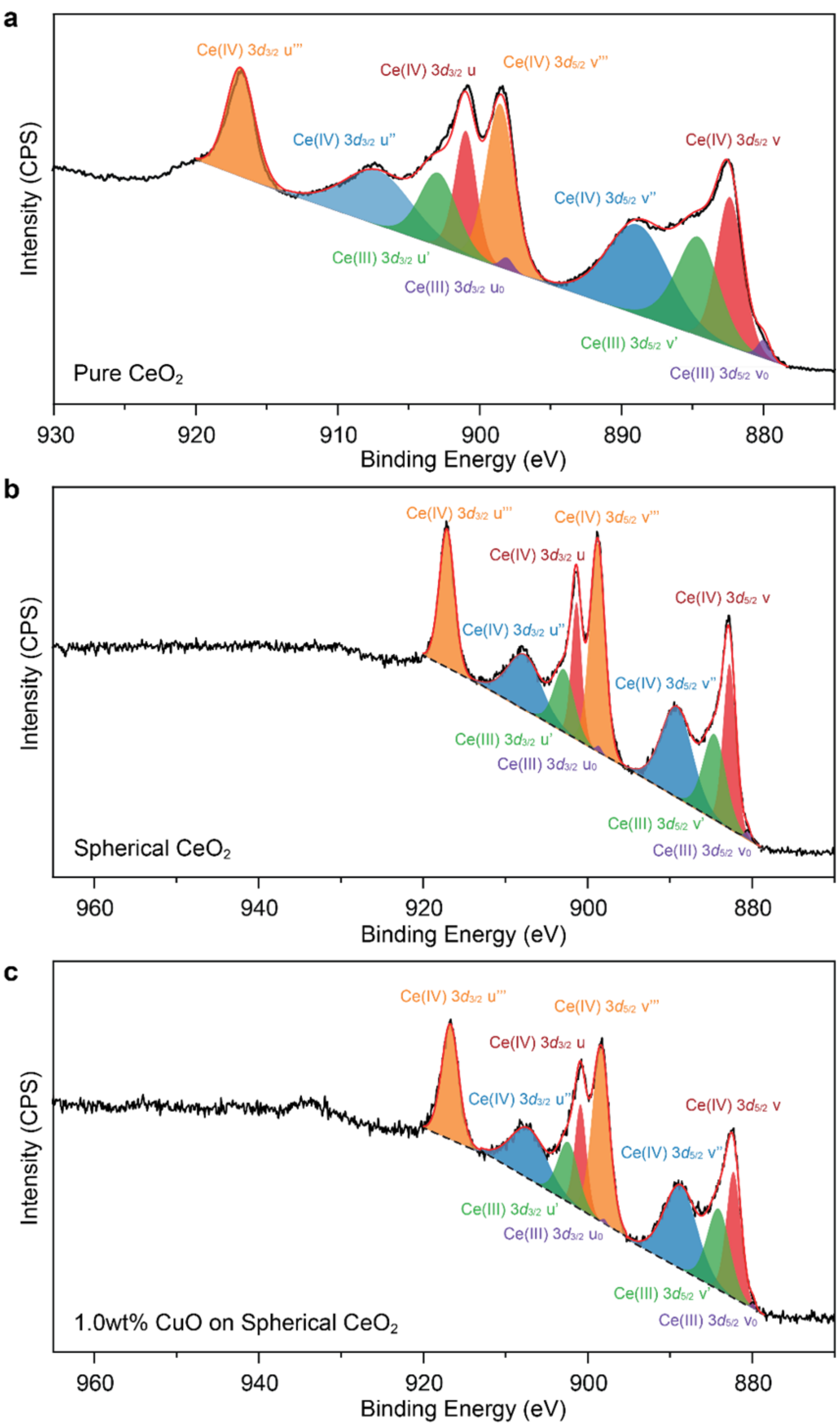

Figure X10. XPS for **a**, Pure CeO_2, **b**, Spherical CeO_2 and **c**,1wt% CuO on spherical CeO_2

Table X5. Deconvolution results for the XPS of pure CeO_2 and 1wt% CuO-CeO_2.

Samples	Ce(III)				Ce(IV)					
	v_0	u_0	v'	u'	v	u	v''	u''	v'''	u'''
	$3d_{5/2}$	$3d_{3/2}$	$3d_{5/2}$	$3d_{3/2}$	$3d_{5/2}$	$3d_{3/2}$	$3d_{5/2}$	$3d_{3/2}$	$3d_{5/2}$	$3d_{3/2}$
Pure CeO_2	0.7%	0.5%	12.7%	8.5%	13.1%	8.8%	18.0%	12.0%	15.4%	10.3%
	22.4%				77.6%					
1wt% CuO-CeO_2	2.0%	1.3%	15.1%	10.1%	11.8%	7.9%	15.5%	10.4%	15.5%	10.4%
	28.6%				71.4%					
20wt% CuO-CeO_2	1.0%	0.7%	12.6%	8.4%	12.1%	8.1%	17.9%	12.0%	16.3%	10.9%
	22.8%				77.2%					
Spherical CeO_2	0.3%	0.2%	11.6%	7.7%	12.6%	8.4%	16.8%	11.2%	18.7%	12.5%
	19.8%				80.2%					
1wt% CuO on Spherical CeO_2	0.2%	0.1%	11.7%	7.8%	11.8%	7.8%	17.5%	11.7%	18.8%	12.6%
	19.8%				80.2%					

3. Facet dependent CO oxidation activity.

We loaded the atomic Cu site over CeO_2 with different morphologies. To ensure a similar dispersion of Cu species on the ceria surface, the loading of CuO is controlled at 0.5 wt%. It is found that the CuO loaded on cubic CeO_2(100) surface shows the lowest activity in CO oxidation, whereas the activity for CuO on ceria prepared *via* flame spray pyrolysis (FSP) is the highest one (Fig. X11). The CuO-CeO_2 FSP has irregular shapes (Fig. 2), so we hypothesise that the surface is a combination of (111), (110) and (100) facets.

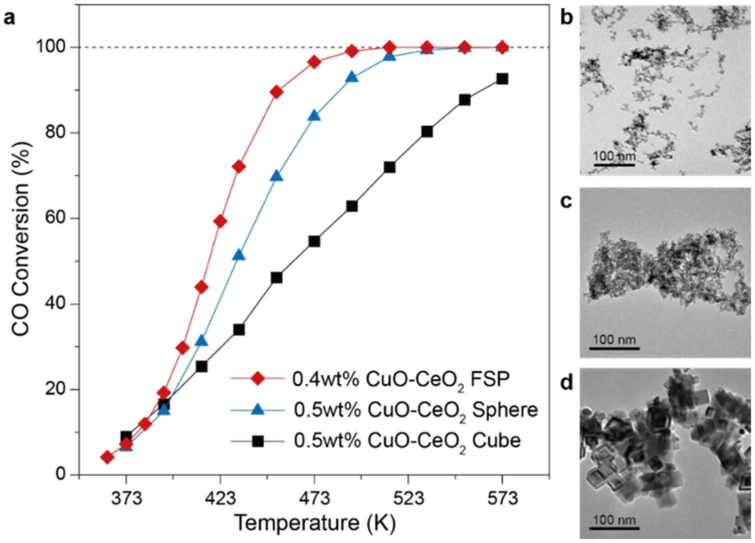

Figure X11. a, The reactivity of CO oxidation catalysed by 0.4 wt% CuO-CeO_2 synthesised by FSP, 0.5 wt% CuO on cubic CeO_2 and 0.5 wt% CuO on spherical CeO_2, respectively. TEM images of ceria support with various morphologies; **b**, irregular shape of FSP ceria; **c**, spherical ceria; **d**, cubic ceria.

In conclusion, we have observed facet dependent activity in CO oxidation. The main reasons

are hypothesised as: 1) The most stable Cu structures on CeO_2(111), CeO_2(110) are 2-fold coordinated whereas the structure on CeO_2(100) is 4-fold coordinated. 2) Chemical O_2 adsorption of Cu site over CeO_2(111), CeO_2(110) promotes the formation of η^2-O_2 species. Such adsorption is not possible with Cu site over CeO_2(100). 3) Enhanced Ce^{3+} content is found in the 1wt% CuO-CeO_2 synthesised by FSP compared with pure CeO_2, whereas the spherical CeO_2 shows the same lower Ce^{3+} content.

Supplementary Fig. 14 is added to identify the stable structure of atomic Cu sites on CeO_2(100).

Supplementary Table 6 is added to show the deconvolution results of XPS data to determine Ce^{3+} content.

The following sentences are added to the paragraph before Fig. 4

In comparison, Cu coordinates with four surface lattice O^{2-} on CeO_2(100) (Supplementary Fig. 14). O_2 can only be physically adsorbed on such site with a distance at 3.01 Å based on DFT simulations.

The following sentences are added in the second paragraph after Fig. 4
"Higher Ce^{3+} content[49] is found in the 1wt% CuO-CeO_2 comparing with 20wt% CuO-CeO_2 (Supplementary Fig. 21 and Table 9), which agrees with the shoulder band at 600 cm^{-1} in the Raman spectrum (Supplementary Fig. 13). According to the MvK mechanism, high Ce^{3+} content leads to more oxygen vacancy formation,[50] and thus improve the mobility of lattice O.[51] Thus, the presence of the atomic Cu site promotes the O_2 and CO activation and the lattice O mobility, leading to higher CO oxidation activity compared with CuO clusters (Fig. 3c)."

Question 2: In addition to Cu adatoms, single Cu atoms also could be stabilized by substituting a cerium atom on the surface and/or in the bulk under oxidative conditions.

Response 2: We thank the reviewer for the comment on the other possible locations of the atomic Cu sites, especially the consideration of its dynamic evolution under oxidative conditions.

If Cu replaces a cerium atom on the surface or in the bulk, the Cu-O distance should be similar or slightly smaller to that of the Ce-O distance, which is 2.38 Å. This is due to the lattice geometry and the strains that apply to the Cu atoms. We performed EXAFS analysis and fitting to identify the Cu-O distance under various oxidation conditions. The length is between 1.90 and 1.95 Å (Fig. 5 and supplementary table 10). Therefore, it is not likely that Cu will be inside Ce lattice to replace the Ce atom.

The following sentence is added to the fourth paragraph before Fig. 5

The Cu-O bond distance agrees well with the DFT model of the $[Cu(I)O_2]^{3-}$ site on CeO_2 surface (Supplementary Tables 4 and 5). As the Cu-O bond is not elongated to match the Ce-O bond length (2.38 Å) in bulk CeO_2,[52] such atomic Cu sites are not likely to be stabilised by substituting Ce atoms in CeO_2 lattice.

Question 3: A number of groups have reported that metal single sites are not active for oxidation reactions (see e.g. Nat. Commun. 2019, 10: 3808; Nat. Mater. 2019, 18, 746). A recent work showed that the ultrafine Cu clusters (bilayer) on ceria account for the high catalytic activity for the water gas shift reaction (Nat. Catal. 2019, 2, 334).

Response 3:

We thank the reviewer for the recommended literature. It is acknowledged that atomically dispersed active sites do not always exhibit the highest activity.

In the water gas shift reaction, the highly active bilayer Cu clusters consist of Cu^+-O_v-Ce^{3+} interfacial perimeter, where Cu^+ site activates CO and O_v-Ce^{3+} activates H_2O. (*Nat. Catal.* **2**, 334-341 (2019).) The top layer Cu^0 atoms only serve as the coordination ligand and are not directly involved in the catalysis. Although this reaction needs bifunctional active sites, the activation of CO only relies on the Cu^+ atoms at the Cu-O-Ce interfacial perimeter, which highly resembles the structure of atomic Cu(I) sites on ceria studied in our work. The reactivity of single sites in oxidation might be further improved if a more suitable structure can be adopted. The enhanced activity of isolated active sites in oxidation and the comparison of various turnover frequency (TOF) with Cu-based and Pt-based catalysts are discussed below (Table X6).

In general, the atomic Cu site in the manuscript has high activity (TOF) compared with the Cu or Pt sites in the literature (Table X6). The Pt-O-Pt dimers that built from isolated Pt sites on CeO_2 exhibit 100-1000 times TOF (197×10^{-2} vs 1.7×10^{-2} s^{-1}) and much lower activation energy (40 vs 86 $kJ\cdot mol^{-1}$) in CO oxidation (*Nat. Commun.* **10**, 3808 (2019).) This work also claims that dinuclear Pt-O-Pt structure is not always more active than atomic Pt species in oxidation since the exceptionally active Pt-O-Pt dimers in this work rely on special coordination environment and high dispersion on ceria. Similar activity improvement of atomically dispersed Pt-TiO_2 catalysts is also found when the isolated Pt single-site (0.14×10^{-2} s^{-1}, 78 $kJ\cdot mol^{-1}$) was converted into an atomic Pt-OH site (0.49×10^{-2} s^{-1}, 48 $kJ\cdot mol^{-1}$) after being reduced at 723 K in 5% H_2/Ar (*Nat. Mater.* **18**, 746-751 (2019), Ref. 54). The following work on similar Pt-CeO_2 catalysts finds that the isolated Pt species with low loadings exhibited high resistance to sintering up to 773 K in H_2 as these Pt species preferentially occupy the most thermodynamically stable adsorption sites on ceria (*J. Am. Chem. Soc.* **142**, 169-184 (2019)). Therefore, the catalytic behaviour of metal single sites relies highly on the inherent surface property of supports and local coordination environment. The most active structure could vary with different combinations of active metal centres and support materials.

The atomic Cu(I) sites studied in this work are favourable in the formation of an electrophilic η^2-O_2 species (Fig. 4c), which is rarely reported with other Cu-based catalysts. We hypothesise that such electrophilic η^2-O_2 species can attract electrons from the CO, and thus

improve the activity of CO oxidation.

In CO oxidation, we demonstrate that the isolated Cu(I)/(II) sites are ten times more active than CuO clusters in terms of TOF (Fig. 3c). The TOF in CO oxidation of atomic Cu sites (2.48×10^{-2} s^{-1}) is also higher than that of Cu-based catalysts in the literature and is comparable with that of the isolated Pt sites on CeO_2 (2.43×10^{-2} s^{-1}, Table X6, *Nat. Mater.* **18**, 746-751 (2019)). Below 1wt% CuO loading, the CuO-CeO_2 catalysts exhibit similar TOF and *E*a, indicating identical isolated active sites with the same intrinsic activity. When the CuO loading is above 1wt%, the catalytic activity of the CuO-CeO_2 catalysts declines as reflected by the decreased TOF and increased *E*a. The continuous decrease of the normalised activity suggests that the cluster sites are generally less active compared with isolated Cu sites.

Table X6. Comparison of the CO oxidation activity of various active metal sites.

<table>
<tr><th rowspan="2">Active Sites</th><th colspan="2">Gas</th><th rowspan="2">Conversion</th><th rowspan="2">WHSV [a]
($mL\cdot g_{cat}^{-1}\cdot h^{-1}$)</th><th rowspan="2">TOF at
($x10^{-2}$ s^{-1})</th></tr>
<tr><th>[CO] vol%</th><th>[O_2] vol%</th></tr>
<tr><td>Atomic Cu sites on CeO_2 [b]</td><td rowspan="2">1%</td><td rowspan="2">10%</td><td>20.9%</td><td rowspan="2">120,000</td><td>2.48</td></tr>
<tr><td>Cu clusters on CeO_2 [c]</td><td>4.0%</td><td>0.47</td></tr>
<tr><td>12mol% CuO on CeO_2 [d]</td><td>1%</td><td>1%</td><td>50.0%</td><td>120,000</td><td>1.01</td></tr>
<tr><td>$Cu_{0.1}CeO_{2-x}$ [e]</td><td>1%</td><td>2.5%</td><td>10.0%</td><td>78,000</td><td>0.16</td></tr>
<tr><td>9mol% CuO on CeO_2 [f]</td><td>1%</td><td>10%</td><td>35.0%</td><td>60,000</td><td>0.47</td></tr>
<tr><td>Isolated Pt sites on CeO_2 [g]</td><td>0.4%</td><td>10%</td><td>12.6%</td><td>200,000</td><td>2.43</td></tr>
<tr><td>Pt_{iso}/TiO_2 reduced at 523 K [h]</td><td rowspan="2">1%</td><td rowspan="2">1%</td><td>0.03%</td><td rowspan="2">480,000</td><td>0.14</td></tr>
<tr><td>Pt_{iso}/TiO_2 reduced at 723 K [h]</td><td>0.11%</td><td>0.49</td></tr>
<tr><td>Pt_1/CeO_2-a [i]</td><td rowspan="2">0.1%</td><td rowspan="2">5%</td><td>0.8%</td><td rowspan="2">2,400,000</td><td>1.7</td></tr>
<tr><td>Pt-O-Pt/CeO_2-a [i]</td><td>91.9%</td><td>197</td></tr>
</table>

[a] WHSV = Weight hourly space velocity per gram of catalysts. [b] Reactivity of 1wt% CuO-CeO_2 at 373 K in this work. [c] Reactivity of 20wt% CuO-CeO_2 at 373 K in this work. [d] The conversion data at 373 K read from the figure of *J. Catal.* **246**, 52-59 (2007). [e] The conversion data at 373 K read from the figure of *J. Am. Chem. Soc.* **136**, 17193-17200 (2014). [f] The conversion data at 373 K read from the figure of *ACS Appl. Mater. Interfaces* **7**, 23538-23544 (2015). [g] The conversion data at 373 K read from the figure of *Science* **358**, 1419-1423 (2017). [h] The reactivity data obtained at 423 K in *Nat. Mater.* **18**, 746-751 (2019), data at 373 K is not shown by the authors. [i] The reactivity data obtained at 423 K shown in *Nat. Commun.* **10**, 3808 (2019), the reactivity of Pt_1/CeO_2-a at 373 K is too low for comparison with Pt-O-Pt/CeO_2-a.

Supplementary Table 11 is added to compare the reactivity in CO oxidation of various active

metal sites.

Supplementary Note 1 is added to discuss the comparison between Cu-based and Pt-based catalysts in CO oxidation.

The following sentence is added to the paragraph before Fig. 5

"The comparison among the TOFs obtained with various Cu-based and Pt-based catalysts in CO oxidation shows that the atomic Cu sites in 1wt% $CuO-CeO_2$ is more active than the reported Cu clusters catalysts and the activity is comparable with that of the isolated Pt sites on CeO_2[45] (Supplementary Table 11)."

Question 4: For the CO oxidation reaction, a synergistic effect should be more important than the activation of single reactant, i.e., a site cooperation allowing the activation of both CO and O_2.

Response 4: We thank the reviewer for the insightful comments on the synergistic effect on the activity in CO oxidation. We performed additional NEXAFS and CO temperature programmed reduction/desorption experiments to study the synergistic effect between Cu and CeO_2 in this reaction. It is shown that the same Cu site can activate both O_2 and CO, as well as promoting the oxygen mobility and transfer in the CeO_2 lattice.

1. Activation of O_2.

Upon O_2 adsorption on atomic Cu(I) site (1wt% $CuO-CeO_2$ reduced by CO), an electrophilic η^2-O_2 species is formed (Fig. 4c). In comparison, the adsorption of O_2 over CuO/Cu_2O clusters (20wt% $CuO-CeO_2$ reduced by CO) only forms nucleophilic lattice O^{2-} (Fig. X3). We hypothesise that the η^2-O_2 is favourable to be formed on atomic Cu(I) sites due to its lowered HOMO energy so electrons from the Cu HOMO tends to flow into the $\pi_g^*(2p)$ of O_2, and break only the O-O π bond. The HOMO energy is not high enough comparing with the $\sigma_u^*(2p)$ in O_2, thus the O-O σ bond is preserved. For Cu_2O and Cu clusters with much higher HOMO energy, the electrons on the Cu HOMO flow into both the $\pi_g^*(2p)$ and the $\sigma_u^*(2p)$, breaking the O=O σ and π bond and forming nucleophilic O^{2-}. In the CO oxidation, such electrophilic η^2-O_2 species will attract electrons from the CO, and thus improve the activity.

2. Activation of CO.

In order to compare the difference in activating CO with atomic Cu sites and CuO clusters, we performed CO temperature programmed reduction (CO-TPR) and desorption (CO-TPD) with 1wt% $CuO-CeO_2$, 20wt% $CuO-CeO_2$ and pure CeO_2 (Fig. X4). The content of CO and CO_2 in the exhaust gas was quantified by IR detectors. To remove any potential carbon contamination, the sample (100 mg) was pre-oxidised in 100 mL/min 5% O_2/He at 673 K for 30 mins before cooling down to room temperature in He. For CO reduction, the sample was kept flowing with 2% CO/N_2 (100 mL/min) and heated up to 473 K at the rate of 5 K/min. The reduction peak of 20wt% $CuO-CeO_2$ is 451 K (Fig. X4a), which is similar to the reduction profile of CuO nanoparticles to metallic Cu (*J. Phys. Chem. C* **117**, 14588-14599 (2013)). The small amount of CO_2 released at low-temperature range comes from the reduction of more oxidative Cu sites on the surface of the CuO clusters. In comparison, the

reduction peak of 1wt% $CuO-CeO_2$ is shifted to 380 K, indicating the atomic Cu(II) sites are more active towards CO oxidation than the majority of the CuO sites on clusters. As unveiled by *operando* XAFS, the atomic Cu(I) sites will not be further reduced to metallic Cu (Fig. 5a-c, first column). Therefore, the tail of the CO_2 release profile is mainly contributed by the reduction of CeO_2 support, as the pure CeO_2 prepared by flame spray pyrolysis is gradually reduced above 400 K. Furthermore, the electron withdrawing effect of Ce^{4+} is enhanced for atomic Cu sites compared with CuO clusters, making it easier to reduce Cu(II) to Cu(I) with 1wt% $CuO-CeO_2$.

The CO-TPD experiments were performed to study the CO adsorption/desorption behaviour on Cu and identify the most oxidative Cu sites under insufficient CO environment. The sample adsorbed CO in 2% CO/N_2 (100 mL/min) at room temperature for 30 mins, before being blown with He (100 mL/min) for another 30 mins to remove physically adsorbed CO. The CO adsorbed sample was then heated up to 673 K in He with a rate at 5 K/min. Most of the desorbed CO have been oxidised to CO_2 as the amount of CO in the exhaust gas is negligible. Only a trace amount of CO is adsorbed on pure CeO_2, which is oxidised to CO_2 above 550 K (not shown here). The CO_2 desorption peak (374 K) from 1wt% $CuO-CeO_2$ is 6 K lower than the peak (380 K) from 20wt% $CuO-CeO_2$ (Fig. X4b). The 6 K difference is reproducible and solid. The lower CO_2 desorption temperature of 1wt% $CuO-CeO_2$ could either result from reduced CO desorption temperature or enhanced oxidation ability of the Cu-O species on the surface which are both beneficial to CO oxidation.

3. Promoting oxygen mobility.

Oxygen vacancies are important for the oxygen transfer in the CeO_2 lattice, which promotes the CO oxidation. Such oxygen vacancies can be correlated with the formation of Ce^{3+}. Thus, higher Ce^{3+} content suggests the easier formation of oxygen vacancy, which are resulted from the reduction of the formation energy of oxygen vacancy. We performed the XPS measurement of the 1wt% (Supplementary Fig. 21a) and 20wt% (Supplementary Fig. 21b) $CuO-CeO_2$ to quantify the content of Ce^{3+}. The results show 28.6% and 22.8% of Ce^{3+} for 1wt% and 20wt%, respectively (Table X4). Thus, with a higher content of the Ce^{3+} associated with the presence of atomic Cu(I/II) sites, the higher oxygen transfer rate is predicted.

In Conclusion, the atomic Cu sites on ceria not only promote the adsorption and activation of O_2, but also show advantages in the CO activation and the transfer of oxygen species in the ceria support.

Supplementary Fig. 15 is added to compare the NEXAFS spectra on O K-edge of 1wt% and 20wt% $CuO-CeO_2$.

Supplementary Fig. 20 is added for the CO-TPR and CO-TPD.

Supplementary Table 9 is added to show the deconvolution results of XPS data to determine Ce^{3+} content.

CO-TPR and CO-TPD methods are added to the methods section.

The following sentences are added at the second paragraph after Fig. 4

"In addition to O_2, activation of CO and the mobility of lattice O in CeO_2 are also important for the CO oxidation. CO temperature programmed reduction and desorption are performed to compare the activation of CO with Cu single-site and clusters. The atomic Cu(II) sites are active towards CO adsorption and oxidation than the majority of CuO sites on clusters as reflected by the shift of the reduction peak from 451 K to 380 K (Supplementary Fig. 20a). The Cu(II) site shows the main desorption peak at 374 K, which is 6 K lower than that of the CuO clusters (Supplementary Fig. 20b). Higher Ce^{3+} content[49] is found in the 1wt% CuO-CeO_2 comparing with 20wt% CuO-CeO_2 (Supplementary Fig. 21 and Table 9), which agrees with the shoulder band at 600 cm^{-1} in the Raman spectrum (Supplementary Fig. 13). According to the MvK mechanism, high Ce^{3+} content suggests more oxygen vacancy formation,[50] and thus improve the mobility of lattice O.[51] Therefore, the presence of the atomic Cu site promotes the O_2 and CO activation and the lattice O mobility, leading to higher CO oxidation activity compared with CuO clusters (Fig. 3c)."

Reviewers' comments:

Reviewer #1 (Remarks to the Author):

In this revised manuscript, the authors have addressed some of my issues. I now feel that this work has the potential to be published in Nature Communications. However, some issues in my previous comment are still unsolved. Particularly, the causality between the HOMO energy and the activity of the Cu sites is still unclear. As a result, although the selective activation of O_2 over the atomic Cu(I/II) site on CeO_2 is observed, the most important conclusion in this work, i.e., "low HOMO energy enables the selective O_2 adsorption and activation, which then improves the CO oxidation activity", is not convincing. Thus, the authors should address the following points before a recommendation can be made.

1. The author claimed that "η^2-O_2 is not observed in the 20wt% CuO-CeO_2, possibly due to the higher HOMO level of the CuO clusters which breaks the O=O double bond". This statement is just a speculation without any convincing evidence. Because calculations of the energy level for HOMO at first-principles levels are routine nowadays, the authors should provide direct evidence for their statement of the effect of low HOMO energy on CO oxidation activity with proper modeling (see below).
2. In the revised manuscript, the authors tried to estimate the charge transfer among Cu, O and Ce for the $[Cu(I)O_2]^{3-}$ and $[Cu(II)O_4]^{6-}$ sites via the Bader charge analysis. However, the authors put a neutral Cu atom on the $CeO_2(111)$ surface to model $[Cu(I)O_2]^{3-}$. In this model, the formal oxidation state of Cu is 0 rather than the expected +1, which is not a proper electronic structure for $[Cu(I)O_2]^{3-}$. Thus, the authors should use a proper model for $[Cu(I)O_2]^{3-}$. Furthermore, the CuO_2 cluster was put on the $CeO_2(111)$ surface to model $[Cu(II)O_4]^{6-}$, where two O-O bonds have to be formed to keep the Cu(II) oxidation state. The justification of the formation of O-O bonds in $[Cu(II)O_4]^{6-}$ should be provided. Overall, the electron withdrawing effect of Ce^{4+} to Cu(I/II) is still ambiguous and should be more properly addressed.
3. The relationship in Figure X5 is interesting. Nevertheless, it is neither in the revised manuscript nor in the revised Supplementary Materials. I strongly recommend that the authors shall add Figure X5 and its related discussions in the revised manuscript.

Reviewer #2 (Remarks to the Author):

The authors have adequately responded to my comments and I consider that the manuscript is appropriate for publication.

Response to Referee 1:

To the comments of Reviewer 1

Overall comments: In this revised manuscript, the authors have addressed some of my issues. I now feel that this work has the potential to be published in Nature Communications. However, some issues in my previous comment are still unsolved. Particularly, the causality between the HOMO energy and the activity of the Cu sites is still unclear. As a result, although the selective activation of O_2 over the atomic Cu(I/II) site on CeO_2 is observed, the most important conclusion in this work, i.e., "low HOMO energy enables the selective O_2 adsorption and activation, which then improves the CO oxidation activity", is not convincing. Thus, the authors should address the following points before a recommendation can be made.

We thank this referee for further comments on the theoretical calculations to support the causality between the HOMO energy and the activity of the Cu sites. We address all the issues below to explain the results and justify the main conclusion.

Question 1: The author claimed that "η^2-O_2 is not observed in the 20wt% CuO-CeO_2, possibly due to the higher HOMO level of the CuO clusters which breaks the O=O double bond". This statement is just a speculation without any convincing evidence. Because calculations of the energy level for HOMO at first-principles levels are routine nowadays, the authors should provide direct evidence for their statement of the effect of low HOMO energy on CO oxidation activity with proper modeling (see below).

Response 1:
We thank the reviewer for the comments on the justification of the relationship between HOMO level and CO oxidation activity. We admit that their different behaviour towards η^2-O_2 species is not well explained in the previous revision (Supplementary Fig. 15).

Here we first compare the HOMO energy of both Cu(I) and Cu(II) species, then calculate the O=O dissociation over single-site and clusters, and finally discuss the relation between CO oxidation activity and HOMO energy.

First, lower HOMO levels are confirmed for both Cu(I) and Cu(II) single sites compare with Cu_2O and CuO clusters. For the Cu(II) species, XANES spectra of Cu(II) single-sites show a shift of 1*s* → 3*d* transition to lower energies, suggesting the lower HOMO energy compared with CuO clusters (8976.5 eV *vs* 8977.1 eV, Fig. 2e). For the Cu(I) species, because the 3*d* orbital of Cu(I) is fully filled, we cannot use XANES to determine the HOMO level of Cu(I) single-site and Cu_2O clusters (Supplementary Fig. 28). Therefore, we perform additional calculations to compare the HOMO level of the Cu(I) single site and Cu_2O(111) surface at the first-principles level (Table Y1), in which the Cu_2O(111) is used here to represent the Cu_2O clusters over CeO_2. The simulations show that the Cu(I) single-site on CeO_2(111) has significantly lower valence band

maximum compared with $Cu_2O(111)$ surface (-0.9451 eV vs -0.2244 eV). Such a 0.7207 eV difference from calculations of Cu(I) species is very close to that of the experimentally measured difference in $1s \rightarrow 3d$ transition energy of Cu(II) species (0.6 eV, Fig. 2e). In conclusion, both Cu(I) and Cu(II) single-sites on CeO_2 have lower HOMO energy compared with Cu clusters.

Table Y1. The valence band maximum (VBM) of Cu(I) single-site and $Cu_2O(111)$ surface.

Model	VBM (eV)
$Cu_2O(111)$	-0.2244
Cu(I) single-site on $CeO_2(111)$	-0.9451

Second, we calculate the dissociation of O_2 over $Cu_2O(111)$ surface (Fig. Y1) and compare the result to the O_2 dissociation over Cu single-site. The dissociation of O_2 on $Cu_2O(111)$ surface requires a lower energy barrier (1.13 eV vs 1.41 eV). The final state of the fully dissociated O atoms is 0.3 eV more stable than the initial undissociated O_2, whereas the final sate of oxygen on Cu single-site is 1.05 eV less stable than its initial state (Supplementary Fig. 16). These results suggest the dissociation of O_2 on $Cu_2O(111)$ is easier than that on Cu single-sites, confirming the statement that “clusters break the O=O double bond”.

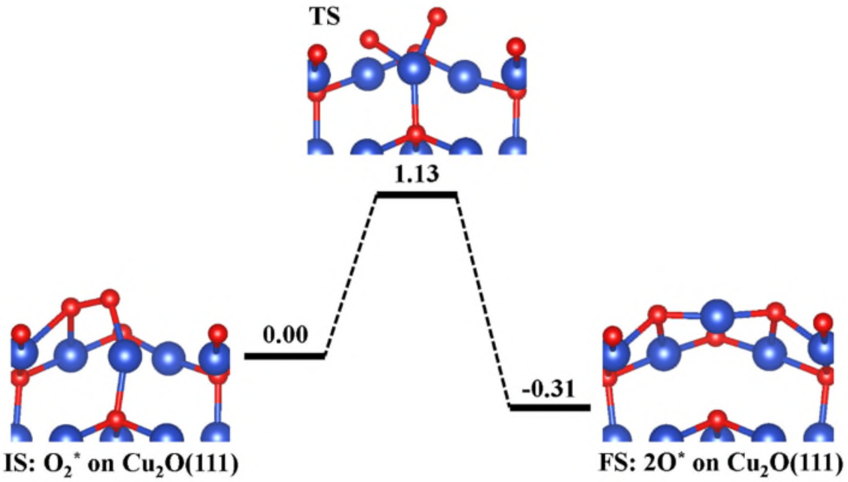

Figure Y1 | Reaction pathway for the decomposition of adsorbed O_2 molecule on $Cu_2O(111)$. Initial state (IS): adsorbed O_2 molecule on $Cu_2O(111)$; transition state (TS): decomposing state of O_2; final state (FS): double adsorbed O atoms on $Cu_2O(111)$. Colour schemes: blue, Cu; red O.

Third, we discuss the effect of lowered HOMO in Cu(I)/(II) single-sites in the O=O

dissociation. The XANES and DFT calculation show the electronic structure of Cu (I)/(II) single-sites in terms of lower HOMO level. DFT calculation, Raman spectroscopy and NEXAFS (Supplementary Figs. 13 and 18) show the performance of Cu (I)/(II) single-sites, which requires high energy barrier in breaking the O=O bond in comparison with clusters. Such structure-performance relationship is observed.

Forth, we discuss the effect of lowered HOMO in Cu(I)/(II) single-sites in CO oxidation. The η^2-O_2 species which is only observed on Cu single-sites with lower HOMO energy is electrophilic.(Ref 31,32 in text) Since the oxygen activation plays a key role in CO oxidation, such electrophilic η^2-O_2 species will facilitate the electrons flown from CO to oxygen, leading to higher CO oxidation activity. In addition, the lowered HOMO energy also results in the weakened adsorption strength of CO (Supplementary Fig. 21), which is beneficial for CO oxidation.

In conclusion, Cu(I)/(II) single-sites have lowered HOMO levels compared with Cu_2O/ CuO clusters, respectively, as confirmed by DFT calculation and XAFS. DFT, Raman and NEXAFS study show that the O=O dissociation is easier over clusters. η^2-O_2 species formation and enhanced CO oxidation activity are then attributed to the lowered HOMO levels. Consequently, a negative correlation between HOMO energy level and TOF of CO oxidation is observed (Fig. 3g).

Supplementary Table 12 and Fig. 17 are added to compare the HOMO energy and O_2 decomposition pathways of Cu(I) single-site and Cu_2O cluster.

The following sentences are added to the end of the third paragraph before Fig. 5.

"DFT calculation shows that the Cu(I) single-site on CeO_2(111) has significantly lower valence band maximum compared with Cu_2O(111) surface (-0.9451 eV vs -0.2244 eV, Supplementary Table 12). Such a 0.7207 eV difference from calculations of Cu(I) species is very close to that of the experimentally measured difference in 1*s* → 3*d* transition energy of Cu(II) species (0.6 eV, Fig. 2e). This result further validates that both Cu(I) and Cu(II) single-sites on CeO_2 have lower HOMO energy compared with Cu clusters."

The following sentences are added to the paragraph after Fig. 4.

"In comparison, decomposition of O_2 on Cu_2O(111) surface requires an energy barrier of 1.13 eV (Supplementary Fig. 17). The final state with fully dissociated O atoms is 0.3 eV more stable than the initial undissociated O_2, whereas the final sate of oxygen on Cu single-site is 1.05 eV less stable than its initial state. These results suggest the dissociation of O_2 on Cu_2O clusters is easier than that on Cu single-sites."

Question 2: In the revised manuscript, the authors tried to estimate the charge transfer among Cu, O and Ce for the $[Cu(I)O_2]^{3-}$ and $[Cu(II)O_4]^{6-}$ sites via the Bader charge

analysis. However, the authors put a neutral Cu atom on the CeO_2(111) surface to model $[Cu(I)O_2]^{3-}$. In this model, the formal oxidation state of Cu is 0 rather than the expected +1, which is not a proper electronic structure for $[Cu(I)O_2]^{3-}$. Thus, the authors should use a proper model for $[Cu(I)O_2]^{3-}$. Furthermore, the CuO_2 cluster was put on the CeO_2(111) surface to model $[Cu(II)O_4]^{6-}$, where two O-O bonds have to be formed to keep the Cu(II) oxidation state. The justification of the formation of O-O bonds in $[Cu(II)O_4]^{6-}$ should be provided. Overall, the electron withdrawing effect of Ce^{4+} to Cu(I/II) is still ambiguous and should be more properly addressed.

Response 2:
We thank the reviewer for the comments on the further justification of electron withdrawing effect of Ce^{4+} to Cu(I/II) sites. These comments include two sub-questions, so we split our answer into two parts.

Part 1, answer to: '*However, the authors put a neutral Cu atom on the CeO_2(111) surface to model $[Cu(I)O_2]^{3-}$. In this model, the formal oxidation state of Cu is 0 rather than the expected +1, which is not a proper electronic structure for $[Cu(I)O_2]^{3-}$. Thus, the authors should use a proper model for $[Cu(I)O_2]^{3-}$.*'

We thank the reviewer for the comments on the justification of proper modelling for the Bader charge analysis.

The Bader charge analysis in VASP requires an electrically neutral model, therefore we cannot put a Cu^+ ion on CeO_2 to model $[Cu(I)O_2]^{3-}$ due to the method limitation. Although other simulation methods like Gaussian can assign the oxidation state of the metal centre as +1, they are commonly used for the molecular model with limited atoms and not applicable in the simulation of crystalline metal oxides. There usually are two approaches to increase the oxidation state of the metal centre during the VASP simulation. First, a positive charge can be quantitatively introduced to the model before the starting guess of the simulation, but an equivalent negative charge field must be uniformly applied to the model to neutralise the charge. This model is unrealistic in heterogeneous catalysis. Second, ligands with strong electron negativity can be introduced to the metal centre to increase its oxidation state. In our simulation model, oxygen atoms, which are available from CeO_2 lattice and gas feed, are used as ligands to control the oxidation state of Cu. The initial neutral Cu atom is clearly positively charged after it coordinated with two lattice oxygen atoms in CeO_2, which fits well with the coordination structure of the $[Cu(I)O_2]^{3-}$ single-site identified by *operando* XAFS (Fig. 5, Supplementary Fig. 19 and Tables 7, 11). In order to simulate the experimentally observed $[Cu(II)O_4]^{6-}$ single-site, we added two additional oxygen atoms on Cu to make the oxidation state of Cu single-site as close to +2 as possible (Supplementary Fig. 20 and Table 8).

To evaluate the influence of CeO_2 on the electron density of Cu ions in the $[Cu(I)O_2]^{3-}$ and $[Cu(II)O_4]^{6-}$ single-sites, we further select the pure Cu_2O(111) and CuO(111)

surface as the Cu(I) and Cu(II) models to represent clusters. Those two models are also formed with Cu(0) atoms and O atoms. The Bader charge of $[Cu(I)O_2]^{3-}$, $[Cu(II)O_4]^{6-}$, $Cu_2O(111)$ and CuO(111) models are then calculated in terms of the difference from neutral Cu atom. In our previous manuscript, the calculated Bader charges are + 0.617 and + 1.074 for $[Cu(I)O_2]^{3-}$ and $[Cu(II)O_4]^{6-}$, respectively (Table Y2). Here, the Bader charges of $Cu_2O(111)$ and CuO(111) are +0.495 and +1.001, respectively (Table Y2). The Bader charge of single-sites is +0.122 and +0.063 higher than those of the clusters. Comparing the difference the model structure and considering the significant electron transferred from Cu single-sites to Ce (Supplementary Tables 6-8), the increased positive charge of Cu single-sites should result from the electron withdrawing effect from Ce^{4+} for both Cu(I) and Cu(II) single-sites.

Part 2, answer to: *'Furthermore, the CuO_2 cluster was put on the $CeO_2(111)$ surface to model $[Cu(II)O_4]^{6-}$, where two O-O bonds have to be formed to keep the Cu(II) oxidation state. The justification of the formation of O-O bonds in $[Cu(II)O_4]^{6-}$ should be provided.'*

There are two types of Cu(II) single-sites identified in our work, i.e. $[Cu(II)O_2(\eta^2\text{-}O_2)]^{4-}$ and $[Cu(II)O_4]^{6-}$. The former has one O-O bond and the latter doesn't have O-O bond (Supplementary Fig. 26). Therefore, we guess the "two O-O bonds have to be formed to keep the Cu(II) oxidation state" mentioned by the reviewer is either two Cu-O bonds or two Ce-O bonds that are formed when putting the Cu-O_2 cluster on the $CeO_2(111)$ surface.

In the model to simulate the $[Cu(II)O_4]^{6-}$ site, there are four Cu-O bonds in total to stabilise the structure and maintain the +2 oxidation states. Two O atoms in Cu-O bond are provided by CeO_2 support. The additional Cu-O bonds are coming formed the Cu-O_2 cluster. This structure is coming from the adsorption of O_2 on $[Cu(I)O_2]^{3-}$ site with subsequent O=O dissociation, which is experimentally confirmed by *in situ* XAFS and NAP-NEXAFS studies. So the two O atoms provided by CeO_2 support are inherited from the $[Cu(I)O_2]^{3-}$ structure (adding Cu atom on CeO_2 surface) whereas the other two Cu-O bonds are introduced by the adsorption of molecular O_2 which is available in the reaction gas.

For the Ce-O bonds, the theoretical calculation shows the formation of additional two Ce-O bonds in $[Cu(II)O_4]^{6-}$ structure origins from the cleavage of the O-O bond in $[Cu(II)O_2(\eta^2\text{-}O_2)]^{4-}$ (Supplementary Fig. 26), forming two additional Cu-O-Ce bridges. This result suggests Ce can help stabilise the negative charge of the two adsorbed O atoms in the Cu-O_2 cluster. As a result, the newly formed Cu-O-Ce bridges give similar signals to that of lattice oxygen species in the O K edge NAP-NEXAFS (Fig. 4d, right).

Table Y2. The Bader charge analysis results of Cu(I) and Cu(II)

Species	Model	Bader Charge	Difference
Cu(I)	$Cu_2O(111)$	+0.495	+0.122
	Cu(I) single-site on $CeO_2(111)$	+0.617	
Cu(II)	CuO(111)	+1.001	+0.063
	Cu(II) single-site on $CeO_2(111)$	+1.074	

In summary, the electron withdrawing effect of Ce^{4+} to Cu(I/II) is further validated by the increased positive Bader charges of both $[Cu(I)O_2]^{3-}$ and $[Cu(II)O_4]^{6-}$ single-sites compared with their corresponding pure copper oxide, which are all calculated based on the same neutral Cu atom as the initial state.

Supplementary Table 9 is added to show the Bader charge analysis of pure $Cu_2O(111)$ and CuO(111) surface.

The following sentences are added to the paragraph after Fig. 4.

"Based on the same calculations parameters for the Cu single-sites, the calculated Bader charges of Cu_2O (111) and CuO (111) surface are +0.495 and +1.001, respectively (Supplementary Table 9). Compared with pure copper oxides, both Cu(I) and Cu(II) single-sites on CeO_2 are more positively charged."

The following sentences are added to the method of calculation in the supplementary materials.

"The $CeO_2(111)$ surface is selected as the representative support for the Cu single-sites. To simulate the Cu(I/II) single-sites, two models are constructed based on their structures in the forms of $[Cu(I)O_2]^{3-}$ and $[Cu(II)O_4]^{6-}$, which are identified by *in situ* XAFS (Fig. 5). In the model for $[Cu(I)O_2]^{3-}$ site, an initial neutral Cu atom is put on $CeO_2(111)$ surface and coordinates with two lattice oxygen atoms in CeO_2. As revealed by the NAP-NEXAFS, the $[Cu(I)O_2]^{3-}$ site can adsorb one O_2 molecule and evolve into the $[Cu(II)O_4]^{6-}$ site (Fig. 4). In the model for $[Cu(II)O_4]^{6-}$ site, there are four Cu-O bonds in total to stabilise the structure and maintain the +2 oxidation states. The two O atoms provided by CeO_2 support are inherited from the $[Cu(I)O_2]^{3-}$ structure (adding Cu atom on CeO_2 surface) whereas the other two Cu-O bonds are introduced by the

adsorption of molecular O_2 which is available in the reaction gas. There are four Ce-O-Cu bridges. The calculation shows the formation of additional two Ce-O bonds in $[Cu(II)O_4]^{6-}$ structure origins from the cleavage of the O-O bond in $[Cu(II)O_2(\eta^2\text{-}O_2)]^{4-}$ (Supplementary Fig. 26). As a result, the newly formed Cu-O-Ce bridges give similar signals to that of lattice oxygen species in the O K edge NAP-NEXAFS (Fig. 4d, right)."

Question 3: The relationship in Figure X5 is interesting. Nevertheless, it is neither in the revised manuscript nor in the revised Supplementary Materials. I strongly recommend that the authors shall add Figure X5 and its related discussions in the revised manuscript.

Response 3:

We thank the reviewer for this comment. Figure X5 has been added to Fig. 3g in the manuscript to illustrate the correlation between TOF of CO oxidation and X-ray absorption energies of 1*s* → 3*d* transition.

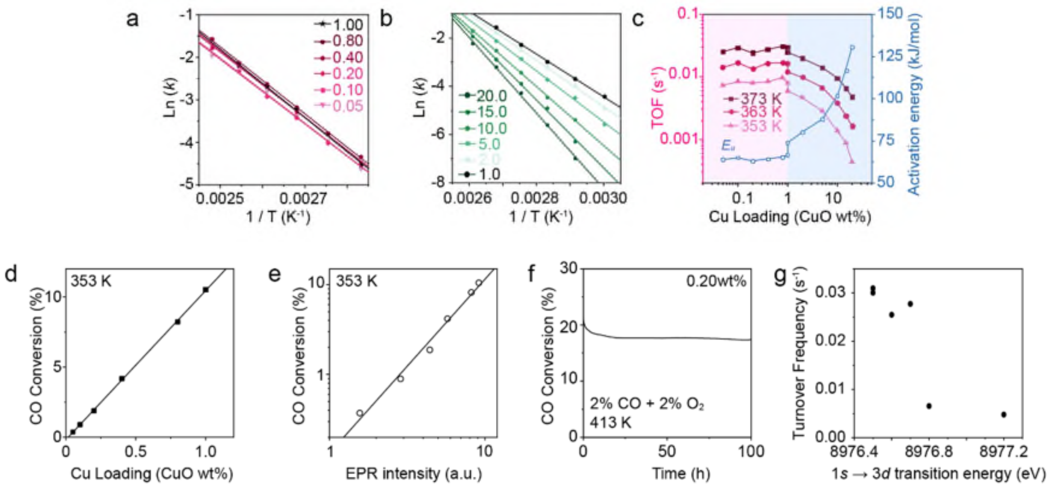

Figure 3 | Catalytic evaluation of Cu species in CO oxidation. a, Arrhenius plots at WHSV = 750,000 $mL_{CO}\cdot h^{-1}\cdot g_{CuO}^{-1}$ for Cu species ranging from 0.1 to 1wt%. **b,** Arrhenius plots at WHSV = 120,000 $mL_{CO}\cdot h^{-1}\cdot g_{CuO}^{-1}$ for Cu species ranging from 1 to 20wt%.**c**, TOF and E_a as function of Cu loading. WHSV = 750,000 $mL_{CO}\cdot h^{-1}\cdot g_{CuO}^{-1}$ and 120,000 $mL_{CO}\cdot h^{-1}\cdot g_{CuO}^{-1}$ were used below and above 1wt% CuO loading, as indicated in the pink and blue areas, respectively. **d**, Below 1wt% CuO loading, CO conversion as a function of CuO loading amount at 363 K. **e**, Below 1wt% CuO loading, CO conversion as a function of the EPR intensity of atomic Cu(II) site at 363 K. **f**, On stream stability test for 0.20wt% CuO-CeO_2 at 1,500,000 $mL_{CO}\cdot h^{-1}\cdot g_{CuO}^{-1}$ and 413 K. **g**, The correlation of the CO oxidation TOF of CuO-CeO_2 catalysts against their absorption energies of 1*s* → 3*d* transition.

To discuss this relationship, we add the following sentences in the paragraph before Fig. 3.

“The HOMO energy levels, which are consistent with the absorption energies of 1*s* → 3*d* transition in the Cu K edge XANES, are negatively correlated with TOF in CO oxidation activity (Fig. 3g). The lower HOMO energy level of Cu single-sites may lead to enhanced competence in O_2 activation, which is then investigated *via* a series of *in-situ* characterisation techniques below.”

REVIEWERS' COMMENTS:

Reviewer #1 (Remarks to the Author):

The authors have made the appropriate modifications. Publication is recommended.
